# Neural vs Neuromorphic Interfaces: Where Are We Standing?

**DOI:** 10.1021/acs.chemrev.4c00862

**Published:** 2025-09-23

**Authors:** Daniela Rana, Natalia Babushkina, Martina Gini, Alejandra Flores Cáceres, Hangyu Li, Vanessa Maybeck, Valeria Criscuolo, Dirk Mayer, Marcello Ienca, Simon Musall, Viviana Rincon Montes, Andreas Offenhäusser, Francesca Santoro

**Affiliations:** † Institute for Biological Information Processing-Bioelectronics, IBI-3 Forschungszentrum Juelich, 52428, Germany; ‡ Neuroelectronic Interfaces, Faculty of Electrical Engineering and IT, RWTH Aachen, Aachen 52074, Germany; § Institute for History and Ethics of Medicine, 9184 Technical University of Munich (TUM), Ismaniger Str. 22, Munich 81675, Germany

## Abstract

Neuromorphic interfaces represent a transformative frontier in neural engineering, enabling seamless communication between the nervous system and external devices through biologically inspired computing architectures. These systems offer promising avenues for diagnosing and treating neurological disorders by emulating the brain’s computational strategies. Neural devices, including sensors and stimulators, monitor or modulate neural activity, playing a pivotal role in deciphering brain function and neuropathologies. Yet, clinical translation remains limited due to persistent challenges such as foreign body responses, low signal-to-noise ratios, and constraints in real-time data processing. Recent breakthroughs in neuromorphic hardware, neural recording, and stimulation technologies are addressing these challenges, paving the way for more adaptive and efficient brain-machine interfaces and neuroprosthetics. This review highlights the emerging class of neurohybrid interfaces, where neuromorphic systems might be integrated to enhance bidirectional neural communication. It emphasizes novel material strategies engineered for seamless neural interfacing and their incorporation into advanced neuromorphic chip architectures capable of real-time signal processing and closed-loop feedback. Furthermore, this review explores cutting-edge neuromorphic biointerfaces and evaluates the technological, biological, and ethical challenges involved in their clinical deployment. By bridging materials science, neuroscience, and neuromorphic engineering, these systems hold the potential to redefine the landscape of neurotechnology.

## Graphical abstract

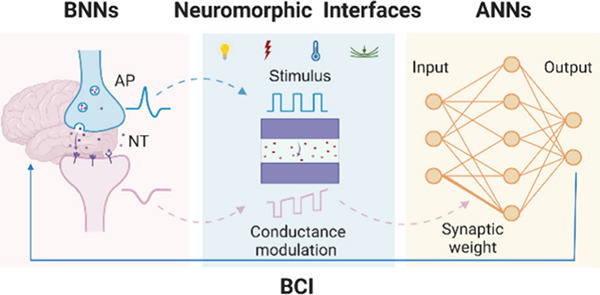

## 1 Introduction

The brain is an extraordinarily complex and efficient system, capable of sensing, processing, and transmitting stimuli while maintaining all vital functions on about 20 W of power.^1^ This stems from its dynamic structure, consisting of billions of neurons connected by trillions of synapses.^2^ The brain’s high plasticity supports the reshaping of these connections, enabling efficient processing of new information and propagation of signals based on learning processes. This relies on intricate functional connections across large neural networks, making the brain robust and capable of flexibly reconfiguring to optimize performance and processing.^3,4^

The nervous system possesses remarkable cognitive abilities, with a precise network of interconnected neurons. However, pathological conditions can severely affect people’s daily life, highlighting the demand for advanced technologies crucial for exploring brain function and understanding neuropathological processes. Following the discovery of EEG signals in the 1920s using scalp electrodes, various Neural interfaces have been developed to facilitate interaction with the brain.^5^ This area has garnered considerable interest within the scientific community for manipulating, monitoring, and restoring neuronal networks and their functionality.^6–8^ Two main pathways have been defined for the evolution of neural interfaces: BCIs or *i* BCIs. The former is a neural interface that captures neural activity in a safe and noninvasive way, while the latter, primarily known as BMIs,^9^ use more invasive approaches to achieve high-resolution acquisition of signals. BCIs and BMIs therefore present different balances in the quality of the neural communication achieved vs the clinical risk and ethical considerations.^9^

Neural devices refer to technologies used to interact with or monitor the nervous system, including sensors, stimulators, and recording equipment. These devices can measure or influence neural activity, such as brain waves or nerve signals, and are used in medical applications, like prosthetics, and neuromodulation therapies.

From a modern clinical perspective, both BCI (e.g., EEG^10^) and BMI (e.g., Utah array^11,12^ ) technology is experiencing rapid advances offering new neurorehabilitation methods and enabling individuals with disabilities to interact with the external environment by decoding signals from neural devices. Despite these advancements, challenges such as low SNR,^13^ immune responses in neural tissue for invasive approaches, limited functionality,^14^ and restricted data processing capabilities have hindered clinical translation. Thus, developing multifunctional neural devices with sensitivity to single-cell activity, excellent biocompatibility, and fast processing capabilities is relevant for diagnosing and treating nervous system diseases.^15^

Neural devices offer a valuable means to investigate the connections between neuron firing and synaptic transmission and hold promise for diagnosing and treating neurological disorders like epilepsy and Alzheimer’s disease. To achieve these goals, it is essential to develop neural devices with high spatiotemporal resolution.^16^

On the other hand, with the advent of the Turing test in the 1950s, initial ideas about artificial intelligence research sprouted, leading to the emergence of technologies like deep learning and big data computing.^17^ Then, in the 1970s, BCIs, and later in the early 2000s BMIs emerged alongside advances in neuroscience and AI,^18^ and today, the integration of AI with brain science has accelerated the development of neuromorphic devices and the rapid growth of hardware and software architectures for ANNs.^16^ In this context, the concept of a BNI has been introduced, leveraging neuromorphic components to enhance noninvasive and invasive brain-computer communication. These systems integrate sensing, processing, and actuation capabilities, offering a more energy-efficient and biologically realistic approach to traditional neural interfaces that rely on conventional CMOS-based systems.^19^

Innovative devices have been created to also simulate and emulate diverse biological neurons, providing potential substitutes for impaired sensory organs.

Neuromorphic systems mimic the topology and/or the functionality of biological neural networks in order to address some limitations of traditional technologies at the level of interfacing of biological tissues or computational paradigms for information processing. In turn, the data from a neural interface could be processed directly with a neuromorphic architecture and in a closed-loop process *ad hoc* endorsement signal could be sent back to the nervous system for stimulation, actuation and prosthetics.

### 1.1 Interfacing the Brain

A neural interface is defined as a platform that can interact with the nervous system, enabling communication between neurons and external devices (Figure 1). This communication typically involves recording and/or stimulating neural activity. Designing these interfaces begins with identifying brain patterns to control devices (i.e., prosthetics). This can be approached by directly measuring brain activity at various spatial and temporal resolutions or simulating brain activity using theoretical models.^20^

**Figure 1:**
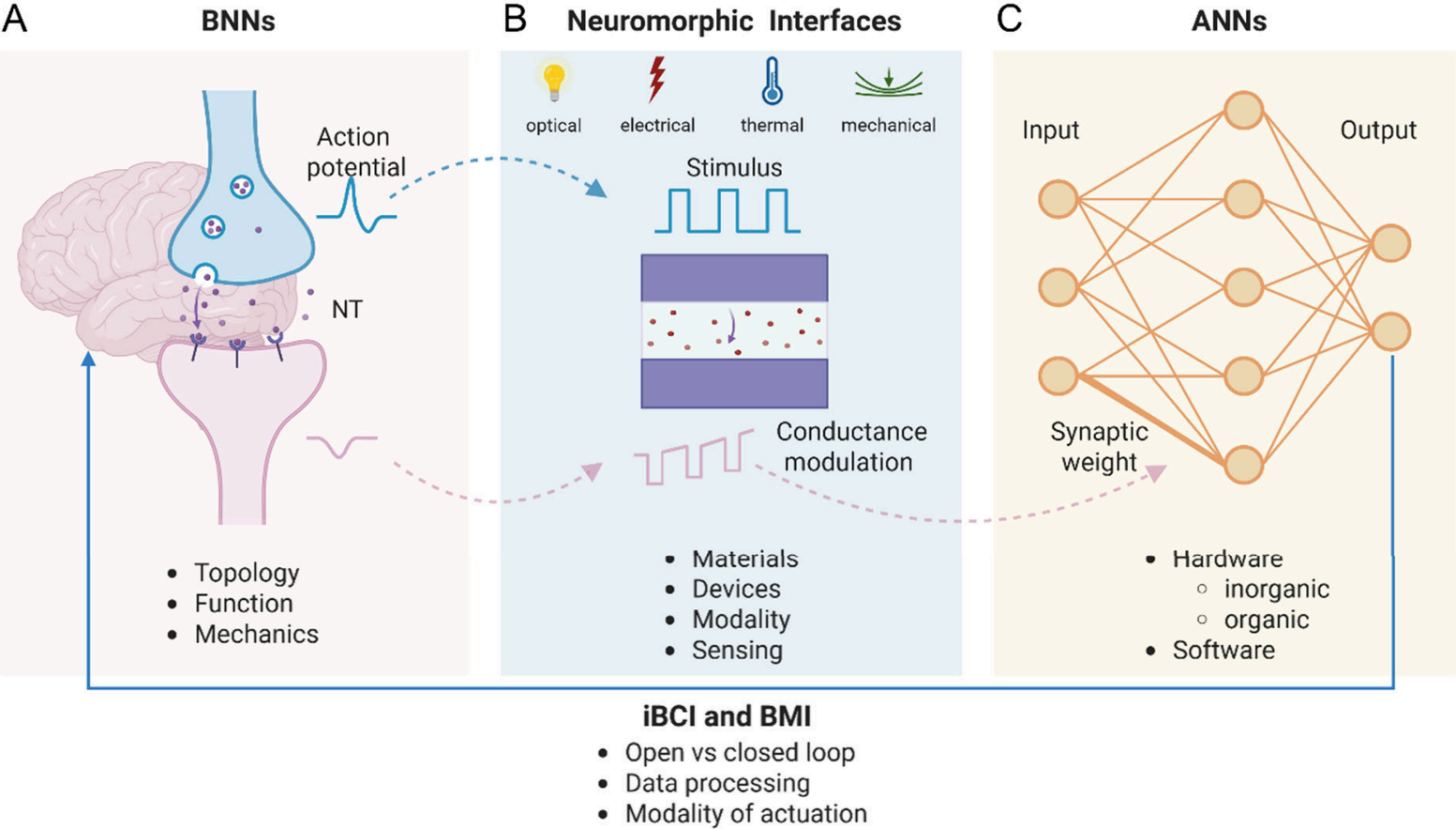
**A. Biological neural networks** have features in their topology, function and mechanics that make them efficient in terms of power consumption and computing capabilities. In particular, the synaptic transmission of information, in which an action potential elicits neurotransmitter release in the synaptic cleft and a postsynaptic potential, is mimicked in neuromorphic devices. **B. Neuromorphic interfaces** have different requirements in terms of neural-inspired behavior. The main feature is that a stimulus (optical, electrical, thermic or mechanical signal) can modulate the conductance of the device like the action potential causes a voltage modulation in the postsynaptic membrane. At the same time, the materials, the design of the device, and the modality adopted should be biocompatible and allow sensing biological signals when in contact with neurons. **C. Artificial neural networks** can be software or hardware and they are constituted of different neuromorphic units in which the connections are represented by synaptic weights, quantifying the strength of the communication. In hardware neural networks, such as crossbar arrays, the synaptic weights depend on the conductance modulation of the neuromorphic devices, while in software applications these values are used in mathematical models. All these architectures can be used to improve the computing capabilities and data processing in both BCIs and BMIs. The output signal from these systems can be used to control an actuation stimulus in the biological environment, allowing for an advantage over open-loop systems in terms of accuracy. Created in BioRender. Santoro, F. (2024) https://BioRender.com/l94w533.

Traditional methods involve capturing brain activity in real-time, significantly advancing our understanding of brain function. However, BMIs are platforms that require chronic implantation, which raises challenges in terms of long-term stability due to implant-failure risks, immune responses and high cost. Additionally, BMIs might raise ethical concerns due to the use of human or animal models for experiments. However, the rise of neural data, along with advances in modeling and machine learning, is supporting neuroscience research to create comprehensive digital models of the brain (i.e., digital twins^21,22^ ), possibly reducing experimental costs and mitigating ethical concerns.

In fact, these models can support neuroscience research as they can simulate brain functions at different scales from a single cell genome to complete brain regions such as sensory, motor, temporal, or visual cortices, suggesting that this field could be able to integrate different systems to progressively simulate the entire central nervous system.

### 1.2 Neural Recordings and Stimulation

Neural interfaces can either record (“read”) or stimulate (“write” to) neural activity. Neural recordings can be obtained through various invasive (e.g., BMIs such as ECoG) or noninvasive (e.g., BCIs such as EEG) methods. In some cases, neural interfaces influence neural activity indirectly by applying external stimuli (i.e., visual inputs). Common BCIs based on neural recordings include spellers, which use visual stimuli to generate brain activity patterns that are then converted into letters on a user interface.^23^ On the other hand, brain stimulation directly activates or inhibits specific brain areas through different means of stimulation (i.e., electrical) to modulate neuronal information processing.

Successful examples include, for instance, BMIs such as DBS where electrodes are connected to a pulse generator that delivers controlled electrical stimulation to target brain areas. It is primarily used to treat neurological conditions such as Parkinson’s disease, essential tremor, dystonia, and sometimes psychiatric conditions. However, the procedure is invasive, requiring surgery to implant electrodes in the brain and patients might experience side effects such as speech difficulties and balance problems with additional device maintenance requirements like battery replacements and long-term effect not yet well understood.^24^

Another example is cochlear implants that provide a sense of sound to patients affected by hearing loss. The device typically consists of an external microphone and a receiver that is implanted to convert sound into an electrical signal. In this context, current challenges still address improvement of sound perception as well as high surgical risks due to device failure.

Most BCI and BMI systems currently use only one mode of interfacing, either reading from or writing to the brain. Recently, the integration of both modalities into bidirectional communication systems has become a key goal in the neuroelectronic field, as evidenced by advancements in clinical applications such as closed-loop DBS.^25^ These approaches are expected to become more common in the future, enabling next-generation applications with multimodal interfacing and on-chip computing.

### 1.3 Software vs Hardware Development

In the past decade, software advancements have significantly improved neural interfaces through innovative processing techniques and a deeper understanding of brain structure and functions. Furthermore, enhanced SNR of neural recordings allows for more precise measurement of brain activity. Additionally, advancements in machine learning, particularly deep learning, have enabled the discovery of novel brain features and the creation of complex classification models to handle highly dimensional input data. Open-source software tools have been crucial in making these advancements accessible to researchers and end-users. Tools like EEGLAB, OpenViBE, BCI2000, and MNE^26^ are widely used in research laboratories and neurotechnology companies worldwide.

In contrast, hardware development has progressed more slowly due to high costs and the time required for prototype development.^27^ For example, the Utah array, introduced 25 years ago, enabled the recording of large populations of neurons with sufficient SNR for developing precise BMIs, and it remains the gold standard for invasive brain recordings.^28^ Most BCIs still rely on EEG, a technology introduced nearly a century ago. However, promising developments include a shift from wet to dry electrodes, which are cheaper, quicker to set up, and provide comparable measurements. Significant advances have also been made in miniaturizing electronic components, leading to more efficient and cost-effective processing boards necessary for advanced neural interfaces. Like open-source software, open hardware initiatives have driven innovation in electrical circuits for neural interfaces with relevant examples such as OpenBCI and various research laboratories demonstrating how to build low-cost BCIs using consumer-grade electrical components.^29^

### 1.4 Coupling Neuromorphic Devices with Neural Interfaces

Inspired by BNNs, ANNs consist of billions of simulated neurons interconnected to perform complex tasks, often surpassing human performance.^30^ ANNs have revolutionized information technology, leading to more optimized and autonomous artificial intelligence systems. However, because ANNs operate on traditional Von Neumann machines, they require significant power, even on optimized hardware like GPUs.^31^ To address this issue and better mimic the energy efficiency of BNNs, SNNs were developed, where computations occur asynchronously without synchronization by an external clock. SNNs can run on neuromorphic processors that facilitate analog-like asynchronous communication, with several commercial solutions now available, such as Intel’s Loihi, IBM’s TrueNorth, and SpiNNaker.^32^ Considerable efforts have been made to develop novel biomimetic devices that replicate the biological mechanisms contributing to the brain’s efficient neuronal communication, exploring new inorganic materials and creating hardware-based neuromorphic devices that function like biological neurons, emulate synaptic plasticity, and demonstrate learning capabilities.A major focus of neuromorphic engineering is to implement the functional principles of biological synapses and neurons into novel devices that allow adaptive signal processing and learning with much lower energy demands compared to traditional computers.

One relevant challenge for neural implants is that signal transfer between cells and devices, and encoded information content can fluctuate significantly over time. This limits their long-term usability for recording and/or stimulation. Movements of the neural device within neural tissue or its encapsulation by scar tissue can strongly affect which neural signals are recorded or reduce the efficacy of neural circuit stimulation. This is particularly challenging for the development of bidirectional closed-loop applications, where specific neural activity patterns should trigger corresponding stimulation patterns; for example, recognizing and disrupting pathological activity patterns to treat focal epilepsy. The edge computing and learning capabilities of neuromorphic devices could be instrumental in continuously adjusting their input/output behavior to identify optimal stimulation patterns and reliably prevent seizure formation over long time scales.

Aside from continuously adjusting to changes in neural signals, flexible materials are also key for long-term functionality because the mismatch between rigid neural devices and soft brain tissue can trigger a FBR, leading to device encapsulation or rejection. Low-power, organic, and flexible neuromorphic devices are therefore ideally suited to promote long-term closed-loop applications to treat neural disorders. Other important aspects to consider in the design of neuromorphic implants are the signaling modalities and spatiotemporal resolution that can be exploited. While many existing devices focus on electrical signals as a readout, neurotransmission is highly multimodal and consists of electrical action potentials as well as a large array of neurochemical signals that shape synaptic transmission and signal integration in single neurons. Neural signals also operate on a broad range of time scales, with action potentials and synaptic events occurring in the millisecond range, alongside minute- or hour-long fluctuations in extracellular neuromodulators and changes in synaptic plasticity.^33^

Lastly, the strength and spatial specificity of neurotransmission can vary by several orders of magnitude, from transient millimolar release within the cleft of chemical synapses to diffuse picomolar concentrations during long-lasting volume secretion.^34^ To effectively interact with such complex and dynamic signaling environments, neuromorphic devices must be designed to accommodate the diversity of neurochemical communication. Key engineering parameters, such as device sensitivity, spatial resolution, and response time, are essential to process both fast electrical signals and slower neurochemical fluctuations at biologically relevant scales. For instance, detecting gradual increases in extracellular glutamate levels could enhance seizure prediction in epilepsy, while monitoring GABA diffusion might support timely intervention to prevent seizure onset.^35^ By integrating electrical and neurochemical sensing and stimulation, future neuromorphic platforms could enable more biologically faithful interactions with neural circuits, leading to more robust learning mechanisms, adaptive stimulation, and advanced therapeutic strategies.

Extending from this need for dynamic, responsive interfaces, we introduce the concept of NNNs, systems that merge the adaptability and efficiency of the human brain with the computational power of neuromorphic architectures. These hybrid platforms aim to establish a two-way interface between living neuronal networks and artificial systems, enabling real-time information processing and feedback. To realize this vision, several challenges must be addressed, including minimizing energy consumption, ensuring stable long-term operation, and managing high-throughput data in real-time. Advances in neuromorphic engineering, particularly in the design of spiking neural networks, analog computing elements, and on-device learning algorithms, will be crucial to support the seamless integration of biological and artificial components. Ultimately, NNNs hold promise not only for next-generation neural interfaces but also for redefining how we interact with, repair, and augment the nervous system in both clinical and research settings.

### 1.5 Integrating Neuromorphic Intelligence into Neural Interfaces

In this review, we aim to discuss how to bridge the gap between conventional neural interfacing technologies and the rapidly evolving field of neuromorphic engineering by exploring how emerging materials and device architectures can support seamless, bidirectional communication between biological and artificial neuronal systems. The next-generation neurotechnologies might go beyond passive sensing and stimulation, toward platforms that can emulate the adaptive, low-power, and real-time processing capabilities of the human brain. Neuromorphic systems, designed to mimic the computational principles of biological neural networks, offer a transformative approach to interfacing with the nervous system, enabling local processing, synaptic-like plasticity, and closed-loop feedback. These features are particularly critical for overcoming long-standing challenges in neural interfaces, such as signal degradation, FBR, and the lack of intelligent responsiveness to dynamic biological environments.

This review begins by elaborating on traditional neural interface strategies with neuromorphic paradigms in terms of recording fidelity, stimulation selectivity, and computational efficiency. We then discuss the biological foundations of neural communication, including synaptic transmission, plasticity, and neuromodulation, and discuss how these mechanisms inspire the design of neuromorphic platforms. The following sections highlight recent advances in materials for both neural and neuromorphic interfaces, emphasizing the importance of flexible, biocompatible, and multimodal systems capable of integrating electrical, chemical, and optical signaling. We further examine device architectures such as organic transistors, memristors, crossbar arrays, as well as SNNs, that collectively enable intelligent biosignal decoding and adaptive stimulation.

Looking ahead, the convergence of neuromorphic electronics and biointerfaces is expected to catalyze the emergence of NNNs where living neurons and neuromorphic devices operate in synergy. These systems hold promises for personalized neurotherapies, real-time seizure prediction and suppression, closed-loop neuroprosthetics, and brain-inspired computing. In the final sections, we discuss the current limitations and ethical implications of such technologies and offer a forward-looking perspective on how advances in material science, device miniaturization, and embedded AI will shape the future of brain–machine integration and redefine the boundaries of human–machine interaction.

## 2 Biological Neuronal Networks and Information Processing

### 2.1 Biological Synapses and Plasticity

The human brain consists of more than 100 billion neurons, each connected to thousands of others through chemical synapses.^36^ Neural computations occur at various scales, from plastic changes in synaptic transmission to adaptive signal integration within individual neurons and larger biological networks. Individual chemical synapses can be considered the most basic computational units in the brain.^37^ Neurons integrate myriad synaptic inputs to generate an all-or-nothing action potential. Synapses contribute to single-neuron computations by modifying their connection strength, a process known as synaptic plasticity. While plasticity often involves strengthening synaptic connections related to the frequency of transmitted action potentials, various implementations exist throughout the brain, such as the targeted modulation of synaptic plasticity through the corelease of neuromodulators like dopamine. Thus, synaptic plasticity is a dynamic process that enables synapses to function as memory units, providing BNNs with an inherent capacity for learning and memory.^38^

Most neurons consist of a cell body (the soma), dendritic branches that receive most synaptic inputs, and an axon that forms synaptic contacts to transmit action potential signals. Many synapses create a gap between neurons, where signals are transmitted chemically via neurotransmitter release. In contrast, electrical synapses consist of gap junctions between neurons that allow signals to be transmitted directly by ion drift.^39^ In chemical synapses, information is transmitted between the axon of the presynaptic neuron and the dendrites of the postsynaptic neuron by transforming electrical signals (an action potential) into the release of chemical molecules (neurotransmitters), which are then transformed back into action potentials.^40^

Unlike chemical transmission, which occurs from a transmitting presynapse to a receiving postsynapse, electrical synapses allow for bidirectional flow of signals between neurons.^41^ Synaptic transmission is the major means of communication between neurons.^42^ It begins with the depolarization of the presynapse by an incoming action potential, leading to the opening of voltage-gated calcium (Ca^2+^) ion channels. This increase in Ca^2+^ triggers the release of neurotransmitters, which diffuse through the synaptic gap and bind to corresponding receptors on the postsynapse, modulating the activity of the receiving neuron^43^ (Figure 2A-B).

**Figure 2:**
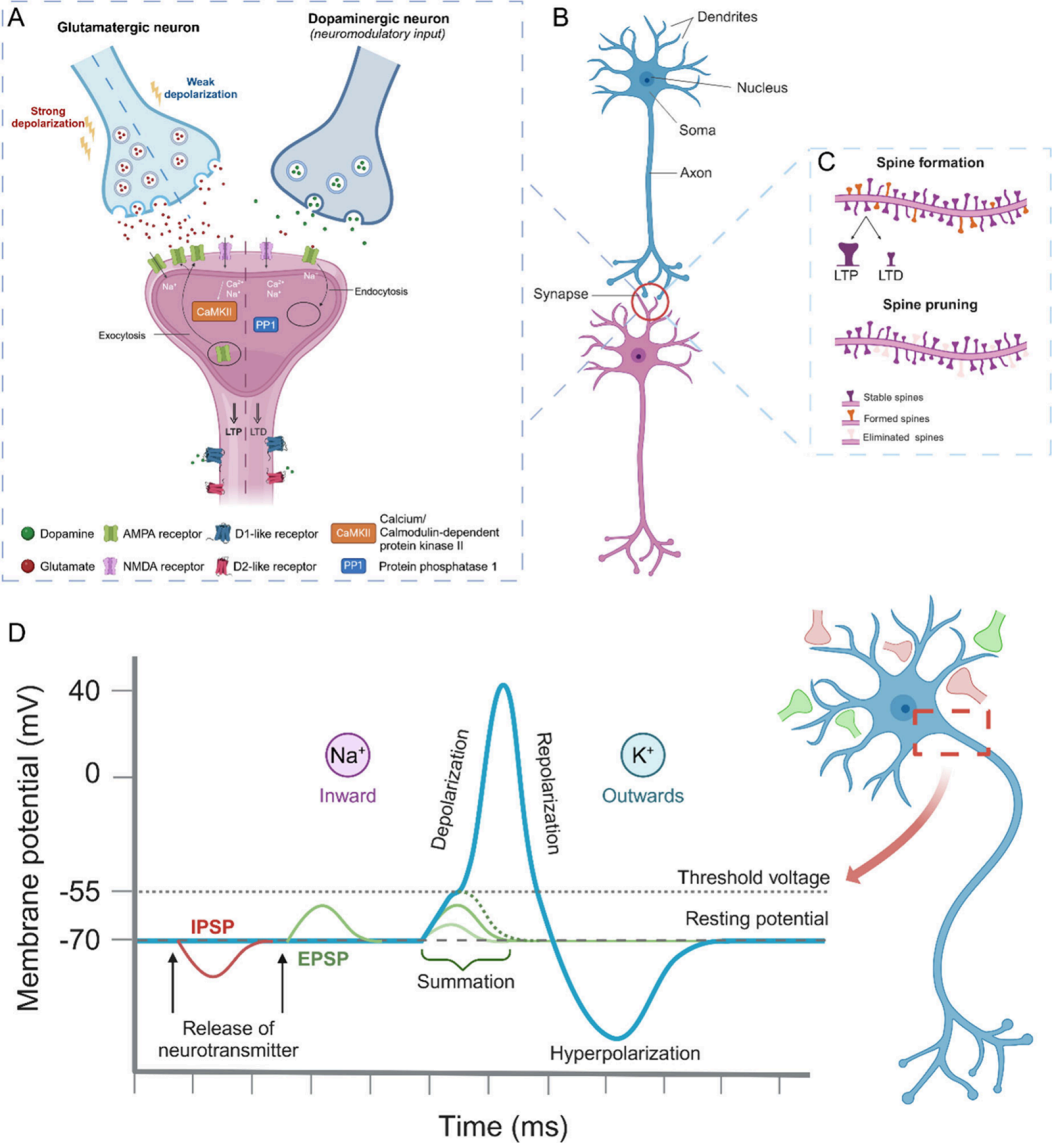
**A. Synaptic plasticity**. Presynaptic depolarization opens voltage-gated Ca^2+^ ion channels, increasing Ca^2+^ levels which triggers glutamate release into the synaptic cleft. Glutamate binds to postsynaptic receptors (AMPA, NMDA), leading to excitatory synaptic transmission. Strong activation causes LTP, via different kinase pathways. Weak activation triggers LTD through phosphatase pathways that reduce receptor density. Dopamine further modulates synaptic plasticity by acting on D1-like receptors to facilitate plasticity or D2-like receptors to reduce plasticity. **B. Synaptic transmission**. Schematic of two glutamatergic neurons interacting via a chemical synapse. **C. Structural plasticity**. Illustration of dynamic formation and elimination of dendritic spines to refine neural circuit function. Structural changes support synaptic plasticity, with LTP causing spine head enlargement, and LTD spine head shrinkage. **D. Action potential**. Graded postsynaptic potentials are typically triggered by synaptic inputs. Depending on the type of neurotransmitter, the resulting postsynaptic potentials can be depolarizing or hyperpolarizing. Synaptic inputs are usually received at the dendrite and integrated at the cell body, where bursts of incoming signals are also integrated over short time intervals. An action potential is triggered when the membrane potential reaches a certain threshold, which is typically around −55 mV. The generation of action potential consists of three phases. 1) Depolarization phase: reaching the threshold causes an opening of voltage-gated sodium channels and sodium influx which further depolarizes the cell. 2) Repolarization phase: inactivation of sodium channels and potassium efflux through potassium channels, bringing the potential back toward resting membrane voltage. 3) Hyperpolarization phase: the membrane potential briefly becomes more negative than the resting level due to prolonged potassium permeability, before it returns to the resting state. Due to the inactivation of sodium channels, no action potentials can occur during the repolarization phase (also known as the absolute refractory period), while the likelihood of triggering an action potential is reduced in the hyperpolarization phase (relative refractory period). Created in BioRender. Santoro, F. (2024) https://BioRender.com/s67f452.

An important mechanism that significantly impacts communication between neurons at the synaptic level is *synaptic plasticity*, enabling modulation of receptor density and size at the postsynapse, as well as increasing neurotransmitter release in response to incoming action potentials. This is a key mechanism underlying learning and memory processes. Early on, it was suggested that synapses strengthen their connections if the presynaptic neuron consistently participates in firing the postsynaptic neuron, a concept known as the *Hebbian rule*.^44^ A specific form of the Hebbian rule, called STDP, was later demonstrated in cultured hippocampal neurons.^45^ In this process, the connection strength between neurons increases if presynaptic spikes precede postsynaptic spikes within a specific time window (20 ms), leading to LTP of synaptic contacts. Conversely, if postsynaptic spiking occurs before presynaptic activation, synaptic strength decreases, resulting in Ltd.

Both LTP and LTD forms are highly dependent on the activation of NMDARs, which play a critical role in hippocampal synaptic plasticity.^46,47^ NMDARs regulate synaptic, dendritic, and neuronal plasticity through a calcium-dependent signaling cascade that alters intracellular protein synthesis, such as increasing the density of postsynaptic receptors.^48^ NMDARs become active when they bind the excitatory neurotransmitter glutamate while the postsynaptic membrane is already depolarized, acting as a coincidence detector for ongoing depolarization and incoming synaptic signals. The combination of depolarization and neurotransmitter signaling leads to the removal of an intracellular magnesium (Mg^2+^) ion from the NMDA receptor pore, allowing for subsequent Ca^2+^ influx that activates downstream signaling cascades.^49^ Ca^2+^ is pivotal in determining whether a synapse undergoes LTD or LTP.^50^ Large increases in intracellular Ca^2+^ activate kinases, inducing LTP, while moderate Ca^2+^ levels activate phosphatases, leading to LTD.^50,51^

Lastly, synaptic plasticity can be divided into several phases based on the cellular mechanisms involved. *Short-term plasticity* lasts approximately 60 min and is mainly mediated by NMDARs.^52,53^ Between 60 and 120 min, mGluRs, kinases, and phosphatases maintain changes in synaptic plasticity, a process called early long-term synaptic plasticity.^50,54^ The later phase, lasting more than 4 h and termed late long-term synaptic plasticity, requires the expression of immediate early genes and protein synthesis.^55^ These protein synthesis changes induce permanent structural and functional alterations in the synaptic connection, enabling biological synapses to encode novel experiences and lifelong memories.^56,57^

### 2.2 Signaling and Neuromodulation

Neuronal interactions occur mainly through key neurotransmitters, which generate EPSPs and IPSPs. **Glutamate**, the primary excitatory neurotransmitter, acts through ionotropic receptors (like AMPA, NMDA, and kainate) and metabotropic receptors (mGluRs). NMDA receptors, which are permeable to Na^+^, K^+^, and Ca^2+^, are particularly important in synaptic plasticity, especially in the hippocampus.^46,47^ **GABA** serves as the main inhibitory neurotransmitter, binding to ionotropic (GABA_A_) and metabotropic (GABA_B_) receptors. GABA binding induces hyperpolarization, making action potential generation less likely, while NMDA receptors play a crucial role in synaptic plasticity via Ca^2+^ influx.^58^

Furthermore, the process by which neurochemical agents (i.e., neurotransmitters and other signaling molecules) adjust the activity of neurons and synapses, influencing their response to inputs is defined as neuromodulation. Unlike direct synaptic transmission, which rapidly activates neurons, neuromodulation typically operates over longer time scales and affects multiple neurons simultaneously. It can enhance or suppress neuronal activity, thus modulating functions such as mood, attention, and pain perception. Common neuromodulators include serotonin, dopamine, and norepinephrine, each playing distinct roles in regulating various brain functions. Aside from modulating synaptic transmission and plasticity, some of these neuromodulators are also involved in regulating brain states, such as attention, arousal or sleep, which strongly affect information processing within a neural network.^59^

Beyond their classical synaptic actions, most neuromodulators, including serotonin, also communicate through a mechanism known as volume transmission. In contrast to local synaptic interactions, this form of transmission refers to the nonsynaptic, diffuse release of neurotransmitters, which allows their actions to occur at relatively distant sites from the point of release.^60^ Volume transmission has been suggested as a significant mode of communication for noradrenaline, acetylcholine, and serotonin to induce strong effects on network function over longer time scales.^61^ For example, these neuromodulators can regulate their own presynaptic release^62,63^ and they might also modulate the release of one another and other neurotransmitters.^64–66^ Volume transmission is therefore of particular importance for the broader and long-lasting effects of neuromodulators on neural network function.

### 2.3 Electrical Communication

Graded potentials are continuous changes in the membrane potential of neurons that vary in amplitude depending on the strength of incoming synaptic signals. Several types of graded potentials exist in the nervous system, including synaptic, receptor, and electrotonic potentials.^67^ Graded potentials are typically triggered by external stimuli, such as sensory stimuli in the case of receptor potentials, or by neurotransmitter binding in the case of postsynaptic potentials. Depending on the type of released neurotransmitter and its corresponding postsynaptic receptors, the resulting potentials can be either EPSPs or IPSPs.

These postsynaptic potentials are primarily created by ionotropic receptors, which open upon binding a specific neurotransmitter, allowing charged ions, such as sodium (Na^+^) or chloride (Cl^–^), to move across the neuronal membrane and change the local electrochemical potential.

The integration of all incoming synaptic signals determines the overall membrane potential at the neuron’s soma, influencing the likelihood of generating an action potential. Since each neuron receives synaptic input from thousands of other neurons, the generation of the action potential depends on the summation of all the EPSPs and IPSPs (Figure 2D). Spatial summation occurs when multiple synaptic inputs are received simultaneously from different locations, increasing the likelihood of generating an action potential if more EPSPs are received. Temporal summation occurs when incoming EPSPs and IPSPs are integrated within specific time intervals, with rapid bursts of EPSPs having a higher chance of triggering an action potential.^68^

In contrast to postsynaptic potentials, an action potential is an all-or-none phenomenon, meaning it is either fully generated or does not occur at all. Once the membrane potential reaches a certain threshold, typically around −55 mV,^69^ the neuron will fire an action potential. Postsynaptic potentials, particularly EPSPs, play a crucial role in driving the membrane potential toward this threshold. In contrast to postsynaptic potentials, which generally have small amplitudes varying between 1 and 50 mV, the amplitude of an action potential is larger (∼100 mV) and is independent of the stimulus strength.^67^ An action potential consists of three phases: depolarization, repolarization, and hyperpolarization.

Depolarization is initiated when the membrane potential reaches the firing threshold voltage, causing voltage-gated sodium channels to open and Na^+^ ions to flow into the cell.^70^ The inward flow of Na^+^ produces a rapid rise in membrane potential, temporarily reversing its polarity. Depolarization in mature neurons lasts approximately 1 ms, during which the sodium channels become inactivated.^71^

During the repolarization phase, voltage-gated potassium (K^+^) channels open, allowing K^+^ ions to flow out of the neuron, which drives the membrane potential back toward its resting membrane voltage. The outward flow of K^+^ continues briefly beyond the resting potential, resulting in temporary hyperpolarization, where the membrane potential becomes more negative than usual. During this phase, the neuron is less likely to fire another action potential. Once the action potential is generated, it moves along the axon until it reaches a presynaptic axonal terminal. Here, voltage-gated Ca^2+^ channels cause an influx of Ca^2+^ ions into the cell, triggering the release of neurotransmitters into the synaptic cleft, where they bind to receptors on the postsynaptic neuron. The duration of the action potential is relatively short (2–4 ms) compared to the duration of graded potentials, which can last for seconds.^67^

Action potentials are followed by a brief refractory period, which can be divided into two phases: the absolute refractory period, during which no further action potentials can be generated, and the relative refractory period, during which particularly strong stimuli are required to trigger additional action potentials.^72^ The refractory period is crucial to ensure that action potentials remain discrete, nonoverlapping signals and maintains the unidirectional flow of action potentials along the axon. It also serves as a fundamental constraint on how neurons transmit information and imposes an upper limit on possible firing rates. While specialized interneurons can approach the theoretical limit of around 500 Hz, most neurons operate at lower firing rates between 1 and 100 Hz, depending on their mode of action. At rest, the firing rate of most excitatory cells is relatively low but can strongly increase during active processing.

Of particular importance are short, high-frequency bursts of action potentials in response to particularly salient events. Such bursts have a strong impact on synaptic plasticity and signal transmission and are linked to sensory perception and the learning and memory of important events. In contrast, tonic firing refers to regular firing patterns at a relatively constant rate over time. Unlike bursts, tonic firing is used to maintain a continuous background signal and is often utilized for homeostatic functions, such as breathing or heart rate, or to provide a constant level of neuromodulation, for example, during states of heightened attention.

### 2.4 Structural Plasticity and Mechanics

Structural plasticity is the ability of living neural circuits to alter their physical structures, either during brain development or as a mechanism to support functional plasticity, such as the strengthening or pruning of synaptic connections between individual neurons.^73^ It involves changes in the number and strength of synapses, including rearrangements at pre-existing synapses, the formation of new synapses (synaptogenesis), and the elimination of existing ones (synaptic pruning)^74^ (Figure 2C). Structural plasticity is closely linked to the learning process: learning leads to changes in synaptic transmission that must be stabilized and consolidated through structural changes to enable long-term memory formation or lasting changes in neural network function.^75,76^ The creation of stable, persistent long-term memory therefore requires molecular changes, including changes in gene expression and protein synthesis, which are closely tied to structural changes in synaptic morphology. These structural changes, occurring over extended time periods from hours to days, are essential for stabilizing and maintaining synaptic modifications, especially at the postsynapse or dendritic spines where postsynapses are commonly located.

The induction of synaptic plasticity is associated with changes in the number and morphology of dendritic spines, increasing synaptic stability that make functional connections more persistent. Early electron microscopy studies have shown that the induction of synaptic plasticity can influence the size and shape of dendritic spines.^77–79^ For example, an increase in synaptic strength is correlated with an enlargement of the spine head which depends on actin polymerization and NMDA receptor activity.^80^ These morphological changes modulate synaptic transmission by increasing the density of neurotransmitter receptors and influencing the calcium influx into the dendrite. In addition, an increase in spine number can enhance synaptic transmission strength, as it allows for the formation of more connections with presynaptic neurons.

Structural changes in the postsynapse are often initiated by elevations in intracellular calcium due to incoming synaptic signals, triggering subsequent activation of second messenger signaling pathways. One crucial pathway for regulating activity-mediated stabilization of dendritic spines involves the activation of intracellular kinases. Furthermore, local protein synthesis close to the synapse is also essential for maintaining LTP and promoting spine enlargement.^81,82^

Electrophysiological studies indicate that the rapid formation and persistence of cytoskeletal F-actin in spines after LTP induction triggers cytoskeletal reorganizations that result in the formation of new synaptic structures.^83,84^ Adhesion molecules also play a role in stabilizing neuron connectivity^85^ and regulate dendritic spine morphology and function by influencing synaptic size and strength.^86,87^ These cytoskeletal and adhesion remodeling processes after learning lead to the formation of new synaptic connections.

Overall, structural plasticity after learning is supported by the coordinated interaction of multiple molecular processes. For example, NMDA receptor-initiated actin cytoskeleton dynamics regulate the insertion of glutamate receptors into synapses, which are further stabilized by upregulation of adhesion molecules that maintain connectivity between neurons.^88^ While early electrophysiological studies primarily focused on spine growth and synapse formation in response to neuronal activation, recent findings show that learning can also result in the rapid rewiring of existing synapses through spine formation and elimination.^89^ Structural plasticity also facilitates neural recovery after injury by remodeling dendritic spines and axons. In conclusion, structural plasticity is a dynamic process that allows the brain to reorganize its neuronal networks based on experience, playing a critical role in learning, memory persistence, and recovery from injury.

### 2.5 Photon Sensing

Detecting optical signals allows animals to locate distant prey, and avoid approaching predators, as well as coordinating biological processes to the day/night cycle of the earth. In the most simple form, this can be a light sensitive ion channel, as in green algae (see also section 3.4.3).^90^ In more advanced organisms, the transduction of light into a neuronal signal has taken a far more complicated path in order to allow perception across many orders of magnitude of signal. Vision in the mammalian eye is typically described as an example of exquisite performance arising from the most counterintuitive engineering design. As light enters the eye it is focused onto the retina (Figure 3A), where it first passes *through* various cell layers that will preprocess the visual signal before information is sent to the brain. At the distal end of the tissue, cones (color-sensing) or rods (black/white detectors) contain pigments to absorb incoming photons. In the case of rods, the incoming photon is absorbed by rhodopsin (Figure 3B), inducing an 11-cis to trans isomerization of retinal.^91^ This is the first in a long line of signal transduction steps, each of which allows tuning and amplitude modulation. When the opsin absorbs light energy, the induced retinal isomerization causes a conformational change in the opsin structure. Rather than directly gating a membrane channel, this isomerization activates the enzyme transducin. Transducin amplifies the signal by activating photoreceptor diesterase that hydrolyses cGMP. Sodium channels that require cGMP to stay open therefore close. Closing sodium channels shifts the polarization across the photoreceptor membrane away from the sodium equilibrium potential. This converts the chemical signal into an electrical signal which propagates to the proximal end of the photoreceptor cell. When the synapses at the proximal end of the photoreceptor experience a more negative potential, the tonic release of the neurotransmitter glutamate is reduced^92^ (Figure 3C). This reduces the chemical signaling from photoreceptor cells to the overlying layers of the retina such as bipolar cells and amicrine cells. Dependent on which receptor each bipolar or amicrine cell expresses, the reduced glutamate will either depolarize or hyperpolarize the cell. Thus, the signal is converted again from chemical to electrical, with the opportunity to integrate signaling horizontally in the retinal layers.^93^ The photoreceptor information is condensed at this stage by a factor of approximately 3. The electrical signaling in these layers is not the spiking information coding of action potentials that other neural tissues utilize. These signals remain graded electrical responses without spiking. Only after passing another chemical synapse from bipolar cells to retinal ganglion cells is a spiking neuron modulated by the light signal. From this point the optical information follows the more common neural coding of electrical action potentials transmitted to downstream cells by chemical synapses, as discussed in section 2.1.

**Figure 3:**
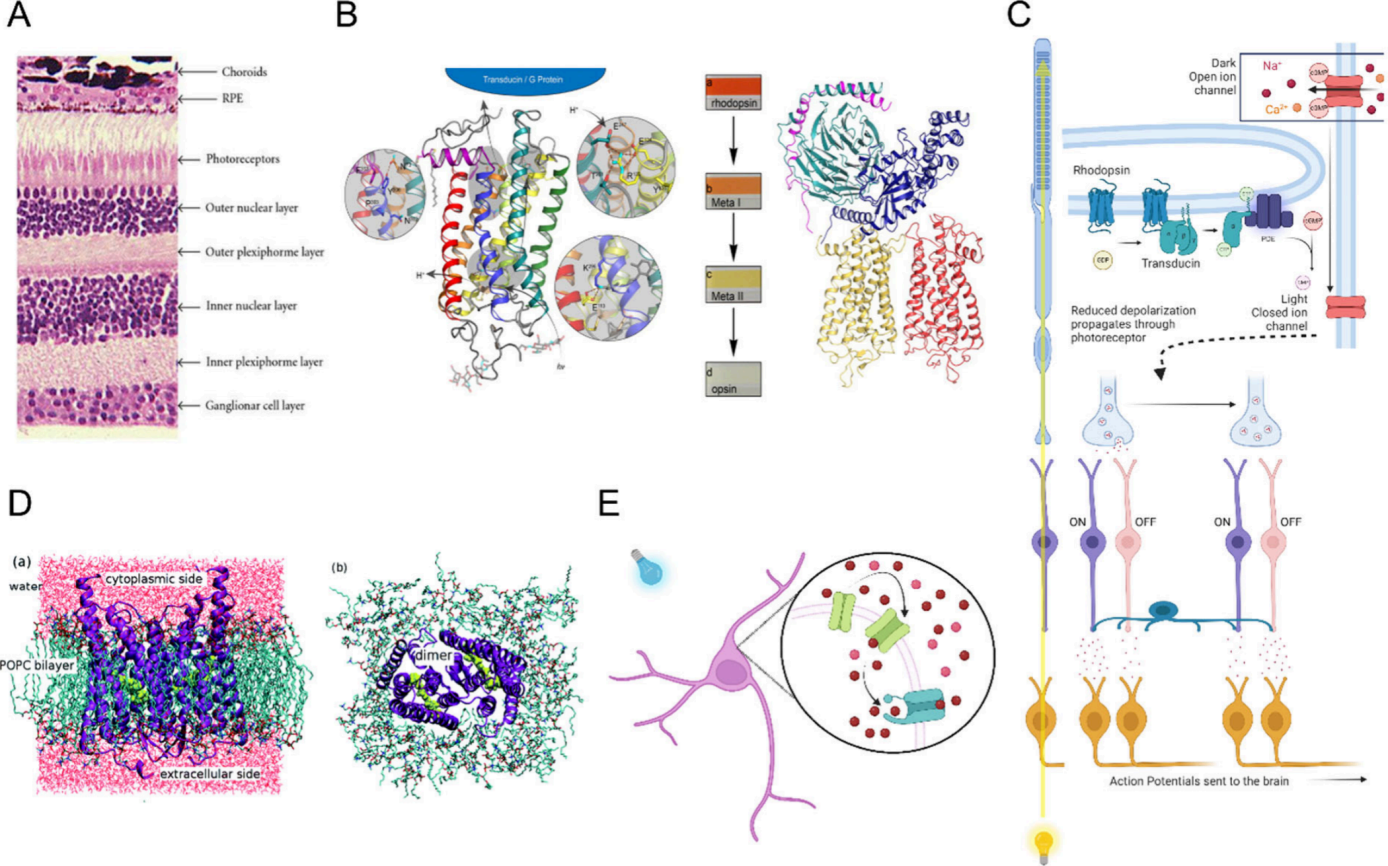
**Photon sensing. A.** Lateral view of a stained retina (adapted from reference 121) showing the layered structure of cells, with photoreceptors at the top (back of the eye). Copyright 2010, Hindawi Publishing Corporation. Distributed under a Creative Commons CC BY License (CC BY 3.0). **B.** The protein structure of mammalian rhodopsin (adapted from reference 122), the photoreceptor pigment. Though the trans membrane domain arrangement (yellow and red) is similar to that of Channelrhodopsin (see panel D) the retinal is a different isomer and no pore is formed by the protein. Copyright 2012, American Society for Biochemistry and Molecular Biology. Distributed under a Creative Commons CC BY License (CC BY 4.0). **C.** Schematic representation of the retina. Light enters from the bottom and traverses all layers of cells, as seen on the left. Light absorbed by Rhodopsin initiates a chemical pathway that closes cGMP gated sodium and calcium channels. This reduces the depolarization of the photoreceptor cell so that less glutamate is released onto bipolar cells. ON bipolar cells depolarize in the presence of glutamate, and OFF bipolar cells hyperpolarize in the presence of glutamate. The graded response of the bipolar cells modulates their own release of glutamate onto retinal ganglion cells. The synapse from the bipolar cells to the retinal ganglion cells is further modulated across pixels by the amacrine cells. Glutamate at the retinal ganglion synapse alters the firing rate of ganglion cell action potentials that are sent to the brain. Created in BioRender. Santoro, F. (2024) https://BioRender.com/s67f452. **Optogenetics D.** The structure of a Channelrhodopsin as seen from the side and from the top (adapted from reference 123). In contrast to the photoreceptor rhodopsin, there is no large enzymatic attachment and light causes a pore to open directly in the Channelrhodopsin. Copyright 2016, Royal Society of Chemistry. Distributed under a Creative Commons CC BY Unported license (CC BY 3.0). **E.** In a neuron expressing channelrhodopsins (green), light opens the channel, causing depolarization; when depolarization reaches the threshold, voltage-gated sodium channels (blue) open to cause an action potential, as shown schematically. Created in BioRender. Santoro, F. (2024) https://BioRender.com/s67f452.

Information processing in the nonspiking cellular layers of the retina amplifies weak signals and distinguishes visual information into categorical types, such as edges, moving objects, etc. This multistep amplification allows humans to perceive light down to a single photon.^94^ The facilitation within the retina also promotes the perception of a single photon if the retina has been primed by recently receiving a single photon stimulation. When the intensity of the incoming light signal increases, a logarithmic response of the cascade assures a high dynamic range (approximately 10 orders of magnitude) of the visual system. Since opsin absorption of photonic energy depends on wavelength, the multistep process further allows color vision by limiting the first step in the cascade to particular wavelengths in the cone cell photoreceptor. Human opsins tune the photon absorption range from about 380–700 nm. The failure of a body to produce opsins will therefore result in color-blindness, without an effect on rod-based vision or overall light sensitivity. Multiplexing wavelength, intensity, and preprocessing class such as movement, allows an information rich signal to be sent from the retinal ganglion cells to the visual cortex of the brain. The system also demonstrates the combination of continuous and spiking information systems to increase information density

## 3 Designing and Engineering Neural Interfaces

### 3.1 General Requirements

Neurotechnologies designed to interface with the nervous system enable the recording, stimulation, and modulation of neural activity from various neural structures (e.g., the brain, retina, spinal cord, and peripheral nerves). These interfaces are employed in both *in vitro* and *in vivo* settings for applications ranging from neuroscience research to clinical therapies, addressing both acute and chronic conditions. To establish stable physical interactions with biological targets, neural interfaces are engineered to match the topological, mechanical, and functional characteristics of nervous tissue. Neural interaction can thus be achieved through different physical modalities, such as electrical, optical, or chemical signals.^124–126^

Interfacing with the nervous system involves physical interaction at multiple levels, from dissociated neurons and organoids in cell culture to the complex, multilayered structures of nervous tissues in neural slices, nerves, or living organisms. Neural interfaces serve as tools that contact neural structures in various forms. For example, penetrating or protruding probes, such as needle-like or thread-like probes, sample internal structures within the intraneural or intracellular space, with glass micropipettes or flexible nanopipettes. Surface or planar probes, such as ECoGs, interact with the surface or outer layer of neuronal targets. Similarly, wrapping probes^127^ (e.g., cuffs) surround the surface of neural structures (e.g., peripheral nerves or the spinal cord), and sieve probes, typically containing perforated electrodes and guidance channels, are designed to support the regeneration of nerves.^128–131^

To provide appropriate mechanical cues for *in vitro* cell cultures and improve biocompatibility *in vivo*, neural interface design often incorporates soft and flexible materials. This helps minimize neuronal loss and FBRs. Polymeric and, more recently, viscoelastic materials, along with miniaturized designs such as neuron-like or net-like configurations, are used to improve mechanical compliance.^132^ These design strategies reduce cross-sectional footprints, thereby lowering the bending stiffness of the interfaces and improving their conformability to neural targets. As these physical characteristics are directly shaped by the materials employed, careful material selection becomes critical, not only for structural and mechanical integration but also for ensuring long-term stability, signal fidelity, and biological compatibility. The following section examines the materials that enable these functionalities, from insulating substrates to conductive and bioactive components.^125^

### 3.2 Materials for Neural Interfacing

The selection of materials for neural interfaces is critical for ensuring long-term stability, effective signal transduction, and seamless biointegration. Typically, insulating materials form the structural backbone of the interface, while conductive materials enable electrical interconnects and electrode function. Neural tissues are inherently soft and dynamic, making rigid materials such as silicon prone to inducing inflammation and glial scarring.^133,134^ To address these limitations, flexible thin-film polymers like polyimide, SU-8, parylene-C, and PDMS are commonly used due to their chemical inertness, biocompatibility, and mechanical flexibility, with Young’s moduli in the low gigapascal to megapascal range.^135^ These materials also support miniaturized designs, such as mesh-like geometries^136,137^ or submicron-thick layers,^138^ facilitating close and minimally invasive interfacing with neural tissue. To further enhance mechanical compatibility or to achieve transient implantation, researchers have developed biodegradable and viscoelastic materials such as silk fibroin, PCL,^139,140^ PLA, PVA, and alginate-based hydrogels,^141^ some of which mimic the viscoelasticity of the brain itself.^139,141,142^ In addition to biodegradable polymers, transient metals such as magnesium, molybdenum, and zinc are being explored for their ability to serve as temporary conductive elements that safely dissolve *in vivo* after completing their functional role. These materials enable fully resorbable neural interfaces, reducing the need for surgical removal and minimizing long-term tissue disruption.^143^

Conductive materials for neural interfaces must combine high electrical conductivity, mechanical compliance, and biostability. Metals like gold and platinum are frequently used in interconnects, but their limited charge injection capacity often necessitates surface treatments like electrochemical roughening to improve electrode performance. For this purpose, Platinum black is also employed in neural electrodes due to its high surface area and low impedance.^144^ IrOx, with its high electroactivity and porous structure, offers superior ionic-electronic coupling and is often favored for stimulation and recording.^145^ Gallium-based liquid metals (e.g., eGaIn) are also being investigated for soft, stretchable interconnects that maintain high conductivity under deformation.^146^ Carbon-based materials such as graphene and carbon nanotubes offer high conductivity and flexibility, though their mechanical mismatch and potential bioaccumulation pose long-term challenges. In this context, emerging two-dimensional materials like MXenes (e.g., Ti_3_C_2_Tx) offer a promising alternative, combining high conductivity with favorable mechanical properties.^147^ In contrast, organic conductive polymers have emerged as leading candidates due to their biocompatibility and ability to operate in aqueous environments without oxide layer formation.^148^ Among these materials, OMIECs stand out for their mixed ionic/electronic conduction. They are typically composed of a conjugated polymer and a polyelectrolyte, either as blends of distinct polymers, copolymers, or conjugated polyelectrolytes. The most well studied OMIEC in organic neuromorphic devices is PEDOT doped with polystyrenesulfonate (PEDOT:PSS) a member of the polythiophene family. These materials exhibit excellent charge injection capacities and reduced impedance, making them ideal for chronic neural recording and stimulation. To further enhance device longevity and reliability, self-healing conductive polymers have been developed to autonomously repair mechanical damage and preserve electrical continuity. These materials incorporate dynamic bonding mechanisms or embedded healing agents that respond to microcracks or delamination at the electrode-tissue interface, making them particularly valuable for chronic neural implants.^149^

In recent years, organic semiconductors have also been utilized in active components such as OFETs, OECTs, and EGOFETs which will be described in more details in 5.4.2. These devices leverage the inherent volumetric capacitance and ionic mobility of organic materials like PEDOT to amplify weak neural signals and modulate cellular activity with high transconductance at low voltages.^150,151^ Furthermore, photoactive organic materials, such as P3HT and P3HT:PCBM, are being integrated into photovoltaic neural interfaces to achieve wireless, light-driven stimulation. These materials not only offer flexible form factors and biocompatibility but can also support photothermal and photoelectrical effects for localized and minimally invasive neural activation. More recently, NIR-sensitive organic semiconductors like PTB7-Th^152^ and PCPDTBT,^146^ as well as nonfullerene acceptors,^153^ have expanded the applicability of these systems to deeper tissues and subcutaneous stimulation. Additionally, the development of OEPCs and emerging materials like perovskites and QDs further extends the functional landscape of optoelectronic neural interfaces.^154^

Beyond bulk material characteristics, surface modifications play a pivotal role in optimizing long-term performance. Strategies such as applying bioactive coatings (e.g., laminin or hyaluronic acid) promote neuronal adhesion and differentiation, while micro- and nanopatterned textures guide axonal growth and improve integration. Antifouling coatings, including zwitterionic layers, further reduce protein adsorption and inflammation, extending device lifespan. Expanding upon these surface modification strategies, nanozyme-based neural interfaces have been developed to regulate the local oxidative microenvironment. These enzyme-mimetic nanomaterials, capable of eliminating ROS, help mitigate neuroinflammation and oxidative damage at the tissue-electrode interface, thereby enhancing recording stability and biocompatibility. Notably, recent designs have demonstrated significant reductions in impedance and glial activation in vivo.^155^ Ultimately, careful engineering of both structural and functional materials enables the development of high-performance neural interfaces that are not only sensitive and stable but also better aligned with the biological complexity of the nervous system.^156^

### 3.3 Neural Transducers

Building on the material considerations discussed in Section 5.2, the functional integration of neural interfaces relies not only on mechanical and biochemical compatibility, but also on their capacity to transduce biological signals into readable formats. The physical and chemical properties of materials, such as conductivity, charge injection capacity, and biostability, directly influence how effectively these interfaces convert, transmit, and respond to neural activity.

In traditional contexts, a transducer (Figure 4A_i_) is defined as a device that converts artificial or biological signals from one to another. Essentially, it serves as a mediator, translating neural signals, such as electrical or biochemical signals, into a format that computers can process, and vice versa. Bidirectional transducers are particularly valuable for facilitating neural communication by enabling both the recording and modulation of neural activity. While their primary function is signal conversion, integrating processing capabilities further enhances their utility, allowing for more adaptive and efficient neuromodulation.

**Figure 4:**
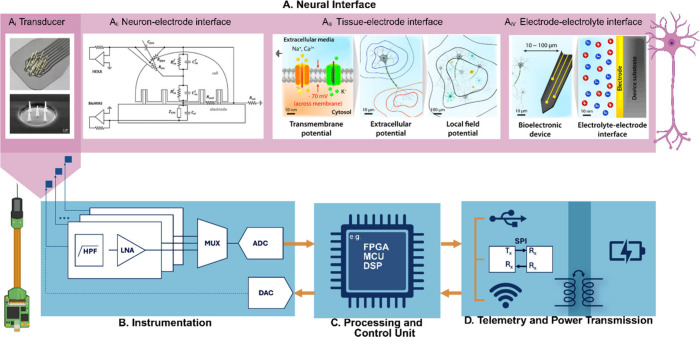
**Conventional signal chain in neural interfaces. A.** When interfacing nervous tissues, a Neural interface comprising a transducer (adapted from reference 171) (A_i_) is used to couple directly with a neural target. The interface between the transducer and the neural target, which can be an isolated neuron or a complex structure, such as a tissue, is governed by the physical characteristics of the neuron-electrode interface (adapted from reference 172) (A_ii_) or tissue-electrode interface (adapted from reference^173^) (A_iii_), dependent on the seal resistance and the distance between the electrode and neural target (current point source), respectively. Transducers can capture the neural activity of individual neurons as transmembrane potentials in the form of intracellular or extracellular action potentials (APs), or the summed activity of groups of neurons in the form of LFPs. Likewise, the quality of the captured activity and modulation processes are then constrained by characteristics of the EDL formed at the electrode–electrolyte interface (adapted from reference^173^) (A_iv_). Copyright 2024, Wiley-VCH GmbH. Distributed under a Creative Commons CC BY License (CC BY 4.0). Copyright 2022, Wiley-VCH GmbH. Distributed under a Creative Commons CC BY NC ND License. Copyright 2019, Royal Society of Chemistry. **B.** Neural activity is then transduced into electrical signals that undergo an instrumentation phase, where the signal is filtered, amplified, multiplexed and digitized for further processing. **C.** This is carried out in a control unit comprising either a FPGA, a microcontroller, or a DSP, when control signals are in turn sent back to the transducer to modulate neural activity. **D.** Finally, digitized data is transmitted and the system is powered for continuous communication. Data is generally transmitted to a computer, where signal postprocessing can be carried out. Created in BioRender. Santoro, F. (2024) https://BioRender.com/s67f452.

A conventional signal chain consists of four primary components (Figure 4): a neural interface, an instrumentation phase, a processing and control unit, and a telemetry unit for data and power transmission. Within this framework, neuromorphic systems are positioned either at the transducer level or within the processing and control unit.

The neural interface (Figure 4A) is the central component of the signal chain. It consists of a biological neural target, such as neurons, tissues, or neural organs like the brain, connected directly to a transducer with both sensing and actuating functions (Figure 4A_i_). Depending on the materials used, the transducer converts various physical signals, electrical, (bio)­chemical, or optical into electronic signals (e.g., voltage or current) for sensing or into physical stimuli for actuation. Transducers are either passive (e.g., MEAs) or active (e.g., memristors, transistors). Passive elements modify or attenuate signals, while active components require external power and can manipulate signals. These elements are mainly in contact with the neural system and physical coupling at different scales between the device and the neuronal cells can limit FBR,^157^ biorecognition and stability in the case of implantable devices. However, the coupling problem also relies on individual cell response, as well as on the plasma membrane’s reaction to the electrochemical and mechanical environment provided by the device surface composition, functionalization and topology.^158^ Here, network development, synaptic formation and maintenance, as well as control over glia cell proliferation should be considered.^159^

Transducers can be one-, two-, or three-terminal devices with electrical contact points that transmit sensory or actuation signals through fixed wiring or multiplexing systems to the next stage: the instrumentation phase (Figure 4B). This stage incorporates key components such as low-noise input amplifiers, filters, ADCs, and DACs, collectively referred to as the analog front end, which amplify, condition, and digitize signals for further processing or reconvert them into analog signals for stimulation.

In recent years, the integration of these elements has shifted from off-chip to on-chip analog front ends to reduce the physical distance between the transducers and the processing stages. These on-chip systems are directly integrated with the transducers and now incorporate preamplification circuitry, multiplexing, data conversion, and even preprocessing and feedback control units, functional blocks that were traditionally performed off-chip during offline signal processing.

Hence processing and control units (Figure 4C) can be integrated with a transducer in the form of application specific integrated circuits or with the implementation of DSPs, FPGAs, or microcontrollers. Various groups have demonstrated such high-level integration for *in vitro* and implantable *in vivo* applications, primarily utilizing CMOS technology. Further details on different instrumentation architectures, front end topologies, and strategies can be found in other reviews.^160–162^

Data transmission, whether analog or digital, can be achieved via wired technologies like USB or SPI, or wirelessly through Bluetooth Low Energy, radiofrequency, or emerging ionic-based communication, which utilizes the high conductivity of biological media.^163–167^ Power can be supplied through wired means, such as external batteries, or wirelessly via inductive coupling or ultrasound-based technologies, which enable both wireless power transfer and communication^168,169^ (Figure 4D). For an in-depth review of wireless power and transmission technologies, readers may consult additional sources.^170^

### 3.4 State of the Art Devices and Architectures for Neuronal Interfaces

The effectiveness of neural transducers depends not only on their material and circuit-level integration, but also on how they are embodied in specific device architectures. Translating the principles of signal transduction into functional systems requires hardware platforms that can interface with complex neural environments across different spatial and temporal scales. As a result, various device-level implementations, ranging from traditional MEAs and transistors to advanced flexible, 3D, and multimodal platforms, have emerged to meet the growing demands of both research and clinical applications. The following section explores these state-of-the-art devices, highlighting their architecture, modes of operation, and the strategies developed to optimize neural coupling and performance in both *in vitro* and *in vivo* settings.

#### 3.4.1 MEAs

These devices consist of a matrix of substrate-integrated electrodes that serve as passive electrical contacts. Their electrochemical behavior, defined by resistive and capacitive properties, is shaped by the electrode material, geometry, and the interface formed with neural tissue. To understand and optimize this interaction, various models have been developed to describe how MEAs engage with neuronal targets. While originally used to characterize transistor interfaces,^174,175^ the point-contact model has been adapted to the neural context to describe the interactions between MEAs and neuronal targets at the electrode-neuron interface^176^ (Figure 4A_ii_). This model focuses on the electrode–neuron interface, where the quality of coupling is primarily determined by the seal resistance, a key parameter representing the tight adhesion between the cell membrane and the electrode surface. Sealing is often enhanced through surface biofunctionalization and coatings, which influence the spacing and adhesion at the cell–electrode junction.^134^

To further enhance cell adhesion and electrical coupling, several research groups have engineered the electrode surface by protein patterning and microfluidic channel to guide cell network arrangement and polarization.^177–179,179,180^ Others implemented 3D nano- and microtopographies, which can modulate the coupling efficiency, also providing tunable contact area and sealing resistance.^181,182^ Hence, topographies, such as cavities, pillars, straws, or mushroom-like structures, recess or protrude from the planar surface of the electrode (Figure 4A_i_), thereby reducing the cleft at the neuron-electrode interface.^172,183–186^

Characterizing this cleft is crucial for understanding the quality of the interface, whereby electrophysiology methods that combine MEAs with invasive patch clamp electrical recordings,^187,188^ as well as with imaging methods, such as scanning or transmission microscopy have been used.^189^ Nonetheless, in interactions between MEAs and nervous tissues, particularly in *in vivo* settings, a tight contact with neural targets is not always achievable due to the presence of FBRs, such as gliosis and microglial insulation.^190^ As a result, MEA-tissue interactions (Figure 4A_iii_) are typically described with a generalized model.^191^ In this model, the electric field generated by neuronal units (current point sources) within the conductive extracellular fluid is a key factor. Assuming the MEA surface is primarily insulated, the voltage or current sensed or delivered by a MEA is inversely proportional to the distance between the electrode and the neuron.^161^

Thus, due to their electrochemical properties and the capability of coupling with single neurons and tissues, MEAs have become essential tools in both research and clinical settings. Offering a high spatiotemporal resolution, MEAs allow for the precise acute and long-term measurement of distinct neural activity. *In vitro*, traditionally planar MEAs on stiff substrates, have been used to characterize neuronal connectivity and signal conduction under physiological and pathological conditions across scales, from single neurons to neuronal population networks. The 2D neuronal cultures and neural slices interface with nano/micro-structured electrodes (Figure 5Ai-ii) or high-density electrodes integrated with CMOS technology.^192,193^ Aiming to enhance the coupling and access the outer and inner volume of 3D neuronal models, such as tissue explants or organoids, innovative MEA designs are emerging.^194,195,195–199^ These include flexible standing 2D substrates, needle-like and biomimetic electrodes protruding several micrometers (Figure 5A_iii_), 2D and 3D architectures containing multisite and multishank electrodes,^200,201,212^ or basket-like devices with mesh structures that optimally interface 3D cell architectures and organoids.^137,202,203^

**Figure 5:**
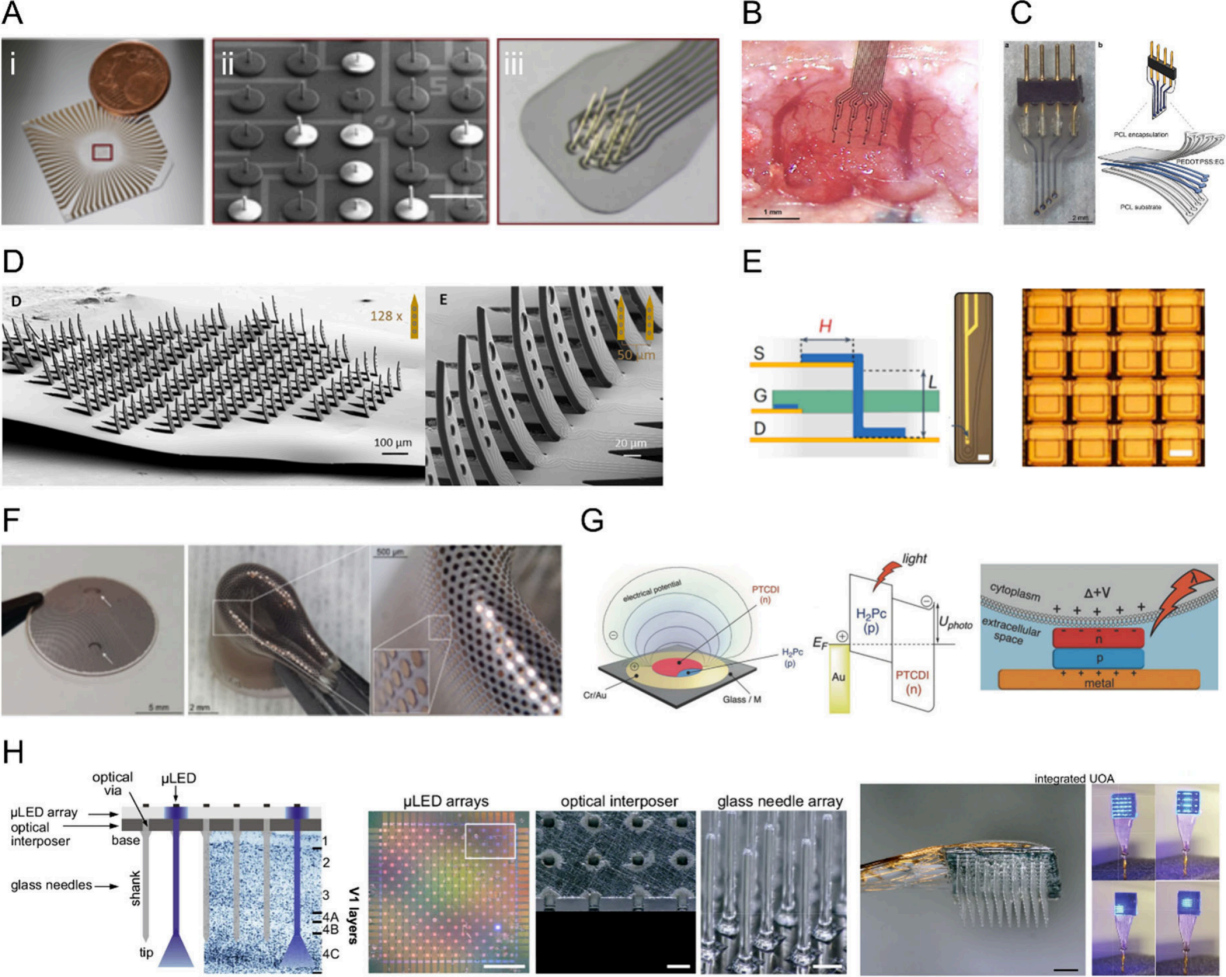
**A.** Needle-like protruding microelectrodes on stiff (A_i_-A_ii_) and flexible substrates (A_iii_) (adapted from reference 171). Copyright 2024, Wiley-VCH GmbH. Distributed under a Creative Commons CC BY License (CC BY 4.0). **B.** Microelectrodes made of reduced graphene oxide (rGO) 16-channel electrodes placed on the cortex of a rat (adapted from reference 210). Copyright 2024, IOP Publishing Ltd. Distributed under a Creative Commons CC BY License (CC BY 4.0). **C.** All-polymeric transient neural probe with four PEDOT:PSS:EG electrodes, highlighting the three layers: PCL encapsulation, PEDOT:PSS:EG electrodes and PCL substrate (adapted from reference 139). Copyright 2021, Elsevier Ltd. Distributed under a Creative Commons CC BY License (CC BY 4.0). **D.** Exemplary 3D kirigami MEA with up to 128 shanks on one flexible probe and an intershank distance as low as 50 μm (adapted from reference 193). Copyright 2025, Wiley-VCH GmbH. Distributed under a Creative Commons CC BY License (CC BY 4.0). **E.** Vertical IGTs (adapted from reference 167). Copyright 2023, Springer Nature. Distributed under a Creative Commons CC BY License (CC BY 4.0). **F.** POLYRETINA is an organic photovoltaic neural interface for artificial vision restoration; being based on polymeric substrate and integrating an organic polymeric photovoltaic layer, it can be folded to facilitate the injection and it can accommodate the natural eye curvature (adapted from reference 247). Copyright 2022, Springer Nature. Distributed under a Creative Commons CC BY License (CC BY 4.0). **G.** Schematic representation of the OEPC, which consists of an organic p-n bilayer patterned on a gold electrode. Upon light illumination, the photogenerated charges induce ionic displacement currents in the surrounding electrolyte, which consequently affect the membrane potential of cells in proximity. If the perturbation is large enough, it can lead to action potential generation (adapted with permission from reference 154). Copyright 2018, Wiley-VCH GmbH. **H.** To bring light to neurons for optogenetic manipulation, various optrode devices have been published (adapted from reference 249). Copyright 2024, Springer Nature. Distributed under a Creative Commons CC BY License (CC BY 4.0).

MEAs are also used for *in vivo* applications that comprise the study of various neural structures in the central and peripheral nervous systems. These applications span fundamental neuroscience and preclinical research, as well as clinical settings. MEAs then function as BMIs or neural prostheses, offering the capability of capturing neural activity in living organisms. The development and application of microelectrodes *in vivo* has been substantial since their inception in the 1950s. Early cortical recordings utilized tungsten microwires^204^ and significant advancement occurred in the 90s with the introduction of silicon micromachined devices, such as the Michigan^205^ and Utah arrays,^28^ and early in the 2000s high-density microwire arrays.^206^ Nonetheless, long-term functionality of MEAs in chronic neural applications is often compromised by biological responses such as gliosis and microglial encapsulation, which interfere with stable electrode–tissue interactions. These responses can lead to reduced SNRs during recording and increased stimulation thresholds. To mitigate these challenges, recent advancements have emphasized the development of more compliant and adaptive interface designs that better accommodate the dynamic environment of neural tissues and reduce chronic inflammation.^207–209^ Among these, flexible μECoG arrays made from organic nanomaterials such as rGO have emerged as promising alternatives to conventional metallic electrodes, demonstrating chronic, high-fidelity recording of both motor and sensory activity.^210^ Building on this platform, similar rGO-based MEAs were recently applied to enable precise deep brain stimulation and mapping in a Parkinsonian rat model, showing their translational potential for targeted neuromodulation.^211^ These graphene-based devices exhibited recording performance comparable to traditional platinum–iridium arrays, while offering advantages in mechanical compliance, biocompatibility, and signal stability (Figure 5B). These efforts include designs and materials that minimize mechanical strain at the interface, enable better conformation to curved or mechanically unstable surfaces, improving the long-term stability of encapsulation layers by incorporating hybrid organic and inorganic (e.g., thin film ceramics) materials (Figure 5C), and reduce the need for surgical implant retrieval by allowing temporary or bioresorbable integration.^139,184,185^

At the same time, advances in device architecture have significantly expanded the functional capabilities of MEAs. These include increased electrode density for high-resolution recording, integration of multisite and multimodal channels, and the development of transparent and optically compatible arrays,.^207^ Additionally, 3D configurations now enable spatially resolved access to neural signals across complex tissue geometries.^165,214,215,218–220,213,214,216,217^ An example is represented by 3D kirigami probes, in which the fabrication process of the devices has been adapted to this requirement^193^ (Figure 5D). Together, these innovations facilitate more comprehensive mapping and modulation of both individual neurons and interconnected neural networks across space and time.^221^

#### 3.4.2 Transistors and Optoelectronic Devices

Transistors are active electronic components that regulate current flow by modulating charge carriers within a semiconductor channel between two terminals (drain and source), controlled by an input signal at a third terminal (gate). As fundamental elements in modern electronics, transistors serve two primary functions: switching and signal amplification. Within neural interfaces, FETs are particularly valuable for their ability to amplify and detect weak neural signals, as well as modulate neural activity. They establish a localized interface between the gate electrode and the neural target, similar to the coupling model described for MEAs, facilitating sensitive transduction.^174^ FET architectures have evolved from planar geometries to nanowire and 3D-penetrating structures, using materials such as silicon^222–224^ and graphene,^225,226^ often integrated with flexible substrates.

These designs have been applied to both *in vitro* systems^174,222–224,227^ and *in vivo* brain recordings, benefiting from FETs’ ability to capture ultraslow neural dynamics, down to 0.025 Hz.^228–230^ These devices offer advantages such as a wider frequency band for neural recording due to low impedances at low frequencies, signal amplification, high sensitivity, miniaturization, scalability, and integration with CMOS electronics. However, their widespread use in neural interfacing has been limited by the reproducibility of complex fabrication processes, high noise at higher frequencies, current drifts over time, and their limited capability to interact with ionic-driven signals.^161,231^

Offering advantages such as enhanced biocompatibility, ease of processing and the capability of mixed charge transport (electronic and ionic functions), organic transistors employ an organic semiconductor material as the transistor channel. Accordingly, the current flow through the channel is modulated by the electric field generated by the gate electrode. Among the most common types, OFETs, EGOTs, divided into EGOFETs, OECTs, and IGTs stand out in interfacing applications with electrogenic cells^222^ (Figure 5E).

Similar to conventional silicon-based FETs, OFETs operate by modulating the flow of electrons or holes through an organic semiconductor channel using a gate voltage. In these devices, the gate electrode directly contacts a dielectric layer (e.g., SiO_2_, Al_2_O_3_ or insulating polymers), which, in turn, interfaces with a thin organic semiconductor film that works as the channel of the OFET. Their compatibility with conventional electronic circuits and suitability for integration with flexible substrates make them attractive for neural interfaces. However, their sensitivity to environmental factors and slower response times compared to inorganic transistors limit their effectiveness in high-speed neural sensing applications.^232–234^

EGOFETs, an improvement on OFETs incorporate an electrolyte (liquid or ion gel) as the dielectric gate to primarily modulate the channel electronic conductivity through ionic effects. Hence, the gate bias induces the redistribution of ionic charges in the electrolyte, thereby forming two EDLs in series at the gate-electrolyte and electrolyte-semiconductor interfaces. This enhances capacitance and sensitivity, making them ideal for biosensing applications such as detecting biomolecules and ions. Their low-voltage operation is advantageous for wearable electronics and continuous health monitoring. Yet, shared ionic interactions can complicate independent gating, and like OFETs, they suffer from slower response times.^225^

On the other hand, OECTs operate by allowing ions from the electrolyte to diffuse into the transistor channel, modulating its bulk conductivity. This ion-electron coupling enables volumetric capacitance, resulting in low operating voltages and high sensitivity to ion fluxes, ideal for detecting electrophysiological and neurochemical signals. OECTs are particularly effective for amplifying weak neural activity and are known for their long-term stability in aqueous environments. Conductive polymers such as PEDOT:PSS and its blends support efficient ion transport, enhancing the performance of neural monitoring systems and BMIs.

Although slower than other transistors due to the nature of volumetric ion transport, OECTs offer excellent biocompatibility, inherent signal amplification through high transconductance, and scalability through multichannel integration.^150,151,167,235^ However, their reliance on a shared electrolyte limits independent gating, making them less suitable for complex circuit integration. To overcome this, internal ion-gated transistors (IGTs) embed mobile ions directly within the conducting polymer channel. This enables a self-(de)­doping mechanism that enhances switching speed and transconductance, facilitating their use in bioelectronic circuits such as inverters, amplifiers, and oscillators.^167,236,237^ Successful examples showed how organic transistors can interface neuronal tissue at different scales, achieving single cell monitoring and stimulation as well as surface and implantable probes for brain interfacing.^235,238–240^

Building upon these capabilities, recent advances have focused on integrating photoactive materials into both conventional electrodes and organic 3-terminal devices. These platforms in fact might facilitate wireless powering and stimulation, reducing the need for implanted components and wiring.^241^ Clinically, such systems can minimize inflammation, lower infection risks, and promote faster recovery while reducing costs. In behavioral animal studies, wireless neural devices enable experiments in more natural, sociologically and environmentally relevant conditions, eliminating biases introduced by tethers.^242^

Among these photoresponsive technologies, photovoltaic electrodes have gained particular attention. Traditionally employed in solar energy applications, photovoltaic electrodes convert light into electrical energy via the photovoltaic effect.^243^ These devices typically use semiconducting materials to absorb photons, excite electrons, and generate a current. In this context, the electrode serves a dual purpose: capturing light to generate charge carriers and conducting those carriers to complete an electrical circuit.

In neurotechnology, this principle has been adapted for neural interfacing, most notably in the field of vision restoration. Photovoltaic interfaces have shown promise in treating degenerative retinal conditions such as retinitis pigmentosa and age-related macular degeneration, which lead to the progressive loss of photoreceptors.^244^ By mimicking the function of these lost photoreceptors, photovoltaic electrodes can convert incoming light stimuli into electrical signals that stimulate the remaining retinal neurons, thereby restoring partial visual function.

For example, one study demonstrated that temporally reliable and spatially selective spiking activity could be induced in primary rat embryonic hippocampal neurons cultured on a biocompatible device using pulsed light stimulation (20 ms at 1 Hz, 10 mW mm^–2^ at 532 nm).^221^ Later, the photovoltaic layer was replaced due to concerns about faradaic currents; here, photothermal and photoelectrical effects supported local heating, inducing slower transient responses and influencing membrane capacitance and ion channels.^222^ Furthermore, photoactive layers under prolonged light pulses (500 ms) were used to silence neurons, triggering hyperpolarization and inhibiting both spontaneous and electrically elicited activity.^223^ These results were extended to models ranging from degenerated retinal explants^224^ to dystrophic rats.

More recently, the POLYRETINA epiretinal prosthesis (Figure 5F) was shown to restore high-resolution visual responses *in vivo*.^225^ The implant includes 10,498 units and achieves a 43-degree visual angle coverage, enabled by the conformability of its substrate. As compared to retinal implants based on inorganic semiconductors,^226,227^ organic systems patterned on stretchable substrates achieve wider visual angles, an essential factor in restoring visual acuities above legal blindness thresholds (i.e., 20 degrees). A major limitation, however, is the restricted absorption spectrum in the visible range, compared to inorganic counterparts capable of absorbing in the near-infrared (NIR). NIR wavelengths are preferred for subcutaneous stimulation as they avoid activating surviving photoreceptors, with further advances in NIR-sensitive materials^228,229^ as well as nonfullerene molecules^230^ which are opening new possibilities for neuronal interfacing.

Recently, OEPCs have been exploited as optoelectronic-to-ionic transducers^154,231^ (Figure 5G). They control voltage-gated ion channels via capacitive coupling, generating charges at the heterojunction level upon light stimulation. This creates a potential difference in the surrounding electrolyte, affecting the membrane potential and potentially triggering action potentials. Besides primary cortical neurons and retinal explants,^231^ OEPCs have been validated in vivo in the rat somatosensory cortex^232^ and peripheral nerve,^233^ successfully achieving subcutaneous stimulation. In recent studies, OEPC implantation increased c-Fos expression at stimulation sites, indicating potential for targeting deeper brain areas.^234^ Optoelectronic biointerfaces can be further engineered with material moieties beyond current approaches.^235,236^ For instance, the use of perovskites^245^ and QDs^246^ was demonstrated *in vitro*, although cytotoxicity and bioaccumulation effects are still under investigation.

#### 3.4.3 Optogenetics

These advances in organic and optoelectronic transistors have significantly expanded the toolbox for bioelectronic neural interfaces, allowing for enhanced resolution, flexibility, and bidirectional communication with neural tissue. As the field moves toward more integrated and less invasive platforms, the boundary between active electronics and biological modulation continues to blur. This convergence is particularly evident in the emerging synergy between organic optoelectronics and control of genetically modified neuronal networks. While organic photoactive materials can deliver precise, wireless stimulation through photovoltaic or capacitive effects, optogenetics introduces molecular-level specificity by enabling light-gated control over ion channels within targeted cell populations. Together, these approaches redefine the design space for next-generation closed-loop neural interfaces, seamlessly bridging synthetic devices and genetically sensitized neural circuits.

Due to the accuracy with which stimulation light can be applied to cells and tissues, the expression of light-responsive proteins (optogenetics) has become a primary tool for manipulating neuronal networks. Initially, efforts to light-sensitize neurons focused on azo-benzene modifications of potassium channels,^97^ but the discovery of ChR2 in 2003 ^90^ (Figure 3D-E) revolutionized the field by enabling directly light-gated control of ion channels that could be introduced into neurons without regard for local chemical pathways.^98^ The range of optogenetic tools has expanded^99^ to cover multiple ions,^100–104^ varying activation dynamics,^105–107^ and different light absorption spectra,^107–109^ offering unparalleled precision in controlling neurons. Throughout the rise of optogenetics, there have also been efforts to manipulate secondary messengers with light,^109^ among the most recent using cAMP to potentiate synapses via the blue light activated bPAC.^41^ Corresponding supporting technologies have also been developed to bring light to the optically sensitized neuron. Optrodes replace one or more of the recording channels in a multielectrode array with an optical fiber to deliver light^110–112^ (Figure 5H). Other MEAs have long sought to incorporate a nearby light-source,^113–115^ facing challenges of heating, optical focus, and electrical cross-talk between driving currents and sensing elements. Significantly more progress has been made in all-optical approaches, where optogenetic stimulation or inhibition is coupled with imaging of neuronal activity.^116^ Furthermore, recent results raise questions if blue light could potentiate synapses in the absence of optogenetic constructs.^117^

Emerging technologies, such as NeuroART,^118^ are revitalizing closed-loop optogenetic systems for real-time recording and manipulation. Alternative light-based systems, like PIF pairs,^119,120^ photocaged neurotransmitters,^96^ and photoactivatable receptors^95^ have been developed to control signaling pathways beyond ion channels. Additionally, azobenzene-based systems offer synthetic light sensitivity, manipulating proteins and even mechanical properties of materials in response to light. Although channelrhodopsins dominate the field, these alternative methods provide diverse ways to influence cellular behavior using light.

#### 3.4.4 Ion Pumps

In biological systems, ion pumps are membrane-spanning proteins with alternating gate mechanisms that actively move ions against concentration gradients. The energy for this process is derived either from ATP hydrolysis (primary pumps) or pre-existing ion gradients (secondary pumps). These pumps are essential for maintaining osmotic balance, generating membrane potentials, and facilitating signal transduction.^237^

Inspired by nature, artificial ion pumps have been developed to regulate ion flow in synthetic systems. Applications range from drug delivery^238^ and energy conversion^239^ to more biologically integrated functions like plant biorhythm regulation^240^ and human-machine communication.^241^ Among these, the most impactful innovation for neurotechnology has been the development of pumps that can interface directly with neurons, enabling precise control of ionic environments and neural activity.

Ion pumps designed for this purpose include several types, but electron-driven systems have emerged as particularly promising tools for neuronal interfaces due to their electrical tunability and compatibility with soft, biocompatible materials.

One such approach is the OEIP, which employs materials like PEDOT:PSS to transport charged species across membranes using externally applied electric fields.^238,246,247^ This method allows for highly localized and temporally controlled delivery of biologically relevant ions and molecules. A PEDOT:PSS-based OEIP capable of delivering potassium ions to neurons was demonstrated. The targeted release of K^+^ ions depolarized the neuronal membrane, thereby activating voltage-gated Ca^2+^ channels.^248^ This kind of electronic control over ion signaling enables modulation of neuronal excitability with minimal invasiveness.

These devices exploit the dual conduction properties of conjugated polymers like PEDOT:PSS, which support both electronic and ionic transport. This makes them ideal for bridging the communication gap between digital electronics and soft biological tissue.^249^ Unlike traditional electrodes, OEIPs can deliver neurotransmitters or ions without generating Faradaic reactions, reducing the risk of tissue damage and inflammation.

Extending this concept further, OEIPs have been used to deliver neurotransmitters such as glutamate, enabling synthetic synaptic-like behavior. One design used overoxidized PEDOT:PSS as the channel and standard PEDOT:PSS as electrodes. Upon applying voltage, glutamate was electrophoretically pumped through the channel.^246^ This biomimetic delivery mechanism offers a route to developing artificial synapses and neuromodulatory systems with high spatial precision.

While other pump types, such as pH-gradient or light-driven pumps, are primarily explored in broader nanofluidic and sensing applications, they also hold potential for neural interfaces. For example, light-driven ion pumps can offer remote, noncontact control over ionic flows. A nanopipette system with photoactive PbS QDs and PEDOT:PSS was developed that produced ionic currents in response to visible light.^245^ Although not directly used for neural modulation, similar systems could be adapted for optically controlled neuromodulation in the future, offering new modalities for wireless brain interfaces.

Naturally occurring protein-based ion pumps, such as those found in unicellular organisms, have also contributed to the field through optogenetics. These light-activated pumps can be genetically targeted to specific neurons and used to manipulate membrane potential independent of endogenous ion gradients,^250^ as described in the paragraph 5.4.4. However, practical challenges such as the need for high light intensity and the risk of cytoplasmic acidification limit their standalone use in neural control, often favoring optogenetic channels instead.

Altogether, bioinspired ion pumps, particularly electron-driven systems, are shaping a new generation of neurotechnology. By enabling seamless ionic control at the cell interface, they offer powerful tools for modulating neural activity, studying brain function, and developing advanced therapeutic systems.

### 3.5 Modalities in Neural Interfaces

Neurons and synapses primarily communicate through electrical and chemical signaling. However, other stimuli, such as mechanical, thermal, or optical, can also modulate their activity by engaging specialized ion channels or disrupting the system. As discussed earlier, neurons and synapses combine various signaling modes to form a complex, multimodal communication network. To achieve seamless integration with this network, interfacing technologies must be capable of sensing and responding to this diverse range of stimuli. In response to this need, multimodal neural probes have been developed that can simultaneously record (“sense”) and modulate neural activity using different modalities, such as electrical, chemical, and optical signals.

#### 3.5.1 Electrical Signal Monitoring and Stimulation

Sensing (recording) or modulating (stimulating) neural activity using electrical methods involves converting ionic currents into electron charge carriers, and vice versa, at the interface between the electrode and the neuronal electrolyte (Figure 4A_iv_). This interface is typically modeled by the EDL, represented as a capacitor in parallel with a resistor. In this transduction process, ions move through the electrolyte (ionic conductor), which may be the cytoplasm in intracellular coupling, the medium in an *in vitro* culture, or the extracellular matrix surrounding neurons in tissues, while electrons flow through the electrode surface (electronic conductor). Consequently, the charge transfer at this interface may occur via two main mechanisms: capacitive, involving electrostatic forces that charge and discharge the EDL capacitor, and faradaic, involving redox reactions. Hence, the dominating mechanism depends on the material and geometry of the electrode, as well as on the properties of the EDL.^250,251^

Sensing neuronal electrical activity entails detecting changes in membrane potentials, which reflect the fluctuation of ionic concentration gradients that create charge differences between the intra- and extracellular space of a neuron. Thus, when discussing neuronal electrical activity, we refer to fast potential changes in the form of subthreshold potentials or action potentials, as well as lower-frequency signals representing the combined activity of groups of neurons, known as LFPs. Modern methods, such as the patch-clamp technique developed in the 1970s, represent a breakthrough in electrophysiology. This technique enables highly precise measurement of ionic currents across the cell membrane of individual cells by forming a tight seal – known as gigaseal resistance – between a glass micropipette and the cell membrane.^252^ This seal allows for accurate sensing of intracellular potentials, including both subthreshold and action potentials. While the patch clamp method is widely used both *in vitro* (in dissociated neuronal cultures or neural slices) and *in vivo*, it is a highly invasive technique that requires direct contact with the cell’s cytoplasm. Additionally, it relies on microscopy techniques (e.g., optical or two-photo imaging) to target cellular and subcellular structures individually, limiting its use for parallelized, high throughput, and long-term recordings.^219,220,253–255^

Contemporary approaches for neuronal recordings have advanced from single-electrode techniques (e.g., patch clamp method) to high-density arrays with hundreds of thousands of electrodes.^266–270^ This expansion in recording capabilities is made possible by tools such as MEAs and transistor arrays, often in combination with CMOS technology. These tools have enabled primarily extracellular recordings whose signal quality depends on the close contact between the recording electrode and the neuronal target as discussed earlier. Advances in micro- and nanotechnology have allowed the engineering of the electrode-neuron interface *in vitro* with vertical nanostructures or its combination with nanocavities. These interfaces can be used to form tight seals with high seal resistances (between 10 and 400 MΩ) that enhance the SNR of extracellular recordings by increasing recorded potential amplitudes from tens to hundreds of μV or even mV.^171,187,256,257^ In this regard, MEAs with vertical nanostructured electrodes have enabled the recording of intracellular signals via mechanical poration, electroporation, or optoporation of the cell membrane,^182,253,258^ as well as the noninvasive recording of intracellular-like signals including subthreshold potentials and action potentials due to tight engulfment.^187,256,257,259^

When addressing 3D neural structures, such as neural slices or intact organs, the quality of neuronal recordings depends on several factors. One factor is the proximity between the neural target (generalized point-contact model) and the electrode, which can be compromised by FBRs or microglial insulation.^190,260^ Another crucial factor is the electrochemical properties of the recording electrodes, particularly their impedance, which is in turn dependent on the electrical properties of the tissue (e.g., its resistivity) and the electrode geometry, surface topology, and charge transfer mechanisms inherent to the electrode material.^261,262^

When carrying out electrical stimulation, the delivered charge activates voltage-gated ion channels, causing changes in transmembrane potential. This results either in the depolarization or hyperpolarization of neurons, depending in turn, on the polarity of the delivered charge.^263,264^ When capacitive effects dominate charge injection, a reversible process occurs. Charge redistribution happens as the negatively charged electrode attracts cations and repels anions, charging the EDL capacitor, which is in turn discharged when the electrode’s polarity is reversed. As a result, charge transfer is governed by electrostatic forces, with no electron transfer involved. Moreover, capacitive charging can occur through electrolytic processes in which charge is stored in thin oxide layers that possess high dielectric constants.

Faradaic stimulation involves electron transfer mediated by redox species at the EDL, leading to reversible or irreversible reactions. Reversible reactions, governed by kinetics, occur when electron transfer is faster than mass transport of electrochemical products. These products remain at the electrode surface and can be reversed with a change in polarity, leading to effective charge storage. Irreversible reactions, governed by mass transport, occur when redox species diffuse away before being fully reacted. This results in no effective charge storage and can cause changes in the chemical composition in the surroundings at the EDL, potentially leading to electrode degradation due to corrosion or tissue damage due to pH changes. Comprehensive examples of irreversible reactions are described in other reviews,^144,264^ however, the electrolysis of water is a potential irreversible faradaic reaction that may occur to all electrodes.^265^ During faradaic charge transfer, staying in the regime of reversible faradaic reactions is of utmost importance. The faradaic reaction is then irreversible if the electrode is driven far beyond its equilibrium potential. In the case of the electrolysis of water, beyond a certain threshold potential, all electrodes will produce hydrogen gas and hydroxyl ions upon the reduction of water, and oxygen gas and hydrogen ions that impact the pH of the surrounding upon the oxidation of oxygen. This threshold potential is the so-called ‘water window’, defined as “the potential region between the oxidation of water to form oxygen and the reduction of water to form hydrogen”.^264^ Hence, electrodes must not be driven to potentials beyond the water window during electrical stimulation. Furthermore, some electrode materials exhibit charge transfer mechanisms driven by both faradaic and capacitive processes, namely pseudocapacitive reactions. In this case, faradaic reactions are bound to the electrode surface, forming, in turn, effective charge storage at the surface while still enabling the faradaic electron transfer.^144,264^

Electrical recording and stimulation, used as sensing and actuation to monitor and modulate neural activity, offer powerful means to understand and characterize neural function, as well as to treat or restore impaired or lost neural functions. Applications of electrical recording *in vitro* comprise the investigation of circuit dynamics and electrophysiological phenotyping in engineered neuronal networks in dissociated and induced pluripotent stem cell-derived neuronal cultures, spheroids or organoids, and neural slices *in vitro*.^271–273^ Furthermore, *in vivo* neural recordings in laboratory animals have allowed the long-term tracking of individual neurons during the adult life of mice^274^ as well as the monitoring of neuronal ensembles across brain regions.^214^ In a clinical setting, neuronal recordings have enabled a paralyzed patient to wirelessly and accurately control a computer mouse, allowing them to play video games.^275^

Moreover, some applications of electrical stimulation *in vitro* include the investigation of diverse electrical stimuli to understand their influence in network connectivity in neuronal cultures,^276,277^ the engineering of 3D neural tissue,^278^ or the investigation of electrical stimulation protocols for neural restoration in neural slices, such as the retina.^279,280^ Furthermore, clinical applications that have experienced notable advancement due to electrical stimulation include the restoration of location in paralyzed subjects,^281^ unidirectional or real-time feedback and adaptive DBS therapies for Parkinson’s disease,^282,283^ the use of cochlear implants for the restoration of hearing,^284^ and retinal prostheses for the restoration of useful vision in blind patients with degenerative retinal diseases.^285^

#### 3.5.2 Biochemical Sensing and Actuation

As mentioned earlier, the transmission of intercellular signals in nervous tissues occurs via alterations of the cell potential governed by the release of neurochemicals such as neurotransmitters. The quantitative determination of the concentration variations of these substances is very intricate due to the huge variety of these chemicals, their different chemical nature, their complex interrelation, as well as their action on different time and length scales. A large spectrum of different techniques can be used to study a particular chemical under defined conditions. However, there is no universal method that can decrypt the full complexity of chemical signaling in the brain.^286^ On a large scale, during brain-wide or brain slice studies, imaging techniques can be used providing information on brain activity, metabolism, and molecule distribution in large functional domains.

However, biochemical sensing represents a widely used approach to sense different species in the neuronal networks from single cell to organ level. For these purposes, common platforms comprise four main components including the analyte to study, the receptor which selectively interacts with the analytical target, a transducer that converts this interaction into an electrical signal, and the electronic interface that amplifies and digitalizes the sensor signal.

A wide variety of different receptors has been utilized in neuronal biochemical sensing with natural origins such as antibodies and enzymes or synthetic moieties as for instance aptamers, imprinted polymers, and inorganic catalysts^287^ (Figure 6A). However, some neurotransmitters, such as the catecholamines dopamine, adrenaline, and noradrenalin but also serotonin, epinephrine, and norepinephrine can be directly measured *in vitro* and *in vivo* by amperometric detection schemes via individual electrode fibers^288,289^ or high-density electrode arrays^290^ without the need for any receptor as they can be electro-oxidized.

**Figure 6:**
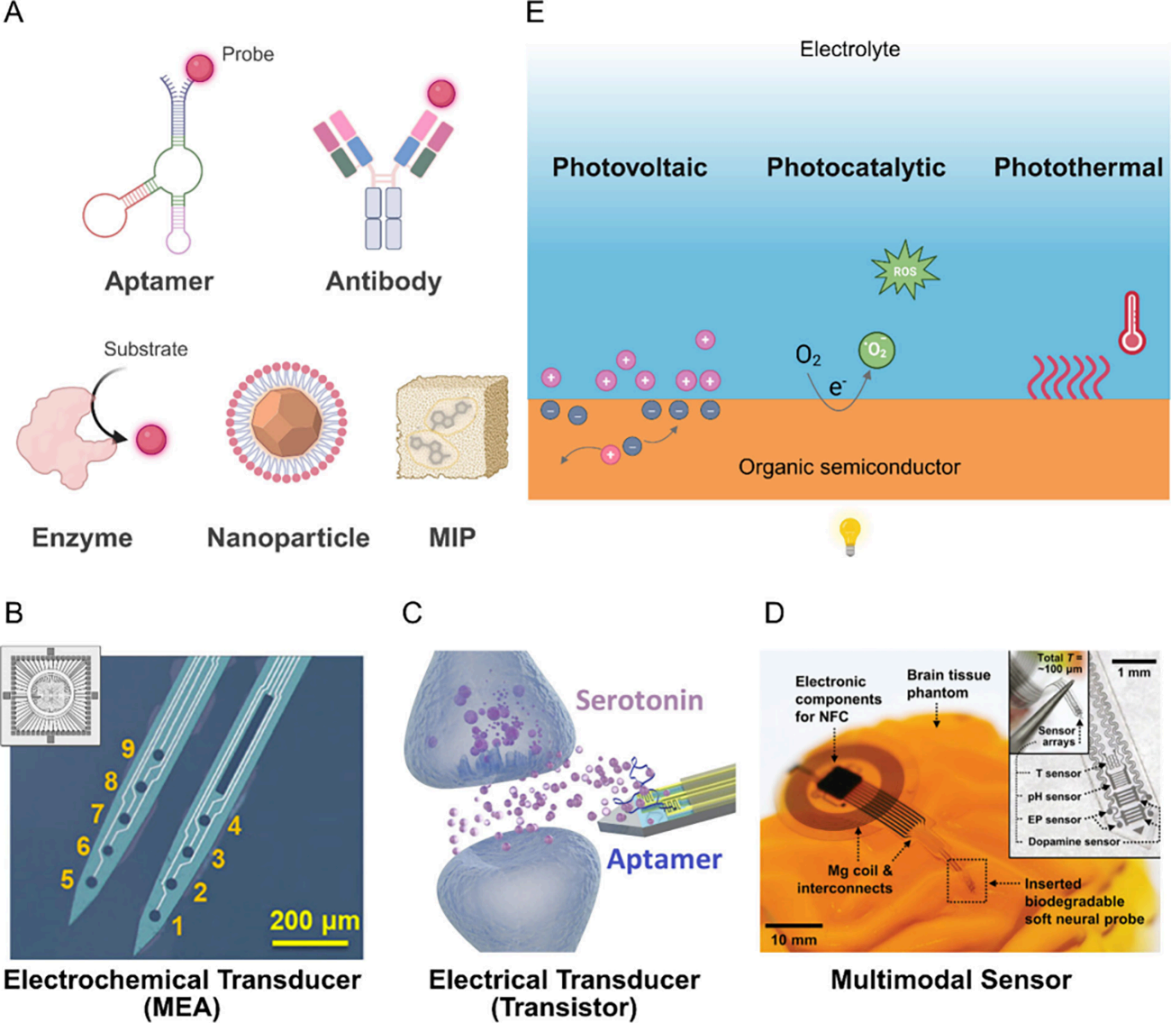
**A.** Schematic representations of receptors implemented in biosensors for the detection of neurochemicals including aptamers, antibodies, enzymes, nanoparticle catalysts, and molecular imprinted polymers. Created in BioRender. Santoro, F. (2024) https://BioRender.com/y36p610. **B.** Micrographs of a modified MEA for deep brain implantation and dopamine detection (adapted with permission from reference 340). Copyright 2012, American Chemical Society. **C.** Neuroprobe with two In_2_O_3_ FETs at the tip. Illustration showing release of serotonin in the extracellular space monitored by an aptamer-FET neuroprobe. (adapted from reference 317). Copyright 2021, American Association for the Advancement of Science. Distributed under a Creative Commons CC BY License (CC BY 4.0). **D.** Silicon-based brain-integrated probes with arrays of electronic sensors for neurochemicals and neurophysiologies in the deep brain. Inset: Image of the neural probe with a conventional syringe needle (D, ≈1 mm) for comparison (adapted with permission from reference^341^). Copyright 2022, Wiley-VCH GmbH. **E.** Upon light illumination, three main phenomena - namely photothermal, photocatalytic and photovoltaic conversion - can occur in an organic semiconductor immersed in an electrolyte; the consequent generation of heat, ROS or electrical charges, respectively, may determine neuronal activation (adapted from reference 342). Copyright 2021, Springer Nature. Distributed under a Creative Commons CC BY License (CC BY 4.0).

The recording of these amperometric NT signals allows for the detection of the exocytosis of synaptic vesicles even on the location of individual synapses and with millisecond temporal resolution.^291^ Therefore, mainly fiber-like microelectrodes and multielectrode arrays made from carbon materials or coated with conductive polymers (Figure 6B) are positioned near the cell under study and the electrode is polarized with a potential more anodic than the oxidation potential of the target molecule. By recording the oxidation current over time, the dynamics of the exocytosis as well as the number of released molecules can be determined.^292^ Although used in *in vivo* settings, this technique does not distinguish between different oxidizable agents and provides not much information about the chemical nature of these molecules.^293^

Another versatile electrochemical technique for the detection of neurochemicals is FSCV, which offers the advantages of high temporal resolution, the capability to distinguish between chemicals, and easy technical implementation. Therefore, the potential of the electrode is cycled by a triangular waveform which results in a neurochemical-specific oxidation and reduction pattern. Since the potential is swept with rates of several hundreds of volts per second, subsecond concentration variations can be recorded *in vivo* with acute and chronic electrodes.^294^ The lateral resolution is defined by the dimensions of the recording electrode. Single-cell recordings are common practice for both amperometric detection and FSCV in *in vitro* and *in vivo* modalities. However, the waiving of a receptor layer brings a drawback to these techniques. Adsorption of the oxidation products and accompanying fouling in the complex biological matrix impairs the live time of these sensors.^295^

Alternatively, sensors modified with enzyme-containing receptor layers have been developed. Here, the enzymes provide a high selectivity as they were naturally developed for conversion of their target molecules (i.e., substrate, cofactor) and facilitate the detection of nonelectroactive species as they generate electroactive molecules that can be electrochemically detected, see above. Representative examples are enzyme-based sensors for glutamate using glutamate oxidase (GlOx)^296^ or glutamate dehydrogenase,^297^ acetylcholine using acetylcholinesterase,^298^ ATP using glucose oxidase and hexokinase or dopamine utilizing tyrosinase.^299,300^ The enzymes need to be immobilized to or in proximity to the electrode which can alternate the performance of the sensor.^301^ Furthermore, they can easily degrade under nonphysiological experimental conditions and the implementation of miniaturized and scalable sensors is still in its infancy. Therefore, synthetic enzyme-like nanomaterials (nanozymes)^302^ have been developed that can convert nonelectroactive neurotransmitters and facilitate their electrochemical detection. One example is the oxidation of glutamate to oxoglutarate^303^ via Ni nanowire array electrodes. Oxytocin was detected by using BDD microelectrodes via a chronoamperometric method combined with flow injection analysis.^304^ Recently, a carbon fiber electrode was modified with a single-atom catalyst facilitating the *in vivo* sensing of dopamine with a lateral food print of less than 50 μm and a temporal resolution of a few seconds.^305^ However, the number of reported sensors is limited to a few neurotransmitters as the establishment of suitable pairs of NT and catalyst is intricate.

In recent years, another type of synthetic receptor has gained interest, namely aptamers as they can be specifically engineered for a respective target independent of its nature. Aptamers are mostly short single-stranded DNA or RNA molecules isolated from nucleic acid libraries via the SELEX process.^306^ To date, aptamers have been reported that recognize various NTs for instance dopamine, serotonin, epinephrine, histamine, ATP, glutamate, or neuropeptide Y.^307^ Compared to antibodies, aptamers can be chemically synthesized at low cost with minimal batch-to-batch variation, have a lower immunogenicity and higher thermostability under harsh conditions. Importantly, the binding profile of an aptamer can be precisely controlled by employing different selection strategies and manipulating the selection conditions during the SELEX process, promising a tunable binding specificity.^308^ Furthermore, the engineered sequences of aptamers are endowed with programmable structures, enabling conformational flexibility and diverse structure-switching functionalities, such as aptamer splitting and target-induced strand displacement.^309^ The easy modifications of aptamers by various anchor and signal tags^310^ can facilitate diverse applications by integrating these receptors into diverse transducer systems. Electrochemical transducers have been discussed already in the scope of the direct oxidation of electroactive NTs and are also the most used concept for the development of aptamer sensors with *in vivo* modalities based on ion current rectification in micropipettes,^311^ carbon fiber electrodes,^312^ metal MEAs^313^ and others. Interestingly, there have been reports for EC sensors that utilize aptamer receptors for dopamine and other electroactive molecules although they could be directly detected by electrooxidation.^314^ However, the aptamer receptors can be easily implemented into the sensor platform and provide the advantage of a high binding selectivity among analog neurotransmitters. Aptamers are even more advantageous for the detection of electrochemically silent neurochemicals such as the excitatory NT glutamate. To facilitate the electrochemical recording of this substance, a redox tag was attached to the aptamer that signals the NT-aptamer binding via a conformational rearrangement of the aptamer on the electrode surface and a variation in the current response of the redox tag.^315^ The size of the electrodes can be as small as 10 μm while the temporal resolution is typically in the range of few minutes.^313^

Besides amperometric, impedimetric, and voltametric transducers, also electrical transducers are frequently employed for the detection of neurochemicals. Above all FETs are used as they feature high sensitivity based on intrinsic signal amplification, scalability, and compatibility with microfabrication. FETs that implement nanomaterials as channel have demonstrated fM detection limits and wide detection ranges for neurochemical analytes^316^ due to high surface-to-volume ratios or quantum effects (Figure 6C). Some of these sensors were manufactured in CMOS processes and facilitate a high device density with a superior sensitivity of ∼ 1 V/fM and large numbers of readouts at the same time. FET-based sensors seem to be well suited as highly integrated probes for *in vivo* recordings as they facilitate both the measurement of electrophysiological and biochemical signals.^317^ Noteworthy, the chemical and structural composition of the channel material remains unaltered during the sensing process, merely the electrical properties (e.g., impedance) of the channel vary. Chemical recognition is typically achieved via the immobilization of the same types of receptor as discussed for electrochemical transducers on to the transistor channel (paragraph 5.4). The lateral footprint of the transistors is typically larger than for microelectrodes as the device architecture is more complex while the temporal resolution is mainly determined by the binding kinetics of the receptor which can be in the millisecond range.^318^ The same is true for transistors made from synthetic semiconducting molecules or polymers have been utilized for biochemical sensing in neuronal tissues. Here, OFETS and OECTs have been developed as biochemical sensing elements,^319^ permitting the detection of biochemical signals with high sensitivities and fM detection limits due to their high transconductance characteristics^320^ as demonstrated for dopamine sensing. It was also observed that the electrical properties of the polymer channel could be tuned in response to neurotransmitter signals.^321^ Both electrical and electrochemical transducers have been implemented in multimodal devices where electrophysiological and biochemical signals can be recorded at the same time (Figure 6D). Furthermore, a combination of optical stimulation via an micro LEDs or optrodes together with the recording of dopamine signals have been reported in animals.^322^

Finally, the local chemical stimulation by pumping of glutamate was demonstrated in *in vitro* astrocyte cultures via increased intercellular [Ca^2+^] levels and *in vivo* experiments by targeting specific regions of the guinea pig auditory system.^323^ Another work reported on development of an OEIP to efficiently deliver GABA to the spinal cord of rats to treat neuropathic pain.^324^ However, OEIPs are limited by their operation at high voltage, which is prone to induce drug degradation. To overcome this drawback, a microfluidic ion pump was utilized to deliver potassium ions into specific regions of the mouse cortex. With the activation of the pump, obvious hyperexcitability was observed during the EEG recording as a result of potassium ions release.^325^

Epilepsy is caused by an abnormal, excessive, and synchronized electrical discharging of brain neurons.^326^ Incorporating drug delivery and electrophysiological signal could facilitate a treatment approach for this disease. The feasibility of direct in situ electrophoretic drug delivery. They designed a probe consisting of a microfluidic ion pump for an on-demand drug delivery and electrodes for recording local neural activity. GABA was delivered to the hippocampus of mice whose seizures were induced into the animal. After the pumping of GABA, the abnormal electrical discharging was suppressed.^327^

#### 3.5.3 Optical and Optoelectronic Strategies for Neural Interfacing

Neuronal interfacing through optical communication faces challenges related to light-tissue interaction, as penetration depth is limited by scattering and absorption.^328^ As light passes through neural tissue, it scatters in multiple directions due to cellular structures and boundaries. Additionally, various tissue components absorb light at different wavelengths, with water, hemoglobin, and lipids being the primary absorbers in the brain. This absorption can cause localized heating, potentially leading to tissue damage. Phototoxicity varies based on power, wavelength, and stimulation patterns, making careful, application-specific dosing essential.^329^ NIR light generally achieves the greatest penetration depth, while adaptive optics can be used to reduce scattering and enhance focus on deeper tissues.^330^ Additionally, micro-LEDs, optical fibers, and waveguides can be integrated into microfabricated probes for localized optogenetic stimulation delivery.

Beyond established stimulation technologies, light-responsive materials that undergo topological changes are being explored for delivering mechanical cues to cells and tissues,^331^ dynamically modulating the cellular microenvironment.^332^ While these materials are currently used to promote stem cell differentiation and for mechanobiological studies, they hold potential for novel interactions and stimulation of neural tissue.

Furthermore, photochemical reactions, particularly the reduction of oxygen to superoxide and hydrogen peroxide, have been observed under high-intensity could be induced at the device-neuron interface^333^ (Figure 6E). These reactive oxygen species can modulate cellular processes,^334,335^ though their potential toxicity at high concentrations is a concern.^336^ Additionally, local photothermal effects inevitably occur with light absorption (Figure 6E). When kept within safety limits, these effects can induce physiological responses, such as temperature-dependent membrane depolarization^337^ or the activation of temperature-sensitive ion channels.^338^

Photoelectrical effects are typically classified into photocapacitive and photofaradaic processes^339^ (Figure 6E). Photocapacitive stimulation involves charge redistribution without electron transfer, whereas photofaradaic processes involve electron transfer across the electrode–electrolyte interface, leading to redox reactions. Photocapacitive stimulation is generally considered safer due to its reversible nature, as it avoids the chemical reactions that can degrade electrodes and produce potentially harmful chemical byproducts.

## 4 Neurohybrid Biointerfacing

### 4.1 General Considerations

Coupling neuromorphic computing devices with neural devices and probes involves addressing several important factors to ensure seamless integration and effective communication between the neural interface and the neuromorphic system. Some examples on biointerfacing with neuromorphic devices have been successfully shown for signal classification and stimulation, holding promise for tackling into specific neuronal probes coupling. Neuromorphic biosensors have been already exploited for classification of biosignals, for example for the measurements of ions in solution and the training of an hardware neural network to establish the positivity to cystic fibrosis from the analyzed sample.^343^ A complementary circuit based on OECTs with neuromorphic features has been demonstrated to show ion-modulated spiking, while able to interface with Venus Flytrap (Dionaea muscipula) to induce lobe closure upon input current stimuli.^344^

While we have extensively discussed previously the relevance of biocompatibility and integration of neural interfaces, here we will discuss the main features of neuromorphic platforms to be directly coupled to neural probes.

A key aspect is to use the neuromorphic system to process signals of different kinds coming from the neuronal interface in real time to provide fast response toward a subsequent mean of actuation or stimulation.

Power consumption is another key factor. Both neuromorphic computing devices and neuronal probes must operate with low energy use, especially for systems that require long-term implantation. Neuromorphic computing brain-inspired architectures are highly energy-efficient, helping to minimize power demands and reduce countereffects like overheating.

The ability to learn and adapt to changes in neural activity is another critical requirement. Neuromorphic systems should be capable of adjusting their behavior based on evolving signals, recalling synaptic plasticity mechanisms. This short- and long- time adaptability should be also supported by high-bandwidth communication between the neuronal probes and the neuromorphic device, handling possibly large data volumes. Additionally, low-latency communication is essential to minimize delays between signal capture and response, which is crucial for applications like neuroprosthetics where timing is critical.

Similarly, robust data encoding and decoding mechanisms are necessary to translate the complex neural signals captured by the probes into information that neuromorphic systems can interpret, and vice versa. This ensures accurate and seamless communication between the brain and the neuromorphic device.

A compact, durable and integrated design is also important, especially for implantable systems.

### 4.2 Materials for Neurohybrid Interfaces

#### 4.2.1 Inorganic Materials

In neuromorphic architectures, the key requirement for materials is their ability to emulate synaptic plasticity and adaptability, mirroring brain-like properties such as nonlinearity, memory, learning, and sensitivity to stimuli. This ability is crucial for replicating the strengthening and weakening of synaptic weights, which in neuromorphic systems is mirrored by nonvolatile memories. These memories allow programmable conductance and the retention of applied information. Initially, inorganic compounds were used in the development of synaptic devices due to their impressive resistance-switching and retention capabilities. Since then, a wide range of inorganic materials has been explored.

Inorganic materials, such as metals and metal oxides, have been extensively used in memristors and memtransistors, utilizing different mechanisms, such as phase transitions, redox reactions, and ion migration. One example is PCMs, where materials like chalcogenides (e.g., GeSe, GeSbTe) switch between amorphous and crystalline phases under electrical pulses. Joule heating induces these changes, where the SET phase causes crystallization, and the RESET phase returns the material to its amorphous state. These materials are popular in neuromorphic computing for their fast-switching speeds, allowing them to emulate­(STDP)^345^ (Figure 7A).

**Figure 7:**
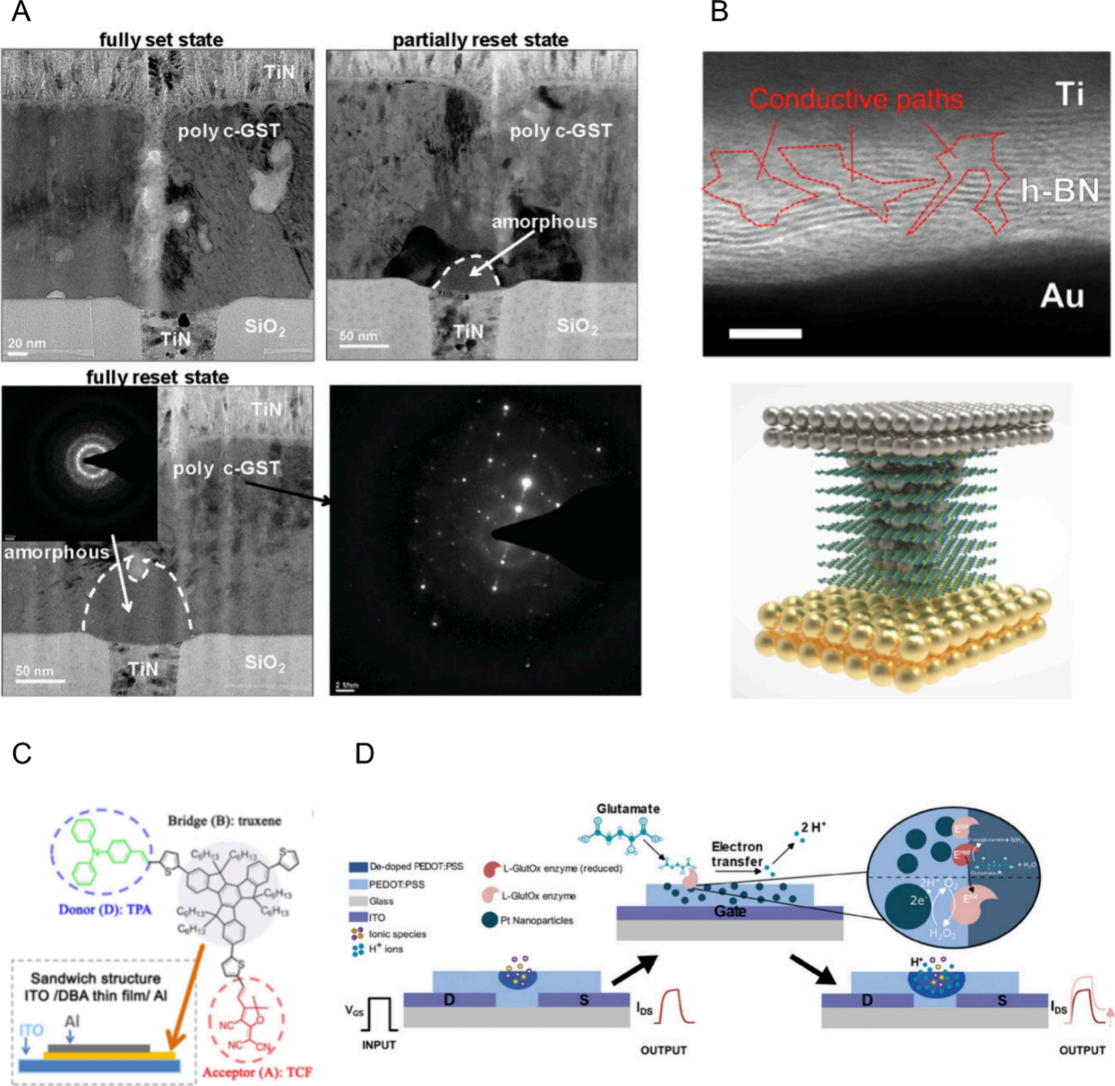
**A.** Transmission electron micrograph of chalcogenide-based PCM during (i) set, (ii) partial set and (iii) fully reset state, and (iv) diffraction pattern of the amorphous region (adapted with permission from reference 345). Copyright 2012, American Chemical Society. **B.** Cross-sectional TEM image of the Au/h-BN/Ti memristor. The local defects responsible for the formation of conductive nanofilaments are indicated in red (scale bar, 5 nm) and represented in the scheme (adapted from reference 352). Copyright 2022, Springer Nature. Distributed under a Creative Commons CC BY License (CC BY 4.0). **C**. Chemical structure of the DBA small molecule, featuring TPA as donor, truxene as bridge and TCF as acceptor; schematics of the sandwich memory device (adapted with permission from reference 359). Copyright 2012, American Chemical Society. **D.** Schematic of the enzyme-functionalized PEDOT:PSS-based OECT; a synaptic stimulus occurs. From left to right: the postsynaptic response when glutamate is not present at the gate/electrolyte interface; the mechanism of the enzymatic reaction occurring at the gate electrode when glutamate is present in solution; enhanced postsynaptic response due to the H+-related polymer dedoping at the channel (the darkened region highlights a further dedoping) (adapted with permission from reference 357). Copyright 2024, Wiley-VCH GmbH.

Metals and metal oxides are also used in RRAM devices, where conductive filaments are formed in the oxide insulator layer. In CBRAM, metal cations (such as Cu^2+^ or Ag^+^)^346^ migrate to form and dissolve these conductive filaments, whereas filamentary RRAM relies on the dislocation and vacancies of oxygen ions to create conductive paths in metal oxides like TiO_*x*_,^347^ HfO_*x*_,^348^ AlO_*x*_,^348^ and SrTiO_3_.^349^ Nonfilamentary or interfacial RRAM includes an insulating oxide layer between the metal electrode and resistive switching material, creating a more gradual and controlled switching mechanism.

In addition to inorganic materials, atomically layered 2D materials^350^ like graphene, TMDs, and h-BN have emerged as promising candidates for memristive devices^351,352^ (Figure 7B). Moreover, monolayer MoS_2_ has been shown to function as a light-sensitive channel material capable of optically resolving neuronal voltages via exciton-trion modulation.^353^ These materials exhibit low-power switching, optoelectronic properties, and high tunability through surface functionalization, making them particularly suitable for neuromorphic applications. The ability to tailor these materials’ electrical properties, combined with their flexibility in device configurations, makes them ideal for addressing the complexity of brain-like computational systems.

#### 4.2.2 Organic Materials

Following the extensive works on inorganic synaptic devices, the discovery of nonvolatile behavior in organic semiconductor-based devices allowed to expand the possibilities for artificial synapse creation. The unique advantages of organic semiconductors, which are lightweight, flexible, and stretchable properties combined with compatibility with low-cost, simple fabrication processes, have positioned them as strong contenders to replace conventional inorganic compounds in electronic devices. Additionally, organic materials offer nearly limitless possibilities for structural modification, allowing for tailor-made properties suited to specific applications. Their organic and soft nature also makes them ideal for interfacing electronic devices with living cells and tissues, due to their inherent biocompatibility and mechanical properties that closely align with biological systems.

Alongside the development of inorganic-based memristors, organic semiconductors have been explored for nonvolatile memories and resistive switching devices, giving rise to the field of organic neuromorphics. Unlike traditional insulators, organic compounds feature conjugated π bonds, where electrons and vacancies are delocalized across the molecule, allowing for charge mobility. Furthermore, these systems can form aggregates via π-π stacking, enabling conduction between molecules. This opens the door for a variety of semiconductive materials, including polymers, small molecules, *D-A* systems (Figure 7C), organometallic complexes, and organic ferroelectric materials, to be integrated into memristive devices, utilizing similar switching mechanisms to their inorganic counterparts.^354^

Small organic molecules have been integrated into memristive switching devices by leveraging charge transfer mechanisms in *D-A* systems. In these systems, the donor moiety transfers the applied stimulus (electrical or optical) to the acceptor moiety, resulting in a higher conductance state. By designing single molecules with multiple acceptor units, multilevel memory devices can be achieved. Additionally, blends of organic compounds have demonstrated nonvolatile memory functions in two-terminal architectures, offering greater tunability by exploiting the individual properties of each component. This can involve either charge transfer mechanisms or charge trapping, where specific sites in the material capture charge carriers. Alongside small molecules, semiconductive polymers have been explored as active layers in two-terminal memristive devices, operating through mechanisms such as metal filament formation, redox reactions, charge trapping, and ion migration.

A major advancement in organic neuromorphic devices comes from the development of OMIECs, which are capable of transducing both ionic currents and electrical signals. This dual conduction mechanism is valuable for a wide range of applications, including batteries, (bio)­chemical sensors, light-emitting electrochemical cells, ion pumps, and neuromorphic systems. Indeed OMIECs can communicate with neurons through ion fluxes as it happens in biological synapse.^355^ For example, PEDOT:PSS. has demonstrated significant synaptic functions in both two-terminal memristive devices and OECTs (Figure 7D).^356–358^

#### 4.2.3 Advantages and Challenges in Materials Design for Neuromorphic Platforms

As mentioned in the previous paragraph, there is an extremely wide range of materials used in neuromorphic platforms, both inorganic and organic, each with its own strengths and weaknesses. This variety allows for the selection of different materials and device architectures based on specific requirements. For example, in neuromorphic computing frameworks, important considerations include device performance metrics such as switching speed, programming time, energy consumption, accessible conductance states, and cycling endurance. These factors are key when choosing the most suitable materials and architectures.

PCMs show very low switching times of ∼ 500 ps, correlated to the crystallization dynamics of the active layer and hence its melting temperature in the range of ∼ 600 °C. On the other hand, switching speed in CBRAMs is in the range of ∼ 100 μs, being dependent on metal ion movements kinetics, while filamentary RRAMs can achieve faster speed in the range of ∼ 10 – 100 ns, thanks to the higher mobility of oxygen vacancies, as opposed to interfacial RRAMs that show the slowest dynamics up to ∼ 1 ms due to high energy barriers to induce the switching.^360^

In such cases, it has been shown that the geometry of the device, including the active layer’s area and thickness, influences its switching properties. As a result, careful device design can enhance overall performance. In this regard, 2D materials can provide a step forward in improving switching times, although they display a wide range of speeds from 10 ms^360^ down to 10 ns.^361^ In addition, organic semiconductors also show a wide range of switching speeds according to the specific employed material and device architecture, given the great variety of available compounds.

Inorganic-based two- and three-terminal devices typically require relatively high voltages, ranging between −3 V and +5 V for memristors and up to 10 V in memtransistors,^362^ according to the selected materials and architectures, therefore requiring high power for synaptic events (e.g., for SET and RESET switching^363^). Nevertheless, recent works demonstrated very low energy consumption as, for instance, in GOQDs stacked with ZHO memristors,^364^ and ultralow power heterostructure composed of Ag/MoS_2_/HfAlO_*x*_/carbon nanotube, achieving 1.9 fJ per spike event, lower than biological neurons.^365^ However, high voltages and high power consumption enable fast switching and programming speeds, therefore this existing trade-off can be tuned according to the device needs.

Similarly, memory switching devices based on organic compounds, small molecules, polymers and hybrid organic–inorganic blends,^354^ operate at roughly the same voltages range of the inorganic counterparts. Interestingly, the high versatility of this class of compounds in terms of molecular structure and fabrication techniques, provides eventually a wide range of possibilities, leading to the lowest power-consuming artificial synapse of ∼ 1 fJ per synaptic event in a nanowire synaptic transistor architecture.^366^ At the same time, memristive materials have demonstrated significant potential for neuromorphic biointerfacing, offering both computational and adaptive capabilities while processing biosignals in real time. Their integration in bioelectronic systems has been explored for applications such as bidirectional neural interfacing and low-power bio-AI fusion, further expanding their role in neurohybrid platforms.^367^

On the other hand, utilizing the mixed conduction of OMIECs holds great promise for creating synaptic devices with low power consumption, especially when used in OECTs, thanks to their operation at voltages below 1 V.

When considering the stability of the various neuromorphic and synaptic devices, particularly regarding cyclic endurance, a wide range of studies have demonstrated excellent performance across different architectures and material choices. This is especially true for computing applications, where the devices are operated in “dry” conditions. However, as we move toward the implementation of neurohybrid interfaces, which aim for seamless integration between electronic devices and living tissues while mimicking biological behaviors, organic materials stand out as the ideal candidates. Since neurons and the brain function in aqueous environments and communicate through the exchange of ions and molecules (such as neurotransmitters) with very low power consumption, organic-based synaptic devices offer these same characteristics. Additionally, they provide intrinsic biocompatibility as well as biointegration, are crucial factors for successful biointerfacing, as explained in paragraph 5.2.

#### 4.2.4 Neuromorphic Biomaterials for Improved Neural Interfacing

As we advance the mimicry of real neurons and brain-like behavior in neural interfaces and neuro-hybrid systems, biocompatibility becomes a critical factor in selecting materials. Devices that interact with living tissues and cell cultures must be nontoxic, preserving cell viability and avoiding inflammatory responses, especially when used in implants. While metals like gold and platinum are well-known for their biocompatibility, many commonly used inorganic materials in neuromorphic platforms can be toxic or have uncertain toxicity profiles. This concern has driven significant research in recent years toward developing neuromorphic devices made from organic semiconductors and nature-derived materials, which are considered ideal candidates due to their natural compatibility with biological systems. Additionally, the stability of electronic devices in aqueous environments is crucial for ensuring long-term interfacing. In this regard, OMIECs^368^ have a prominent position being intrinsically stable in aqueous environments and more importantly can emulate the means of communication of neurons through ion fluxes as in biological synapses.

As mentioned, in 5.3.1, The physical interaction between cells and devices also plays a key role in transmitting electrical and electrochemical signals. In vitro systems often experience decoupling between cells and tissues due to the presence of a physical gap, known as the cleft. Over the past decade, various technological and microfabrication techniques have been developed to engineer nano- and microtopographies, such as 3D and pseudo-3D structures, grooves, and scaffolds, aimed at minimizing this cleft.^369^ These structures promote cytoskeletal rearrangements and plasma membrane ruffling, leading to tighter cell-surface junctions, which are crucial for bioelectronic devices to achieve stronger electrical coupling and improve SNR. Standard silicon-based microfabrication techniques have been successful in creating diverse surface topographies. However, organic semiconductors offer greater flexibility in design and fabrication, allowing for the use of unconventional patterning techniques. For instance, PEDOT doped with PSS^–^ or PF_6_^–^ has shown the ability to form dendritic fibers that mimic neuronal dendrites, potentially guiding neuronal growth and network formation.^367,368,370,371^ These fibers, fabricated using AC-driven electropolymerization, have shown promise in exhibiting memory and synaptic functions, making them valuable in applications like reservoir computing.^372^

Additionally, neurons, particularly synapses, are not static units; they can strengthen or weaken their connections through physical reshaping. As a result, there is growing interest in engineering topographies that can mimic and potentially drive synaptic morphological changes. One promising approach involves using light-responsive polymers capable of undergoing conformational changes when exposed to light, such as azobenzene-based materials. Recent studies have shown that micropillars made of pDR1m azopolymer can be reversibly reshaped into elongated bars, demonstrating the potential for dynamic manipulation of synaptic structures.^332^ Although nonconductive, such microstuctures have been coated with PEDOT:PSS to impart conductivity yielding a light-driven conductive deformable substrate.^373^

### 4.3 Devices and Architectures in Neuromorphic Biointerfacing

Traditional computing architectures rely on a clear separation between processing and memory, where data is transferred through interconnecting buses. While this approach has been effective, the increasing disparity between processing speed and memory access, known as the von Neumann bottleneck, limits overall performance and scalability. As data-intensive applications demand higher efficiency, these limitations become more pronounced, leading to increased latency and energy consumption. Neuromorphic computing offers an alternative by integrating memory and processing within the same system, enabling parallel data processing that more closely resembles the functionality of biological brains. This in-memory computing paradigm significantly enhances energy efficiency and processing speed, making it highly suitable for applications such as brain-machine interfaces (BMIs), neuroprosthetics, and real-time learning systems.^374^ Moreover, neuromorphic systems can integrate multiple sensory inputs, advancing biohybrid devices and brain-machine interfaces.^375^

#### 4.3.1 Three-Terminal Devices and Transistors

Addressing the limitations of conventional computing architectures requires advancements in hardware capable of efficiently handling synaptic operations. Three-terminal devices have been proposed as a viable solution, as they allow independent modulation of memory and processing functions within a single unit. Unlike two-terminal memristors, these devices introduce an additional gate terminal that provides enhanced control over conductance states, improving both energy efficiency and computational scalability.^376^ This additional control facilitates synaptic plasticity, which is crucial for implementing learning and adaptation in neuromorphic systems.^377^

In neuromorphic applications, MOSFETs are fundamental building blocks for circuits that emulate neuron-like behavior. A notable example is the 45 nm CMOS neuromorphic chip, which employs a MOSFET-based architecture to simulate synaptic plasticity and support spiking neural networks (SNNs) that mimic biological synaptic processes.^378^ MOSFETs offer scalability and high integration, making them well-suited for large-scale neuromorphic systems. However, traditional MOSFETs tend to have higher energy consumption compared to emerging alternatives.^378,379^ Additionally, silicon nanowire transistors have been developed to operate at lower voltages. In the realm of inorganic transistors, indium gallium arsenide transistors have shown high electron mobility, which has been exploited for high-speed neuromorphic devices for computing applications,^380^ but not for biointerfacing due to biocompatibility issues. Likewise, ferroelectric field-effect transistors have shown potential in neuromorphic applications thanks to the several nonvolatile states that can be achieved by their channel material,^381^ but the biointerfacing relevance of these devices is exploratory. The latter has been more effectively addressed with TMDs, such as MoS_2_, thanks to their flexibility, biocompatibility and the ability to show neuromorphic features.^382^

#### 4.3.2 Memristors

Memristors, widely regarded as the archetypal artificial synapse, have been central to the emulation of synaptic plasticity and memory functions. Theoretically introduced by Chua in the 1970s as the fourth fundamental circuit element, memristors are nonvolatile memories whose resistance can be modified by various stimuli, including electrical, magnetic, and optical signals. They are implemented in both two-terminal and three-terminal device architectures, providing a wide range of applications.^363^ In two-terminal metal–insulator–metal architectures, the switching mechanism operates between high-resistance and low-resistance states during the SET and RESET phases, producing a characteristic hysteresis loop that modulates the device’s conductance. Three-terminal devices, called memtransistors, add a gate terminal to control the resistive switching of the two-terminal memristor, offering further conductance states.^363^

#### 4.3.3 Organic Transistors

Beyond two-terminal devices, organic field-effect transistor (OFET) can be integrated as artificial synapses exploiting small semiconductive organic molecules and materials at the channel.^383^ Nonvolatile memory operation in OFETs is achieved by inserting an active layer between the gate and the organic semiconductor channel, which serves to store dipoles or charges.^383^ Three-terminal devices like OFETs are particularly appealing for emulating synaptic functions, as the gate terminal can represent the presynaptic input, while the channel modulation corresponds to the postsynaptic terminal, effectively mirroring synaptic weight changes. Various device architectures and organic semiconductors, including both n-type and p-type materials, have been investigated, highlighting the versatility of these compounds.^384^ Conductive polymers offer significant advantages in electrode functionality, particularly in their ability to modulate electrical behavior by leveraging ion fluxes in addition to electron movement. This makes them ideal candidates for operation in aqueous environments, where they exhibit excellent stability and low power consumption.^385,386^ The ionic-to-electronic transduction capability of these materials is especially utilized in organic electrochemical transistors (OECTs), where signal amplification of biological activity is achieved by interpreting the chemical signals generated by cells, rather than purely electrical signals. This approach allows for the collection of more comprehensive, neuroinspired information.^365,382^ OECTs, due to their high transconductance, provide highly efficient local signal transduction while consuming minimal energy. Organic operational amplifiers (OPAs), when integrated with OECTs, can amplify biological signals even at 0 V gate bias by fine-tuning geometric parameters such as channel thickness and the width-to-length ratio.^387–389^ These systems can amplify small signals, such as 100 μV, with power consumption as low as 50 nW.^390^ Given the vast data output from neural recording devices, local data processing becomes essential to reduce memory and battery demands.

Devices are engineered to mimic the behavior of biological neurons and synapses, functioning as synaptic devices that replicate neural plasticity.^358,391^ By configuring the gate as a presynaptic neuron and the source-drain pathway as a postsynaptic connection, transistors can simulate synaptic weight modulation and neural signal transmission, effectively bridging the gap between electronic and biological systems.

Recent advancements in materials like organic semiconductors and oxide-based transistors have further refined the emulation of synaptic behavior, enabling the creation of artificial synapses with learning and memory capabilities.^151,392,393^ These materials allow transistors to convert external stimuli, such as light, pressure, or temperature, into electrical signals, broadening their application in artificial synapse development.^391,394,395^ With high transconductance and the ability to amplify signals at low voltages, transistors are particularly suited for neuromodulation, where precise control of neural signals is critical.^396^

OECTs can effectively mimic both short-term and long-term synaptic plasticity. High-frequency pulses simulate synaptic strengthening by increasing OECT conductance, emulating short-term plasticity, while long-term changes are achieved by retaining charges within the channel. One of the earliest neuromorphic OECTs, based on PEDOT/PEI, successfully replicates long-term synaptic plasticity mechanisms due to its nonvolatile memory properties.^356^ Additionally, OECTs support spatial data integration, enabling functions such as classification. By combining p-type and n-type OECTs, neuromorphic platforms can be created to mimic learning processes like Hebbian learning or firing frequency adaptation in response to stimuli.

There are documented examples in which OECTs emulate learning mechanisms through neurotransmitter oxidation during electrical stimulation. Neurotransmitters such as serotonin, dopamine, and ascorbic acid have been tested in these systems.^397,398^ Moreover, an enzyme-mediated organic neurohybrid synapses has been implemented to achieve neuromorphic behavior driven by nonelectroactive neurotransmitters, such as the glutamate, thanks to the oxidation of hydrogen peroxide.^357^ Such devices have been integrated into organic circuits to trigger the closing of Venus Flytrap lobes in response to repeated inputs or enable robots to use reinforcement learning to navigate a maze.^399^ Mechanisms to control and make the communication between the device and the biological environment bidirectional have been demonstrated with the use of another biologically produced species, hydrogen peroxide, allowing for a closed-loop modulation of the system response.^400^ This application paves the way for a new generation of implantable devices in which the neural response is responsible for learning in artificial systems and, at the same time can be integrated by the system itself in case of a damaged tissue in which some neural connections need to be restored. Recent approaches demonstrated the integration of highly resistive biomembranes with organic transistors to enable synaptic functions mediated by biological ion channels, further bridging the interface between artificial and biological systems.^401,402^ In addition, investigations about the influence of the tissue-like environment can affect the neuromorphic behavior of organic electrochemical transistors.^403^

#### 4.3.4 Crossbar Arrays

The architecture in which the devices are connected can play an important role to achieve an optimization in the elaboration of information, as it happens for the data transfer between the memory and the computing units. In particular, crossbar arrays architecture was proposed to enable parallel processing of analog units, performing vector-matrix multiplication and implementing neural networks.^404^ Their grid-like structure makes the connections between input and output lines efficient. Among the advantages of these architectures, there is the possibility to integrate memristive devices in high-density configurations and scale the network to increase the number of modules allocated. These systems leverage the properties of memristors, which can change resistance depending on the history of voltage applied, similarly to what happens in neurons when the information is transmitted at the synapse level.^405^ By applying read and write pulses, device states change across rows and columns, enabling massive parallel updates. In this way, computations occur in the same place where data is stored, reducing latency and energy consumption.^405^ Moreover, crossbar arrays can perform analog and digital operations, thanks to the possibility to have a gradual or abrupt transition in the conductivity, and then in the state of the system.^406^ For instance, a Ti:PEDOT:PSS:Ti sandwich structure could display a gradual change in the conductance thanks to the modification of the interface between metal and conductive polymer to achieve 100 states and it was shown to be used in crossbar arrays.^407,408^ Access devices like switches or transistors are needed to connect or block memory elements. Key requirements include linear switching for predictable states, low read/write currents for energy efficiency, prevention of sneak paths, and stable conductance states for reliable operation.^409^ For the prevention of sneak path, a device based on poly­(3-hexylthiophene) (P3HT) with photochromic diarylethene was optically modulated to achieve 256 conductance states and it was promising for neuromorphic applications, such as light-assisted programming, overcoming some limitations in electrical memristive-based crossbar arrays without the use of access devices.^410^ In a digital application of crossbar arrays for neuromorphic computing, an organic polymer was modulated, achieving potentiation and depression of the film, creating separate conductance paths in three dimensions, allowing for the formation of two memristive devices on a crossbar-point between four electrodes.^411^ Neuromorphic brain interfaces based on RRAM crossbar arrays have been developed mainly for spike sorting and real-time processing of neural signals.^406^

#### 4.3.5 Spiking Neural Networks

The emulation of brain connections and organization enables artificial neural networks (ANNs) to replicate the parallel computation typical of the brain. However, ANNs operate on hardware that is fundamentally different from the brain, such as Von Neumann machines, which often rely on GPUs and significantly increase power consumption.^31^ The energy consumption gap between ANNs and BNNs is largely due to differences in how information is processed. ANNs run on Von Neumann architecture, which require vast amounts of energy to execute billions of operations per second. These systems rely on synchronized operations controlled by an external clock and process information digitally.^412^

In contrast, BNNs transmit information asynchronously in a mixed analog-digital format, with individual neurons firing independently.^72^ To replicate the brain’s energy-efficient computation model and overcome the mismatch between the synchronized digital operations of Von Neumann machines and the brain’s more efficient communication system, spiking neural network (SNN) circuits have been developed.^32^ In SNNs, neurons communicate in real-time via electrical signals, or spikes, using neuromorphic hardware such as Intel’s Loihi or IBM’s TrueNorth.^32^

However, training SNNs presents a challenge due to their event-driven and nondifferentiable nature, which prevents the use of traditional gradient descent and backpropagation algorithms commonly applied in ANN training.^413^ To address this, brain-inspired methods leveraging stochastic signaling have been developed to improve information propagation and enable the training of SNNs.^414^ For instance, random backpropagation on neuromorphic hardware has achieved task accuracy comparable to ANNs running on GPUs.

Furthermore, recent advancements have made it possible to train SNNs and neuromorphic chips using gradient-based techniques similar to those driving deep learning, opening new pathways for local synaptic plasticity rules. Neuronal spiking communication can also be replicated on biomimetic hardware architectures, such as silicon neurons.^415^ These systems approximate neural activity using spike trains with specific timing, mimicking the integrate-and-fire behavior of biological neurons through components like capacitors and threshold counters. An example is the Axon-Hillock circuit, where a silicon neuron generates a spike when the membrane voltage reaches a threshold.^416^

Moreover, silicon neurons can exhibit synaptic plasticity, mimicking the conductance changes in biological membranes during action potentials, making the hardware circuits more complex and accurate than numerical models.^415^ Among the other computing tasks, SNNs can be used for classification of signals, and in particular biosignals, such as EEG, ECG, and EMG. Temporal patterns can be extracted from those and encoded as spike trains and used to predict the class of the signal.^417^ Different methods are adopted to train SNNs for classification: backpropagation through time can be used to train multilayer SNNs,^418^ while other methods are required for nondifferentiable spike events. Surrogate gradient methods are used to approximate the gradient of a spike function to a smooth differentiable function,^419^ conversion methods translate the trained weight of an ANN into a SNN architecture.^420^ There are also more bioplausible methods to train and update synaptic weight, such as the spike-timing-dependent-plasticity rule.^421^ The event-driven computation and the temporally sparse processing of signals make SNNs efficient and suitable for biological information classification tasks.^422^ Regarding event-driven and temporally dispersed computation in SNNs, these approaches also have a place in the emulation of the senses, e.g., human vision, which is based on highly efficient neural pathways and low-power information coding. Numerous neuromorphic circuits and devices mimicking the function of one or more components of the visual pathway have been developed aiming at achieving biorealistic, real-time, low latency, and low-power machine vision systems, overcoming the limitations of traditional CMOS circuits.^423^ For example, optoelectronic electrochemical transistors (OPECTs), integrating light-responsive materials at either the gate or the channel, can emulate some features of the biological retina. An OPECT with an azobenzene-based gate electrode connected to a PEDOT:PSS resistor was shown to replicate the ON/OFF pathway of the retina.^424^ In darkness, the device mimics the continuous firing of retinal ganglion cells (like glutamate release in biological systems), while under light this firing is suppressed, in analogy with ganglion cells hyperpolarization in response to light. In a more recent work, the retina’s sensory processing was simulated by integrating light-triggered spikes with neurotransmitter-based modulation.^425^ The system consists of a commercial light sensor, the neuromorphic spiking circuit and PEDOT:PSS biohybrid synapse. When exposed to light, the sensor generates a potential, causing the neuromorphic circuit to spike at a frequency proportional with the light intensity, replicating how afferent neurons encode sensory information. The role of the biohybrid synapse is to adjust the spike frequency based on the concentration of neurotransmitter, simulating interneurons signal modulation. Similarly, an optoelectronic neuromorphic circuit was developed able to emulate retinal photopic and scotopic adaptation while exhibiting STP and LTP.^426^ The circuit consists of a photovoltaic divider, using a metal chalcogenide (i.e., CdSe) photosensor and metal oxide (i.e., IGZO) load transistor functioning as artificial retina, and an ionotronic synaptic IGZO transistor as optic nerve. In another work from the same group, mixed QDs (CdSe for red/green and cadmium sulfide (CdS) for blue light sensitivity) were integrated in a IGZO transistor to create multispectral (RGB) color recognition and adaptive chromatic filtering via gate-tunable memory modes.^427^

### 4.4 Biointerfacing

#### 4.4.1 Biosignal Processing and Classification

Neuromorphic systems have seen widespread adoption across various fields for signal processing. Notably, significant advancements have been made in analyzing biological signals, which can exhibit diverse characteristics and patterns. Asynchronous spike encoder based on neuromorphic memristors have been used to reproduce and predict EEG and electrocardiogram (ECG) recordings^428^ (Figure 8A). In neuroprosthetics applications, for instance, signals from the motor cortex and electromyogram (EMG) are captured, processed, and used to stimulate movement. A relevant example of this is the Neurochip, a system that records spiking activity from the primary motor cortex of monkeys and EMG signals from their forearm muscles. This technology has demonstrated the ability to restore motor function by using spinal cord stimulation to trigger movements.^429,430^ Neuromorphic interfaces are also used to generate locomotor-like activity through intraspinal microstimulation methods, aimed at promoting motor rehabilitation in patients with spinal injuries. In this process, the neuromorphic hardware interacts with neural circuits to analyze muscle activity and generate precise stimulation.^431^ Neural signals can also be processed using neuromorphic systems to model and classify pathological conditions. For example, a biohybrid system was developed to detect pathological signals in rats and restore memory function after a pharmacological blockade of the hippocampus.^431^ In the field of neural interfaces, neural signals have been identified and analyzed to classify LFP and multiunit activity in response to external stimuli in organoids. Notably, researchers observed distinct responses to the presence or absence of visual stimuli and achieved a bidirectional synaptic connection between the mouse brain and human organoids, a remarkable breakthrough in the realm of neuromorphic interfaces.^432^

**Figure 8:**
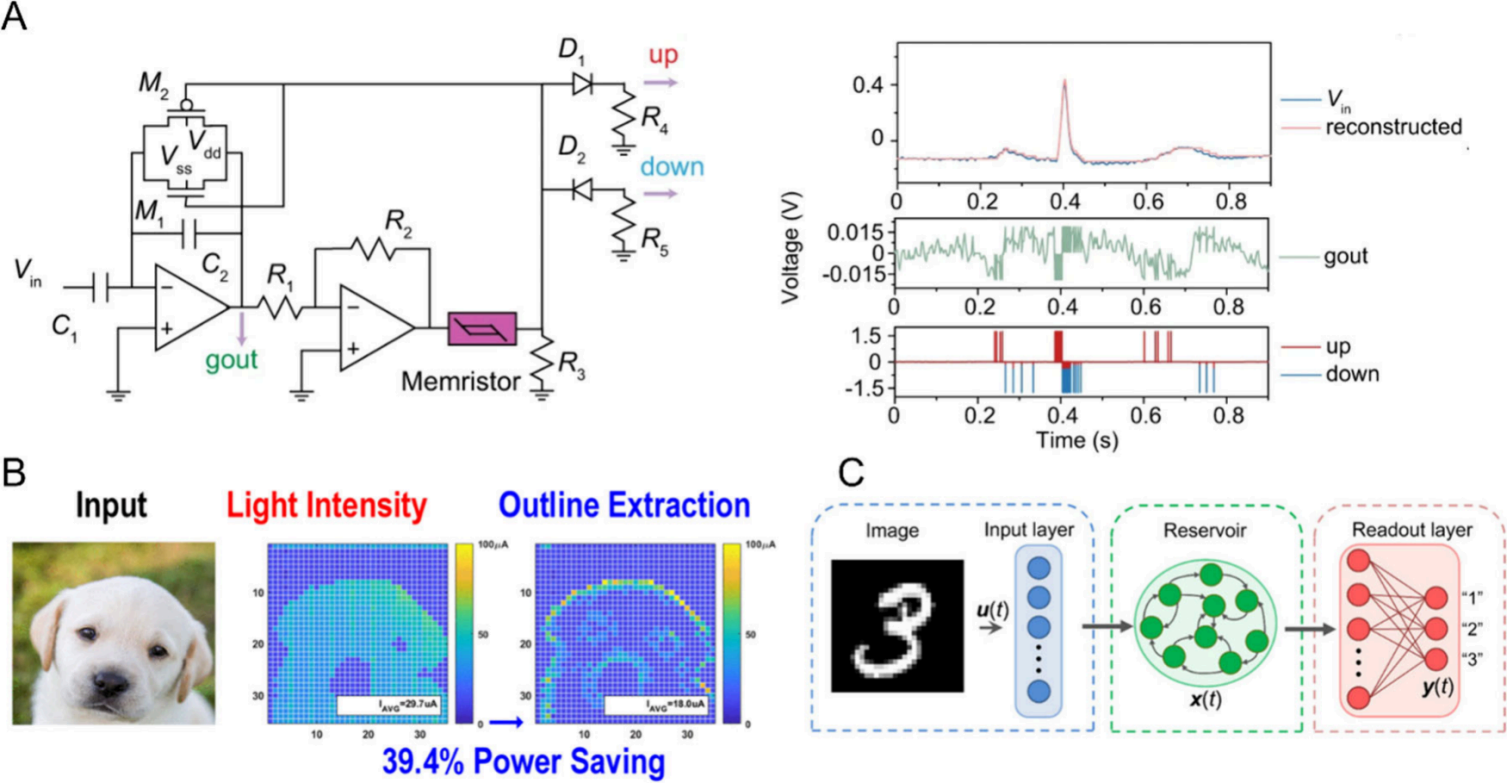
**Neuromorphic architectures for biosignal processing. A.** Memristors have been inserted in circuits in order to achieve the obtained sparse-spiking and on-demand encoding, in order to reconstruct neuromorphic signals. In this example they have been used for ECG heartbeat classification (adapted from reference 428). Copyright 2023, Springer Nature. Distributed under a Creative Commons CC BY License (CC BY 4.0). **B.** Neuromorphic devices have been adopted for the implementation of retinal-prosthesis examples. For instance, a localized temperature-regulation has allowed for an outline extraction with an overall power consumption that is lower than the physiological one (adapted from reference 433). Copyright 2020, IEEE. Distributed under a Creative Commons CC BY License (CC BY 4.0). **C.** In other applications memristor arrays have been put in RC systems to achieve image recognition through a reservoir dynamics (adapted from reference 434). Copyright 2023, Wiley-VCH GmbH. Distributed under a Creative Commons CC BY License (CC BY 4.0).

In a different approach, a system-on-chip was proposed, not yet validated *in vivo*, that integrates neuromorphic processing to optimize power consumption and stimulation precision. The device consists of 1,225 interconnected pixels, each made up of a photodiode paired with a neuromorphic image processor, enabling decentralized processing. This design aims to achieve power consumption levels comparable to those of the biological retina^433^ (Figure 8B). In a different application, memristors arrays have been put in connection with a culture of neurons to exploit the dynamics of the reservoir to complete tasks of image recognition^434^ (Figure 8C).

#### 4.4.2 *In Vitro* Platforms

Several neuromorphic solutions have emerged over the past decade for direct interfacing with biological systems, including bioinspired neural networks used for processing, computing, and classification tasks. For instance, metal-oxide memristors have been employed to record neural activity from retinal ganglion cells when connected to MEAs. These systems offer benefits such as efficient neural data recording, noise reduction, and scalability to larger neural networks, enabling real-time encoding and compression of neuronal spike activity.^435^ However, these inorganic memristors cannot be classified as neuromorphic or neurohybrid devices because their signal processing is separate from the neuron interfaces, and they lack the ability to exhibit synaptic plasticity. Biomimetic SNNs, on the other hand, can operate in real time, delivering biologically realistic stimulations to living cells, such as *in vitro* neurons and cerebral organoids, with validation through calcium imaging. Depending on the application, these systems can provide either spike-time-based or waveform-based stimulation.^436^ In a different approach, OMDs have been used to connect two cortical neurons, creating artificial synapses to simulate natural excitatory signals (Figure 9A). While OMDs mimic certain features of natural synapses, such as activity-dependent coupling and spike-timing properties, they still fall short in fully replicating key synaptic behaviors, including short- and long-term plasticity, which are essential for learning processes.^437^ Additionally, the communication between the neurons in these systems is unidirectional, which is not biologically accurate, and scaling to more complex biological networks remains a challenge. This limitation was addressed in a study involving a three-neuron brain-silicon network, where bidirectional communication between rat hippocampal neurons and silicon-based artificial neurons was implemented. In this setup, memristors emulated synaptic plasticity, including both LTP and depression, allowing for dynamic adjustments in connection strength based on neuronal firing rates^438^ (Figure 9B). The bidirectional communication pathways in this system make it adaptable for closed-loop neuroprosthetics and real-time adaptive responses in neurohybrid systems. However, the absence of organic materials affects its biocompatibility and the efficiency of information transmission. Another example of bidirectional communication is the use of a transparent organic transistor, which enabled both stimulation and recording of primary neurons, allowing for membrane hyperpolarization and depolarization. This device achieved a higher SNR compared to MEAs on the same neuronal preparation.^439^ However, in these examples, the transistors exhibit volatile memory, meaning they cannot reproduce plasticity mechanisms necessary for neuromorphic computing capabilities. Organic electrochemical transistors, which convert ionic signals to electronic information, have demonstrated the potential for biohybrid synapses. These devices showed nonvolatile memory when exposed to dopamine released from PC-12 cells, which was modulated by pulse-train voltage oxidation, mimicking synaptic behavior^398^ (Figure 9C). This principle could also be applied to other electroactive species released at the synaptic cleft, such as serotonin and ascorbic acid.^397^ While the system lacks bidirectionality, it successfully emulates synaptic plasticity. Another example of neurotransmitter-based communication between PC-12 cells and transistors involves a supercapacitive graphene nanowall gate electrode. The charging and discharging of the device are triggered by acetylcholine released from biological cells. When integrated into a Y-shaped “OR” gate circuit, this device mimics the integrate-and-fire model for spike generation and reproduces the paired pulse facilitation mechanism in response to neurotransmitter signals.^440^

**Figure 9:**
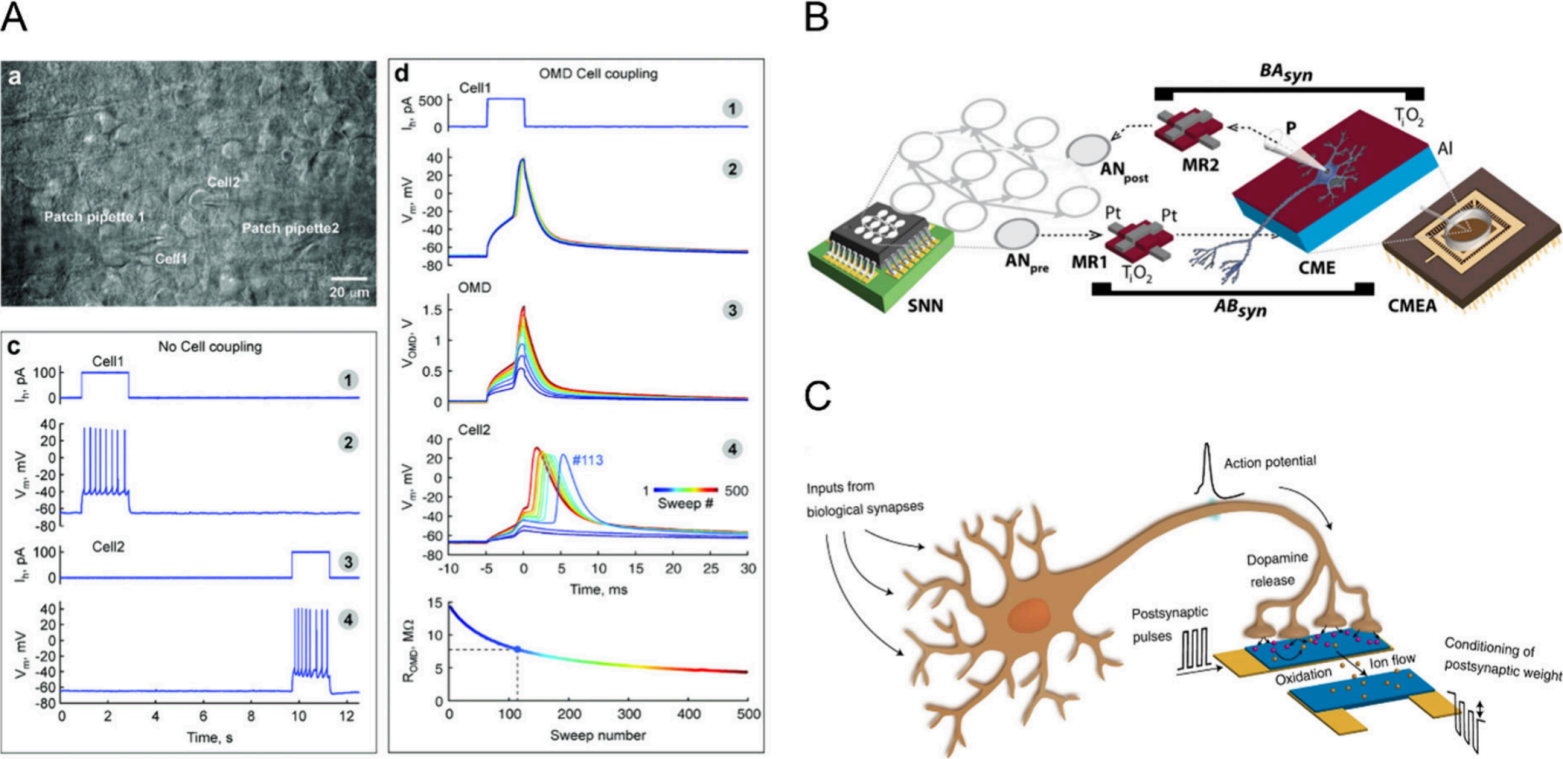
**In vitro neuromorphic applications. A**. To achieve a communication between biological neurons and a neuromorphic system, several strategies have been investigated. For example, organic memristive devices were connected to two cortical neurons, through a patch-clamp technique to simulate natural excitatory activity and allow for their coupling (adapted with permission from reference 437). Copyright 2018, Wiley-VCH GmbH. **B.** In another example, silicon memristors were connected to two neurons, showing bidirectional communication (adapted from reference 438). Copyright 2020, Springer Nature. Distributed under a Creative Commons CC BY License (CC BY 4.0). **C**. The direct interfacing between the biological cells and the OECT has allowed mimicking of synaptic plasticity driven by the neurotransmitter released from the culture in the biohybrid synapse (adapted from reference 398). Copyright 2020, the Authors, under exclusive license to Springer Nature Limited.

Optoelectronic stimulation presents another promising method for neuron interaction. In one example, a retina-inspired optoelectronic synapse using QDs was used for neuromorphic stimulation of hippocampal neurons. While the TiO_2_ material used in this setup is biocompatible, there have been concerns about potential toxicity.^441^

In the Brainoware application, brain organoids were interfaced with high-density MEAs to create a neuromorphic hardware system capable of adaptive reservoir computing. This system enables complex computing tasks, such as speech recognition, and supports unsupervised learning mechanisms.^442^ However, challenges remain in downscaling, maintaining organoid viability, and interfacing between organoids and hardware.

#### 4.4.3 *In Vivo* Platforms

Several challenges must be addressed when moving from *in vitro* to *in vivo* experiments. The first consideration is ethical: devices implanted in living organisms must meet strict standards, particularly minimizing inflammatory responses and ensuring stability and longevity. Extensive research is necessary before new materials can be transitioned from *in vitro* to *in vivo* use, as living tissues are significantly more complex and harder to control than *in vitro* cultures.^443^ Furthermore, issues such as device stability, longevity, and minimizing inflammatory responses when implanted *in vivo* must be fully addressed.^437,444^ In the field of technologies for bidirectional recording and stimulation of living tissue, there are applications such as interfaces for motor interaction with the environment, sensory prostheses to restore biological senses, and systems designed to replace damaged brain circuitry. Neuromorphic interfaces with neural tissue can be classified into three main categories: perception, control, and cognitive interfaces.^124,392^

In prosthetics, sensorimotor integration is essential for both biological systems and emerging robotic technologies. It involves coordinating sensory input with motor output, a process fundamental to exploring and adapting to the environment.^445^ Sensorimotor integration covers a range of biological processes, from simple reflexes to complex voluntary movements, enabling learning and decision-making based on sensory information. Developing this capability in artificial systems has been challenging.^429,446–450^ Traditional robotic systems rely on rigid structures and computationally intensive algorithms, which limit their adaptability and efficiency. However, advancements in organic electronics, such as flexible artificial synapses and optoelectronic systems, offer promising solutions to these limitations. For instance, artificial pupil reflex systems can utilize real-time associative learning and spatiotemporal integration in neuromorphic systems.^445^

In this context, the concept of embodiment is relevant as it considers the physical interaction between a system and its environment.^446^ The structure and material properties of a system, such as skin, play a key role in its sensorimotor abilities. Human skin not only provides sensory data but also enhances interaction through its softness and texture, making tasks like grasping objects easier. In robotics, replicating this level of embodied intelligence with artificial skin has been challenging, as traditional materials like silicon lack the flexibility and sensory capabilities of human skin. Conducting polymers, which can be fabricated with flexibility, presents a potential solution. Prototypes of electronic skin made from flexible organic materials are showing promise, with the ability to process pressure stimuli from the motor cortex in live rats, perform multimodal perception, generate neuromorphic pulse-train signals, and enable closed-loop actuation^445^ (Figure 10A). Recent work on artificial nociceptors demonstrates how synaptic transistors can mimic pain perception by integrating sensory and memory functions within a single device, responding to temperature variations in a way similar to biological skin^451^ (Figure 10B). Some systems are now capable of connecting to prosthetics, allowing amputees to feel textures and distinguish between objects, mimicking the sensory responses of human skin. However, challenges such as scalability and fabrication variability still hinder the full realization of artificial skin.^445^ Stretchable elastic synaptic transistors have demonstrated their applicability in neurologically integrated soft systems.^452^ On the other hand, neurohybrid systems can be used to restore neurodegenerative diseases with an adaptive stimulation, in which the signal coming from two different parts of the brain can be processed with a memristive device^453^ (Figure 10C).

**Figure 10:**
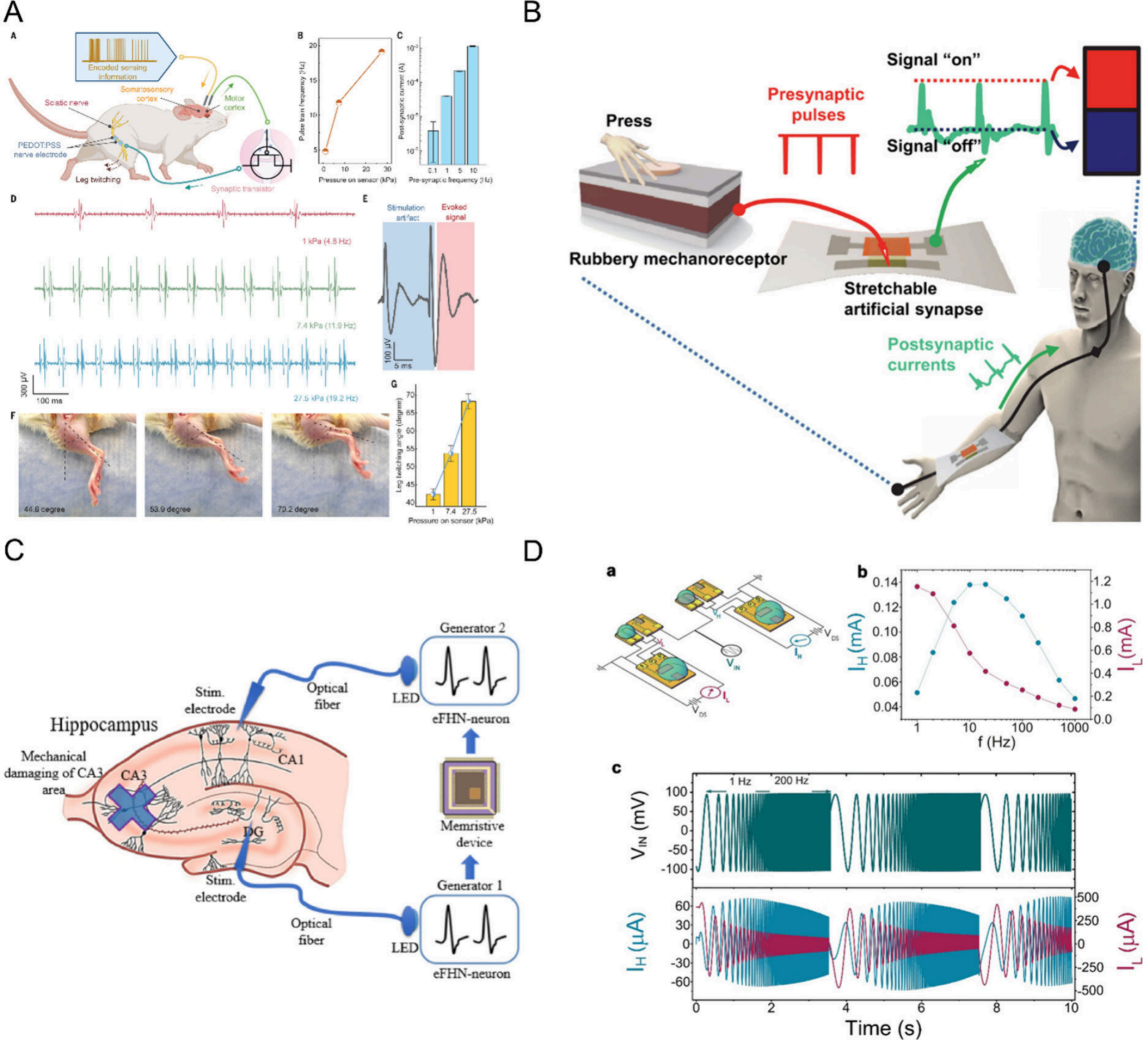
***In vivo* neuromorphic applications. A.** Neurormorphic devices can be applied for the integration of somatosensory integration to obtain a perception-action loop. An example is represented by the e-skin, in which a synaptic transistor is stimulated with signals from the somatosensory cortex and can cause leg-twiching in the sciatic nerve in the animal (adapted with permission from reference^445^). Copyright 2023, The American Association for the Advancement of Science. **B.** Sensory skin based synaptic transistors, in which the action potentials created by the on mechanoreceptor are transmitted to the artificial synapse (adapted with permission from reference 452). Copyright 2019, The American Association for the Advancement of Science. **C.** Neurohybrid systems have been exploited also for adaptive stimulation in the hyppocampus, through the use of memristors that can process and transmit the information from one area of stimulation to another (adapted with permission from reference 453). Copyright 2021, Elsevier Ltd. **D.** Biocompatible neuromorphic systems, based on organic electronic circuits have the advantage of performing local computation of electrocphysiological signals (adapted from reference 454). Copyright 2023, Wiley-VCH GmbH. Distributed under a Creative Commons CC BY License (CC BY 4.0).

To enable in situ processing of electrophysiological signals, EGOTs have been integrated into an organic circuit for real-time signal transduction, amplification, and sorting. This system leverages brain-like processing capabilities, aligning the biological time scales of neural signals with those of organic electronics (Figure 10D). A prerecorded *in vivo* SEP from a rat’s barrel cortex was used to benchmark the platform’s sorting and filtering abilities. The SEP signals, typically composed of alpha (5–15 Hz), beta (15–30 Hz), and gamma (30–150 Hz) frequency components, were processed in real time, demonstrating the system’s ability to isolate and amplify specific frequency bands of neural activity.^454^ In another application, a neural network model was trained to predict the deep calcium activity recorded by two-photon imaging while surface potentials were recorded by the graphene arrays. These *in vivo* experiments validated the ability of the platform to conduct multimodal neural recordings characterized by high spatiotemporal resolution.^213^

## 5 Ethics and Outlook

The development of neurohybrid interfaces, where artificial neuromorphic systems are integrated with biological neural circuits, presents profound ethical challenges that must be addressed as these technologies advance toward clinical application. As this review highlights, the technical complexity of creating devices capable of interfacing with living neural tissue, whether through SNNs, OECTs, or CPs, brings with it a unique set of ethical concerns grounded in the specific ways these devices interact with the nervous system.

One of the most pressing ethical issues arises from the ability of neurohybrid interfaces to potentially influence or alter neural activity. Devices designed to monitor and stimulate neuronal circuits, such as those employing closed-loop systems for treating epilepsy or neurodegenerative conditions, have the capacity to modify brain function in real time.^20^ This opens up concerns related to autonomy and cognitive liberty.^455^ For instance, there is uncertainty on how to ensure that these devices operate in ways that support the individual’s control over their own mental processes, a right and ability often referred to as ‘mental self-determination’.^456^ This is particularly relevant for closed-loop systems where neural stimulation is adjusted automatically based on neurofeedback. The technical aim of achieving seamless bidirectional communication between artificial and biological systems raises questions about the limits of human agency when devices have the power to modulate neural signals continuously. The challenge lies in balancing therapeutic efficacy, such as preventing seizures or mitigating Parkinsonian tremors, without undermining an individual’s sense of control over their own cognitive and motor functions.^457^

A significant emerging trend is the integration of advanced AI algorithms, particularly deep learning and reinforcement learning, into neurohybrid platforms to enable personalized neural modulation. These systems can detect and model individual neurophysiological signatures by analyzing electrophysiological recordings, such as LFPs or EEG data, in real time. Through continuous data acquisition and model updating, AI-enhanced closed-loop systems can dynamically adjust stimulation parameters (e.g., frequency, amplitude, spatial targeting) based on patient-specific biomarkers of pathological activity. For instance, convolutional neural networks and long short-term memory architectures have demonstrated success in predicting epileptic seizures from intracranial EEG signals with high sensitivity and specificity, enabling preemptive neurostimulation that could abort seizure onset. This convergence of adaptive AI with organic or neuromorphic electronics capable of bidirectional interfacing, such as OECTs or memristive synapses, heralds a shift toward individualized neuromodulation protocols rooted in the principles of precision medicine. These developments are also representative of the broader trend toward technological convergence, which envisions a fusion of biological, digital, and cognitive systems into cohesive, hybridized infrastructures.^458^

As neurohybrid interfaces evolve into increasingly autonomous and adaptive therapeutic systems, it becomes imperative to develop robust ethical oversight mechanisms, adaptive regulatory frameworks that can accommodate algorithmic evolution, and interdisciplinary collaborations spanning neuroscience, computer science, ethics, and law. These efforts are essential not only for realizing the clinical promise of AI-augmented neurotechnologies but also for safeguarding human agency and mental integrity in the context of deepening brain-machine symbiosis.

Ethical implications extend further when considering the data privacy issues inherent in neuromorphic platforms. Devices such as organic transistors, which can transduce both ionic and electrical signals, have the potential to capture a broad array of neural data. The precise recording of electrical activity in living neurons, combined with neuromorphic signal processing, enables these systems to gather information not only about motor commands but potentially about thoughts, emotions, and cognitive states.^459,460^ The sensitivity of these neural data demands rigorous data governance protocols to prevent unauthorized access or exploitation.^460^ With technologies that bridge the biological and digital domains, thereby referred to as converging technologies,^458^ questions about who owns this neural data and how it can be used, especially as it is processed through artificial platforms, are critical.^455^ Moreover, the technical flexibility of these devices, such as their ability to adapt to changing neural signals in real time, could be exploited for purposes beyond therapy, such as cognitive enhancement or surveillance, further introducing uncertainty into the ethical landscape.

A particularly urgent ethical challenge emerges from the research and development phase of neurohybrid interfaces. The iterative process of designing neuromorphic devices capable of mimicking synaptic plasticity and learning functions, such as in memristors or OECTs, requires extensive testing in both animal models and human trials. Here, traditional concerns about the safety, efficacy, and long-term impacts of neural implants intersect with the emerging complexities of adaptive and learning systems. For example, neuromorphic devices that emulate brain-like learning raise questions about how to assess safety when the system’s behavior can evolve over time. Testing devices that not only interact with but also “learn” from the neural environment introduces ethical uncertainties about predictability and control, as it may be difficult to anticipate how these systems will behave after extended use.

The potential for neurohybrid interfaces to enhance human capacities also brings into focus the ethical boundaries between therapeutic use and human enhancement. Neuromorphic technologies, designed to emulate the brain’s efficiency and adaptability, could be employed not only to restore lost functions but to augment cognitive abilities, memory, or sensory perception. These developments challenge existing ethical frameworks that distinguish between medical treatment and enhancement, an issue that appears remarkably polarizing in the public.^461^ Moreover, the risk of creating disparities between those who can access these advanced neurotechnologies and those who cannot, it could exacerbate social inequalities, particularly if enhancements are marketed as consumer products. These inequities become even more pronounced when considering the cost and accessibility of the materials and technologies involved in producing neurohybrid systems, such as the use of sophisticated organic polymers or cutting-edge neuromorphic chips like Intel’s Loihi.

Research ethics are particularly critical as the field moves toward integrating more complex closed-loop systems in clinical trials. The use of adaptive technologies, which respond dynamically to changing neural environments, presents challenges for obtaining informed consent. Patients may consent to a device with specific therapeutic parameters, but as the system adapts and modifies its behavior, ensuring ongoing informed consent becomes problematic. In such cases, both patients and researchers need to be aware of the evolving nature of technology and the potential for unforeseen effects on neural activity. Informed consent becomes particularly fraught when devices evolve over time as they may deviate from the initially consented to risk profiles. This is particularly relevant for neurohybrid interfaces relying on machine learning. This raises the need for ’dynamic consent’ models, where patients can regularly revisit and adjust their consent based on device performance and emerging effects. We recommend deployers of AI-reliant neurohybrid interfaces to prioritize dynamic vs static consent models, ideally based on interactive personalized interfaces that allow participants to engage as. As they choose and to alter their consent choices in real time.^462,463^

There is also the broader question of how to ethically design experiments where the line between human and machine agency becomes increasingly blurred, particularly when studying adaptive interfaces that could exert autonomous influence over brain function.^457,464^ In such systems, decision-making processes are partially delegated to algorithms capable of learning from and modifying neural activity in real time. This raises complex ethical issues related to accountability, transparency, and user consent. For example, if an AI-driven interface modifies neural stimulation patterns based on internal optimization criteria rather than explicit human input, it becomes difficult to determine whether resulting changes in cognition or behavior are attributable to the subject or to the system. It was argued that experimental protocols must therefore incorporate safeguards such as algorithmic explainability, thresholds for autonomous intervention, and mechanisms for user override.^465^ Furthermore, researchers must carefully assess the psychological and phenomenological impact of interacting with systems that may not only respond to but also shape one’s cognitive and emotional states. These considerations are critical for preserving human agency and ensuring that participants are not subjected to unintended forms of behavioral or emotional manipulation.

In the outlook for neurohybrid interfaces, interdisciplinary collaboration will be crucial for addressing these ethical concerns. The development of neuromorphic technologies requires not only technical expertise in bioengineering, materials science, and computational neuroscience but also engagement with neuroethicists, legal scholars, data security experts and regulatory bodies. Ethical foresight must accompany technical innovation to ensure that the promise of neurohybrid interfaces for treating neurological disorders is realized without compromising individual rights, privacy, or societal well-being. Similarly, legal compliance is necessary.

However, achieving legal compliance may be challenging as the legal landscape surrounding neurohybrid interfaces is evolving with many of these technologies challenging existing regulatory frameworks in various jurisdictions. In the United States, the FDA is the primary body responsible for regulating medical devices, including neural interfaces. Devices such as closed-loop systems, BCIs and BMIs would need to undergo premarket approval or clearance under FDA’s IDE process, which governs clinical trials for high-risk devices. However, neuromorphic devices, particularly those that involve deep learning, raise questions about whether the current regulatory criteria, which focus on static risk assessments, are equipped to evaluate systems that adapt over time. The FDA’s recent Digital Health Innovation Action Plan recognizes this gap, as it outlines efforts to develop frameworks for regulating adaptive and evolving AI-based medical devices, which would be crucial for neuromorphic interfaces.

In Europe, the regulation of neurohybrid systems falls under the MDR, which came into effect in 2021. Under the MDR, neural implants and other neurohybrid technologies would be classified as high-risk Class III devices, subject to strict premarket evaluation for safety and efficacy. However, as with the FDA, the current European regulations are focused on devices with predictable and static behaviors, creating uncertainties around the regulatory pathways for dynamic learning systems. The GDPR also plays a critical role in governing the use of neural data in Europe. The GDPR mandates stringent protections for personal data, which includes the neural data captured by neurohybrid interfaces.^460^ While the GDPR provides a strong foundation for data protection, its applicability to neurohybrid interfaces and adaptive neurotechnologies in general remains ambiguous. For instance, the regulation does not yet clearly delineate how data minimization principles apply to systems that continuously collect and analyze neural data. Similarly, the applicability of the right to explanation is questionable in the context of neurohybrid interfaces as explainable AI (XAI) models for neuromorphic systems remain rare.^466,458,459^ Beyond Europe, intergovernmental efforts such as the OECD Principles on AI and the UNESCO Recommendation on the Ethics of Artificial Intelligence call for anticipatory governance frameworks that address data misuse and algorithmic opacity. These guidelines emphasize the need for transparency, human oversight, and accountability, which are particularly relevant for neurohybrid interfaces as they increasingly integrate AI-driven functionalities. However, while these global initiatives provide valuable normative anchors, their practical implementation and enforceability remain significant challenges. Many of these frameworks are nonbinding and rely on voluntary compliance by states or organizations, which limits their ability to ensure consistent application across jurisdictions. Moreover, the technical complexity and opacity of AI-enabled neurotechnologies often outpace the regulatory capacities of existing institutions, resulting in enforcement gaps and regulatory uncertainty. Addressing these limitations will require the development of concrete enforcement mechanisms, such as auditability requirements, regulatory sandboxes for experimental oversight, and international cooperation on standards and accountability practices tailored to convergent neurotechnologies.

One additional concern is liability in the event of device malfunction or unintended changes in neural behavior. As neurohybrid systems like OECTs or memristors can adapt their signal processing, questions arise over who holds responsibility when things go wrong: the manufacturer, the healthcare provider, or the system itself? Consensus papers by multidisciplinary experts^460,467,468^ as well as institutional reports (EU Parliament 2024,^440^ Council of Europe 2024 ^469^) have concluded that current legal frameworks are ill-equipped to handle the complexity of adaptive systems, and new regulations are needed to address liability in these contexts.

Finally, the dual-use potential of neurohybrid systems, where the same technologies used for therapy could be adapted for cognitive manipulation, interrogation, or performance enhancement, demands urgent ethical scrutiny. As with other AI-enabled biomedical technologies, the risks of weaponization or coercive use must be considered, particularly in settings such as military, correctional institutions, or high-performance professions. For instance, adaptive neurotechnologies capable of modulating stress resilience or alertness could, in theory, be deployed to optimize combat performance or enhance belligerance, raising serious concerns about consent, autonomy, and abuse. While outright prohibitions of military application are likely ineffective,^470,471^ safeguards will be needed not only with regard to technical constraints on usage but also export controls, and review processes that explicitly account for dual-use scenarios. Embedding these protections into both design and deployment pathways is essential to prevent misuse and to ensure that neurohybrid innovations remain aligned with human rights and humanitarian norms.

## 6 Conclusions

Implementing neuromorphic technologies for long-term interfacing with the neuronal system presents numerous challenges. Key considerations include accurately emulating neuronal functions while ensuring biocompatibility, preventing signal mismatches, and integrating both processing and sensing capabilities into a single device. Neuromorphic devices offer the potential to combine computation and sensing directly within biological environments. This is especially important for neurointerfacing, where chemical signals often precede electrical ones in biological processes.

Achieving energy-efficient, real-time neurointerfacing requires devices to function with parameters similar to the biological counterpart, such as low operating voltages and minimal power consumption. However, current artificial synapses still fall short compared to inorganic alternatives in terms of endurance and speed. Advanced materials like soft or organic semiconductors are increasingly preferred for their biocompatibility and mechanical properties that closely match biological tissues, reducing inflammatory responses and promoting long-term integration between electronics and neurons at cellular and tissue level.

Wearable and implantable devices, essential for continuous *in vivo* monitoring, benefit from the energy efficiency and adaptability of neuromorphic platforms. These devices can better interpret biological signals, even in noisy conditions, by acting as thresholded integrators. However, direct biointerfacing exposes electronics to harsh environments, necessitating the use of durable materials like conducting polymers that can withstand extended exposure to water and ions.

To achieve seamless biointerfacing, further advancements in material reliability, durability, and the miniaturization of operational voltages are needed. Bidirectional communication between electronic and biological systems can be enabled through multimodal interfaces that replicate both electrical and biochemical processes, such as synaptic plasticity. This will support more efficient edge computing and enable real-time interactions between neural networks and electronic devices. In turn, self-adaptable neuronal interfaces should provide advanced multimodal sensing, on chip computing exploiting organic and inorganic interfaces and architectures with closed-loop endorsement to provide required targeted stimulation to support and restore lost neuronal functionalities and efficient prosthetic interfacing.

## Abbreviations

TCF: 2-dicyanomethylen-3-cyano 4,5,5-trimethyl-2,5-dihydrofuran
ADCs: Analog-to-digital converters
AI: Artificial Intelligence
ANNs: Artificial neural networks
bPAC: Beggiatoa photoactivated adenylyl cyclase
BNNs: Biological neural networks
BDD: Boron-doped diamond
BCI: Brain-computer interface
BNI: Brain–Neuromorphics Interface
BHJ: Bulk heterojunction
CdSe: Cadmium selenide
CdS: Cadmium sulfide
cAMP: Cyclic adenosine monophosphate
ChR2: Channelrhodopsin 2
CMOS: Complementary metal–oxide–semiconductor
CBRAM: Conductive bridging random-access memory
CP: Conductive polymer
cGMP: Cyclic Guanosine Monophosphate
DBS: Deep brain stimulation
DACs: Digital-to-analog converters
DSP: Digital signal processor
D-A: Donor–acceptor
DBA: Donor–bridge–acceptor
ECG: Electrocardiogram
ECoG: Electrocorticography
EEG: Electroencephalography
EDL: Electrical double layer
EGOT: Electrolyte-Gated Organic Transistor
EGOFET: Electrolyte-Gated Organic Field-Effect Transistor
EPSP: Excitatory postsynaptic potential
FSCV: Fast-scan cyclic voltammetry
FET: Field-Effect Transistor
FPGA: Field-programmable gate array
FBR: Foreign Body Response
FDA: Food Drug Administration
GABA: Gamma-aminobutyric acid
GDPR: General Data Protection Regulation
GOQDs: Graphene oxide quantum dots
GPUs: Graphical processing units
h-BN: Hexagonal boron nitride
IPSP: Inhibitory postsynaptic potential
IGZO: Indium–gallium-zinc-oxide
ITO: Indium Tin Oxide
IGTs: Internal ion-gated organic electrochemical transistors
iBCI: Invasive brain-computer interface
IDE: Investigational Device Exemption
IrOx: Iridium oxide
LFPs: Local field potentials
LTD: Long-term depression
LTP: Long-term potentiation
MDR: Medical Devices Regulation
μECoG: Microelectrocortigography
MEA: Microelectrode array
mGluR: Metabotropic glutamate receptor
MOSFET: Metal-Oxide-Semiconductor Field-Effect Transistor
MoS_2_: Molybdenum disulfide
NIR: Near Infrared
NNN: Neurohybrid neuronal networks
NMDAR: *N*-Methyl-d-Aspartate receptor
OECN: Organic electrochemical neuron
OECT: Organic electrochemical transistor
OEPC: Organic Electrolytic Photocapacitor
OEIP: Organic electronic ion pump
OMD: Organic memristive device
OMIEC: Organic mixed ionic-electronic conductor
PEDOT: Poly­(3,4-ethylenedioxythiophene)
PEDOT:PSS: Poly­(3,4-ethylenedioxythiophene):polystyrenesulfonate
PCM: Phase change material
P3HT: Phytochrome interacting factor (PIF)­Poly­(3-hexylthiophene-2,5-diyl)
PTB7-Th: Poly­([2,6′-4,8-di­(5-ethylhexylthienyl)­benzo­[1,2-b;3,3-b]­dithiophene]­{3-fluoro-2­[(2-ethylhexyl)­carbonyl]­thieno­[3,4-*b*]­thiophenediyl})
PCPDTBT: Poly­[2,6-(4,4-bis­(2-ethylhexyl)-4H-cyclopenta­[2,1-b;3,4-b′]­dithiophene)-*alt*-4,7­(2,1,3-benzothiadiazole)]
PEI: Poly­(ethylenimine)
PDMS: Polydimethylsiloxane
QD: Quantum dot
rGO: Reduced Graphene Oxide
RRAM: Resistive random-access memory
ROS: Reactive oxygen species
SELEX: Systematic evolution of ligands by the exponential enrichment
SPI: Serial Peripheral Interface
SNR: Signal-to-noise ratio
SEP: Somatosensory evoked potential
SNNs: Spiking neural networks
STDP: Spike-timing-dependent plasticity
TMDs: Transition metal dichalcogenides
TPA: Triphenylamine
USB: Universal Serial Bus
ZHO: Zr_0.5_Hf_0.5_O_2_

## References

[1] Marković D., Mizrahi A., Querlioz D., Grollier J. (2020). Physics for Neuromorphic
Computing. Nat. Rev. Phys..

[2] Zhang, J. Basic Neural Units of the Brain: Neurons, Synapses and Action Potential. *arXiv* May 30, 2019. http://arxiv.org/abs/1906.01703 (accessed 2024–11–01).

[3] Sporns O. (2011). The Human
Connectome: A Complex Network. Ann. N.Y. Acad.
Sci..

[4] Denève S., Alemi A., Bourdoukan R. (2017). The Brain
as an Efficient and Robust
Adaptive Learner. Neuron.

[5] Luan L., Yin R., Zhu H., Xie C. (2023). Emerging Penetrating Neural Electrodes:
In Pursuit of Large Scale and Longevity. Annu.
Rev. Biomed. Eng..

[6] Przedborski S., Vila M., Jackson-Lewis V. (2003). Series Introduction:
Neurodegeneration:
What Is It and Where Are We?. J. Clin. Invest..

[7] Reeve A., Simcox E., Turnbull D. (2014). Ageing and Parkinson’s Disease:
Why Is Advancing Age the Biggest Risk Factor?. Ageing Res. Rev..

[8] Reitz C., Brayne C., Mayeux R. (2011). Epidemiology of Alzheimer
Disease. Nat. Rev. Neurol..

[9] Hofmann U. G., Stieglitz T. (2024). Why Some BCI
Should Still Be Called BMI. Nat. Commun..

[10] Zhang J., Li J., Huang Z., Huang D., Yu H., Li Z. (2023). Recent Progress
in Wearable Brain-Computer Interface (BCI) Devices Based on Electroencephalogram
(EEG) for Medical Applications: A Review. Health
Data Sci..

[11] Hochberg L. R., Serruya M. D., Friehs G. M., Mukand J. A., Saleh M., Caplan A. H., Branner A., Chen D., Penn R. D., Donoghue J. P. (2006). Neuronal Ensemble
Control of Prosthetic Devices by
a Human with Tetraplegia. Nature.

[12] Campbell P. K., Jones K. E., Huber R. J., Horch K. W., Normann R. A. (1991). A Silicon-Based,
Three-Dimensional Neural Interface: Manufacturing Processes for an
Intracortical Electrode Array. IEEE Trans. Biomed.
Eng..

[13] Wilson B. C., Jermyn M., Leblond F. (2018). Challenges
and Opportunities in Clinical
Translation of Biomedical Optical Spectroscopy and Imaging. J. Biomed. Opt..

[14] Schuman C. D., Kulkarni S. R., Parsa M., Mitchell J. P., Date P., Kay B. (2022). Opportunities for Neuromorphic Computing
Algorithms and Applications. Nat. Comput. Sci..

[15] Li H., Wang J., Fang Y. (2023). Recent Developments in Multifunctional
Neural Probes for Simultaneous Neural Recording and Modulation. Microsyst. Nanoeng..

[16] Wang Y., Liu S., Wang H., Zhao Y., Zhang X.-D. (2022). Neuron Devices:
Emerging Prospects in Neural Interfaces and Recognition. Microsyst. Nanoeng..

[17] Turing A. M. I. (1950). -COMPUTING
MACHINERY AND INTELLIGENCE. Mind.

[18] Vidal J. J. (1973). Toward
Direct Brain-Computer Communication. Annu. Rev.
Biophys. Bioeng..

[19] Wan C., Pei M., Shi K., Cui H., Long H., Qiao L., Xing Q., Wan Q. (2024). Toward a Brain-Neuromorphics
Interface. Adv. Mater..

[20] Valeriani D., Santoro F., Ienca M. (2022). The Present and Future of Neural
Interfaces. Front. Neurorobotics.

[21] Xiong H., Chu C., Fan L., Song M., Zhang J., Ma Y., Zheng R., Zhang J., Yang Z., Jiang T. (2023). The Digital
Twin Brain: A Bridge between Biological and Artificial Intelligence. Intell. Comput..

[22] Wang H. E., Triebkorn P., Breyton M., Dollomaja B., Lemarechal J.-D., Petkoski S., Sorrentino P., Depannemaecker D., Hashemi M., Jirsa V. K. (2024). Virtual Brain Twins:
From Basic Neuroscience to Clinical Use. Natl.
Sci. Rev..

[23] Lin Z., Zhang C., Zeng Y., Tong L., Yan B. (2018). A Novel P300
BCI Speller Based on the Triple RSVP Paradigm. Sci. Rep..

[24] Jung I.-H., Chang K. W., Park S. H., Chang W. S., Jung H. H., Chang J. W. (2022). Complications After Deep Brain Stimulation: A 21-Year
Experience in 426 Patients. Front. Aging Neurosci..

[25] Bronte-Stewart, H. M. ; Petrucci, M. N. ; O’Day, J. J. ; Afzal, M. F. ; Parker, J. E. ; Kehnemouyi, Y. M. ; Wilkins, K. B. ; Orthlieb, G. C. ; Hoffman, S. L. Perspective: Evolution of Control Variables and Policies for Closed-Loop Deep Brain Stimulation for Parkinson’s Disease Using Bidirectional Deep-Brain-Computer Interfaces. Front. Hum. Neurosci. 2020, 14, 10.3389/fnhum.2020.00353. PMC7489234 33061899

[26] Renard Y., Lotte F., Gibert G., Congedo M., Maby E., Delannoy V., Bertrand O., Lécuyer A. (2010). OpenViBE:
An Open-Source Software Platform to Design, Test, and Use Brain-Computer
Interfaces in Real and Virtual Environments. Presence Teleoperators Virtual Environ..

[27] Stieglitz T., Rubehn B., Henle C., Kisban S., Herwik S., Ruther P., Schuettler M. (2009). Brain-Computer
Interfaces: An Overview
of the Hardware to Record Neural Signals from the Cortex. Prog. Brain Res..

[28] Maynard E. M., Nordhausen C. T., Normann R. A. (1997). The Utah Intracortical
Electrode
Array: A Recording Structure for Potential Brain-Computer Interfaces. Electroencephalogr. Clin. Neurophysiol..

[29] Rakhmatulin I., Parfenov A., Traylor Z., Nam C. S., Lebedev M. (2021). Low-Cost Brain
Computer Interface for Everyday Use. Exp. Brain
Res..

[30] Siegelmann H. T. (2003). [No Title
Found]. Minds Mach..

[31] Mehonic A., Sebastian A., Rajendran B., Simeone O., Vasilaki E., Kenyon A. J. (2020). Memristors—From
In-Memory Computing, Deep Learning
Acceleration, and Spiking Neural Networks to the Future of Neuromorphic
and Bio-Inspired Computing. Adv. Intell. Syst..

[32] Merolla P. A., Arthur J. V., Alvarez-Icaza R., Cassidy A. S., Sawada J., Akopyan F., Jackson B. L., Imam N., Guo C., Nakamura Y., Brezzo B., Vo I., Esser S. K., Appuswamy R., Taba B., Amir A., Flickner M. D., Risk W. P., Manohar R., Modha D. S. (2014). A Million
Spiking-Neuron
Integrated Circuit with a Scalable Communication Network and Interface. Science.

[33] Kandel, E. R. ; Schwartz, J. H. ; Jessell, T. M. ; Siegelbaum, S. A. ; Hudspeth, A. J. Principles of Neural Science, Fifth ed.; McGraw Hill Professional, 2012.

[34] Oláh S., Füle M., Komlósi G., Varga C., Báldi R., Barzó P., Tamás G. (2009). Regulation of Cortical Microcircuits
by Unitary GABA-Mediated Volume Transmission. Nature.

[35] Chen T.-S., Huang T.-H., Lai M.-C., Huang C.-W. (2023). The Role of Glutamate
Receptors in Epilepsy. Biomedicines.

[36] Drachman D. A. (2005). Do We Have
Brain to Spare?. Neurology.

[37] Zador A. M. (2000). The Basic
Unit of Computation. Nat. Neurosci..

[38] Kennedy M.
B. (2016). Synaptic
Signaling in Learning and Memory. Cold Spring
Harb. Perspect. Biol..

[39] Jabeen S., Thirumalai V. (2018). The Interplay between Electrical
and Chemical Synaptogenesis. J. Neurophysiol..

[40] Südhof T. C., Malenka R. C. (2008). Understanding Synapses: Past, Present, and Future. Neuron.

[41] Feldmann J., Youngblood N., Wright C. D., Bhaskaran H., Pernice W. H. P. (2019). All-Optical Spiking Neurosynaptic Networks with Self-Learning
Capabilities. Nature.

[42] Südhof T. C. (2021). The Cell
Biology of Synapse Formation. J. Cell Biol..

[43] Wang, B. ; Dudko, O. K. A Theory of Synaptic Transmission. eLife 2021, 10, e73585. 10.7554/eLife.73585. 34970965 PMC8776255

[44] Morris R. G. M. D.O. (1999). Hebb: *The Organization of
Behavior*, Wiley: New York;
1949. Brain Res. Bull..

[45] Bi G., Poo M. (1998). Synaptic Modifications
in Cultured Hippocampal Neurons: Dependence
on Spike Timing, Synaptic Strength, and Postsynaptic Cell Type. J. Neurosci..

[46] Keller E. A., Borghese C. M., Carrer H. F., Ramirez O. A. (1992). The Learning Capacity
of High or Low Performance Rats Is Related to the Hippocampus NMDA
Receptors. Brain Res..

[47] Stecher J., Müller W. E., Hoyer S. (1997). Learning Abilities Depend on NMDA-Receptor
Density in Hippocampus in Adult Rats. J. Neural
Transm..

[48] Wang H., Peng R.-Y. (2016). Basic Roles of Key Molecules Connected with NMDAR Signaling
Pathway on Regulating Learning and Memory and Synaptic Plasticity. Mil. Med. Res..

[49] Chetkovich D. M., Gray R., Johnston D., Sweatt J. D. (1991). N-Methyl-D-Aspartate
Receptor Activation Increases cAMP Levels and Voltage-Gated Ca2+ Channel
Activity in Area CA1 of Hippocampus. Proc. Natl.
Acad. Sci. U. S. A..

[50] Lisman J. (1989). A Mechanism
for the Hebb and the Anti-Hebb Processes Underlying Learning and Memory. Proc. Natl. Acad. Sci. U. S. A..

[51] Cummings J. A., Mulkey R. M., Nicoll R. A., Malenka R. C. (1996). Ca2+ Signaling Requirements
for Long-Term Depression in the Hippocampus. Neuron.

[52] Collingridge G. L., Kehl S. J., McLennan H. (1983). Excitatory Amino Acids
in Synaptic
Transmission in the Schaffer Collateral-Commissural Pathway of the
Rat Hippocampus. J. Physiol..

[53] Kauer J. A., Malenka R. C., Nicoll R. A. (1988). A Persistent
Postsynaptic Modification
Mediates Long-Term Potentiation in the Hippocampus. Neuron.

[54] Malenka R. C., Kauer J. A., Perkel D. J., Mauk M. D., Kelly P. T., Nicoll R. A., Waxham M. N. (1989). An Essential Role
for Postsynaptic
Calmodulin and Protein Kinase Activity in Long-Term Potentiation. Nature.

[55] Jones M. W., Errington M. L., French P. J., Fine A., Bliss T. V. P., Garel S., Charnay P., Bozon B., Laroche S., Davis S. (2001). A Requirement
for the Immediate Early Gene Zif268 in the Expression
of Late LTP and Long-Term Memories. Nat. Neurosci..

[56] Krug M., Lössner B., Ott T. (1984). Anisomycin Blocks the Late Phase
of Long-Term Potentiation in the Dentate Gyrus of Freely Moving Rats. Brain Res. Bull..

[57] Nguyen P. V., Abel T., Kandel E. R. (1994). Requirement
of a Critical Period
of Transcription for Induction of a Late Phase of LTP. Science.

[58] Meldrum B. S. (2000). Glutamate
as a Neurotransmitter in the Brain: Review of Physiology and Pathology. J. Nutr..

[59] Lee S.-H., Dan Y. (2012). Neuromodulation of
Brain States. Neuron.

[60] Zoli M., Jansson A., Syková E., Agnati L. F., Fuxe K. (1999). Volume Transmission
in the CNS and Its Relevance for Neuropsychopharmacology. Trends Pharmacol. Sci..

[61] Umbriaco D., Garcia S., Beaulieu C., Descarries L. (1995). Relational
Features of Acetylcholine, Noradrenaline, Serotonin and GABA Axon
Terminals in the Stratum Radiatum of Adult Rat Hippocampus (CA1). Hippocampus.

[62] Milusheva E., Baranyi M., Zelles T., Mike Á., Vizi E. S. (1994). Release
of Acetylcholine and Noradrenaline from the Cholinergic and Adrenergic
Afferents in Rat Hippocampal CA1, CA3 and Dentate Gyrus Regions. Eur. J. Neurosci..

[63] Marchi M., Paudice P., Raiteri M. (1981). Autoregulation
of Acetylcholine Release
in Isolated Hippocampal Nerve Endings. Eur.
J. Pharmacol..

[64] Scanziani M., Gahwiler B., Thompson S. (1993). Presynaptic Inhibition
of Excitatory
Synaptic Transmission Mediated by Alpha Adrenergic Receptors in Area
CA3 of the Rat Hippocampus in Vitro. J. Neurosci..

[65] Hasselmo M., Schnell E. (1994). Laminar Selectivity of the Cholinergic Suppression
of Synaptic Transmission in Rat Hippocampal Region CA1: Computational
Modeling and Brain Slice Physiology. J. Neurosci..

[66] Behrends J. C., ten Bruggencate G. (1993). Cholinergic Modulation of Synaptic Inhibition in the
Guinea Pig Hippocampus in Vitro: Excitation of GABAergic Interneurons
and Inhibition of GABA-Release. J. Neurophysiol..

[67] Dharani, K. Chapter 2 - Physiology of the Neuron. In The Biology of Thought; Dharani, K. , Ed.; Academic Press: San Diego, 2015; pp 31–52. 10.1016/B978-0-12-800900-0.00002-6.

[68] Byrne, J. H. CHAPTER 16 - Postsynaptic Potentials and Synaptic Integration. In From Molecules to Networks; Byrne, J. H. ; Roberts, J. L. , Eds.; Academic Press: Burlington, 2004; pp 459–478. 10.1016/B978-012148660-0/50017-2.

[69] Platkiewicz J., Brette R. (2010). A Threshold Equation for Action Potential Initiation. PLoS Comput. Biol..

[70] Barnett M. W., Larkman P. M. (2007). The Action Potential. Pract.
Neurol..

[71] Ulbricht W. (2005). Sodium Channel
Inactivation: Molecular Determinants and Modulation. Physiol. Rev..

[72] Kandel, E. R. ; Schwartz, J. H. ; Jessell, T. M. ; Siegelbaum, S. ; Hudspeth, A. J. ; Mack, S. Principles of Neural Science; McGraw-Hill New York, 2000; Vol. 4.

[73] Hensch T. K. (2005). Critical
Period Plasticity in Local Cortical Circuits. Nat. Rev. Neurosci..

[74] Holtmaat A., Svoboda K. (2009). Experience-Dependent
Structural Synaptic Plasticity
in the Mammalian Brain. Nat. Rev. Neurosci..

[75] Dudai Y. (1996). Consolidation:
Fragility on the Road to the Engram. Neuron.

[76] McGaugh J. L. (2000). Memory-a
Century of Consolidation. Science.

[77] Yuste R., Bonhoeffer T. (2001). Morphological Changes in Dendritic Spines Associated
with Long-Term Synaptic Plasticity. Annu. Rev.
Neurosci..

[78] Nikonenko I., Jourdain P., Alberi S., Toni N., Muller D. (2002). Activity-induced
Changes of Spine Morphology. Hippocampus.

[79] Engert F., Bonhoeffer T. (1999). Dendritic
Spine Changes Associated with Hippocampal
Long-Term Synaptic Plasticity. Nature.

[80] Matsuzaki M., Honkura N., Ellis-Davies G. C. R., Kasai H. (2004). Structural Basis of
Long-Term Potentiation in Single Dendritic Spines. Nature.

[81] Tanaka J., Horiike Y., Matsuzaki M., Miyazaki T., Ellis-Davies G. C. R., Kasai H. (2008). Protein Synthesis and
Neurotrophin-Dependent Structural
Plasticity of Single Dendritic Spines. Science.

[82] Bramham C. R. (2008). Local Protein
Synthesis, Actin Dynamics, and LTP Consolidation. Curr. Opin. Neurobiol..

[83] Matus A. (2000). Actin-Based
Plasticity in Dendritic Spines. Science.

[84] Maletic-Savatic M., Malinow R., Svoboda K. (1999). Rapid Dendritic Morphogenesis in
CA1 Hippocampal Dendrites Induced by Synaptic Activity. Science.

[85] Wittenmayer N., Körber C., Liu H., Kremer T., Varoqueaux F., Chapman E. R., Brose N., Kuner T., Dresbach T. (2009). Postsynaptic
Neuroligin1 Regulates Presynaptic Maturation. Proc. Natl. Acad. Sci. U. S. A..

[86] Togashi H., Abe K., Mizoguchi A., Takaoka K., Chisaka O., Takeichi M. (2002). Cadherin Regulates
Dendritic Spine Morphogenesis. Neuron.

[87] Murase S., Mosser E., Schuman E. M. (2002). Depolarization Drives β-Catenin
into Neuronal Spines Promoting Changes in Synaptic Structure and Function. Neuron.

[88] Lamprecht R., LeDoux J. (2004). Structural Plasticity and Memory. Nat. Rev. Neurosci..

[89] Lai C. S. W., Franke T. F., Gan W.-B. (2012). Opposite
Effects of Fear Conditioning
and Extinction on Dendritic Spine Remodelling. Nature.

[90] Nagel G., Szellas T., Huhn W., Kateriya S., Adeishvili N., Berthold P., Ollig D., Hegemann P., Bamberg E. (2003). Channelrhodopsin-2,
a Directly Light-Gated Cation-Selective Membrane Channel. Proc. Natl. Acad. Sci. U. S. A..

[91] Applebury M. L., Hargrave P. A. (1986). Molecular Biology
of the Visual Pigments. Vision Res..

[92] Arshavsky V. Y., Wensel T. G. (2013). Timing Is Everything: GTPase Regulation in Phototransduction. Investig. Opthalmology Vis. Sci..

[93] Nelson, R. ; Connaughton, V. Bipolar Cell Pathways in the Vertebrate Retina. In Webvision: The Organization of the Retina and Visual System [Internet]; Kolb, H. ; Fernandez, E. ; Jones, B. , Eds.; University of Utah Health Sciences Center: Salt Lake City (UT), 2007 May 24; updated 2012 Jan 20. https://www.ncbi.nlm.nih.gov/books/NBK11521/ (accessed 2025–07–31).

[94] Tinsley J. N., Molodtsov M. I., Prevedel R., Wartmann D., Espigulé-Pons J., Lauwers M., Vaziri A. (2016). Direct Detection of a Single Photon
by Humans. Nat. Commun..

[95] Szobota S., Gorostiza P., Del Bene F., Wyart C., Fortin D. L., Kolstad K. D., Tulyathan O., Volgraf M., Numano R., Aaron H. L., Scott E. K., Kramer R. H., Flannery J., Baier H., Trauner D., Isacoff E. Y. (2007). Remote Control of
Neuronal Activity with a Light-Gated Glutamate Receptor. Neuron.

[96] Xiong H., Xu Y., Kim B., Rha H., Zhang B., Li M., Yang G.-F., Kim J. S. (2023). Photo-Controllable
Biochemistry:
Exploiting the Photocages in Phototherapeutic Window. Chem..

[97] Banghart M., Borges K., Isacoff E., Trauner D., Kramer R. H. (2004). Light-Activated
Ion Channels for Remote Control of Neuronal Firing. Nat. Neurosci..

[98] Pan Z.-H., Bi A., Ma Y.-P., Olshevskaya E., Dizhoor A. M. (2005). Functional Expression
of a Directly Light-Gated Membrane Channel in Mammalian Retinal Neurons:
A Potential Strategy for Restoring Light Sensitivity to the Retina
After Photoreceptor Degeneration. Invest. Ophthalmol.
Vis. Sci..

[99] Wietek, J. ; Prigge, M. Enhancing Channelrhodopsins: An Overview. In Optogenetics; Kianianmomeni, A. , Ed.; Methods in Molecular Biology; Springer New York: New York, NY, 2016; Vol. 1408, pp 141–165. 10.1007/978-1-4939-3512-3_10. 26965121

[100] Mohammad, F. ; Stewart, J. C. ; Ott, S. ; Chlebikova, K. ; Chua, J. Y. ; Koh, T.-W. ; Ho, J. ; Claridge-Chang, A. Optogenetic Inhibition of Behavior with Anion Channelrhodopsins. October 20, Nat. Methods 2017, 14, 271-274 10.1038/nmeth.4148. 28114289

[101] Li H., Sineshchekov O. A., Wu G., Spudich J. L. (2016). In Vitro
Activity of a Purified Natural Anion Channelrhodopsin. J. Biol. Chem..

[102] Zhang F., Wang L.-P., Brauner M., Liewald J. F., Kay K., Watzke N., Wood P. G., Bamberg E., Nagel G., Gottschalk A., Deisseroth K. (2007). Multimodal Fast Optical Interrogation
of Neural Circuitry. Nature.

[103] Keck T., Scheuss V., Jacobsen R. I., Wierenga C. J., Eysel U. T., Bonhoeffer T., Hübener M. (2011). Loss of Sensory
Input Causes Rapid Structural Changes of Inhibitory Neurons in Adult
Mouse Visual Cortex. Neuron.

[104] Wietek J., Beltramo R., Scanziani M., Hegemann P., Oertner T. G., Wiegert J. S. (2015). An Improved Chloride-Conducting
Channelrhodopsin for Light-Induced Inhibition of Neuronal Activity
in Vivo. Sci. Rep..

[105] Gunaydin L. A., Yizhar O., Berndt A., Sohal V. S., Deisseroth K., Hegemann P. (2010). Ultrafast Optogenetic
Control. Nat. Neurosci..

[106] Berndt A., Yizhar O., Gunaydin L. A., Hegemann P., Deisseroth K. (2009). Bi-Stable Neural State Switches. Nat. Neurosci..

[107] Klapoetke N. C., Murata Y., Kim S. S., Pulver S. R., Birdsey-Benson A., Cho Y. K., Morimoto T. K., Chuong A. S., Carpenter E. J., Tian Z., Wang J., Xie Y., Yan Z., Zhang Y., Chow B. Y., Surek B., Melkonian M., Jayaraman V., Constantine-Paton M., Wong G. K.-S., Boyden E. S. (2014). Independent
Optical Excitation of Distinct Neural Populations. Nat. Methods.

[108] Lin J. Y., Knutsen P. M., Muller A., Kleinfeld D., Tsien R. Y. (2013). ReaChR: A Red-Shifted Variant of Channelrhodopsin Enables
Deep Transcranial Optogenetic Excitation. Nat.
Neurosci..

[109] Marshel J. H., Kim Y. S., Machado T. A., Quirin S., Benson B., Kadmon J., Raja C., Chibukhchyan A., Ramakrishnan C., Inoue M., Shane J. C., McKnight D. J., Yoshizawa S., Kato H. E., Ganguli S., Deisseroth K. (2019). Cortical Layer-Specific
Critical Dynamics Triggering Perception. Science.

[110] Jiayi, Z. ; Laiwalla, F. ; Kim, J. A. ; Urabe, H. ; Van Wagenen, R. ; 1, B. W. ; Nurmikko, A. V. A Microelectrode Array Incorporating an Optical Waveguide Device for Stimulation and Spatiotemporal Electrical Recording of Neural Activity. In 2009 Annual International Conference of the IEEE Engineering in Medicine and Biology Society; IEEE: Minneapolis, MN, 2009; pp 2046–2049. 10.1109/IEMBS.2009.5333947. 19964571

[111] Son Y., Jenny Lee H., Kim J., Shin H., Choi N., Justin Lee C., Yoon E.-S., Yoon E., Wise K. D., Geun
Kim T., Cho I.-J. (2015). In Vivo Optical Modulation of Neural
Signals Using Monolithically Integrated Two-Dimensional Neural Probe
Arrays. Sci. Rep..

[112] McAlinden N., Cheng Y., Scharf R., Xie E., Gu E., Reiche C. F., Sharma R., Tathireddy P., Tathireddy P., Rieth L., Blair S., Mathieson K. (2019). Multisite
microLED Optrode Array for Neural Interfacing. Neurophotonics.

[113] Lin X., Wang Y., Chen X., Yang R., Wang Z., Feng J., Wang H., Lai K. W. C., He J., Wang F., Shi P. (2017). Multiplexed Optogenetic
Stimulation
of Neurons with Spectrum-Selective Upconversion Nanoparticles. Adv. Healthc. Mater..

[114] Ozden I., Wang J., Lu Y., May T., Lee J., Goo W., O’Shea D. J., Kalanithi P., Diester I., Diagne M., Deisseroth K., Shenoy K. V., Nurmikko A. V. (2013). A Coaxial Optrode as Multifunction
Write-Read Probe for Optogenetic Studies in Non-Human Primates. J. Neurosci. Methods.

[115] Yakushenko A., Gong Z., Maybeck V., Hofmann B., Gu E., Dawson M. D., Offenhaeusser A., Wolfrum B. (2013). On-Chip Optical Stimulation
and Electrical Recording from Cells. J. Biomed.
Opt..

[116] Hochbaum D. R., Zhao Y., Farhi S. L., Klapoetke N., Werley C. A., Kapoor V., Zou P., Kralj J. M., Maclaurin D., Smedemark-Margulies N., Saulnier J. L., Boulting G. L., Straub C., Cho Y. K., Melkonian M., Wong G. K.-S., Harrison D. J., Murthy V. N., Sabatini B. L., Boyden E. S., Campbell R. E., Cohen A. E. (2014). All-Optical Electrophysiology
in Mammalian Neurons Using Engineered Microbial Rhodopsins. Nat. Methods.

[117] Nagase M., Nagashima T., Hamada S., Morishima M., Tohyama S., Arima-Yoshida F., Hiyoshi K., Hirano T., Ohtsuka T., Watabe A. M. (2024). All-Optical Presynaptic Plasticity
Induction by Photoactivated Adenylyl Cyclase Targeted to Axon Terminals. Cell Rep. Methods.

[118] Bowen Z., De Zoysa D., Shilling-Scrivo K., Aghayee S., Di Salvo G., Smirnov A., Kanold P. O., Losert W. (2024). NeuroART: Real-Time Analysis and Targeting of Neuronal
Population Activity during Calcium Imaging for Informed Closed-Loop
Experiments. eneuro.

[119] Mühlhäuser W. W. D., Fischer A., Weber W., Radziwill G. (2017). Optogenetics - Bringing Light into
the Darkness of
Mammalian Signal Transduction. Biochim. Biophys.
Acta BBA - Mol. Cell Res..

[120] Quail P. H. (2002). Phytochrome Photosensory Signalling
Networks. Nat. Rev. Mol. Cell Biol..

[121] Simó R., Villarroel M., Corraliza L., Hernández C., Garcia-Ramírez M. (2010). The Retinal
Pigment
Epithelium: Something More than a Constituent of the Blood-Retinal
Barrier—Implications for the Pathogenesis of Diabetic Retinopathy. J. Biomed. Biotechnol..

[122] Palczewski K. (2012). Chemistry and Biology of Vision. J. Biol. Chem..

[123] Guo Y., Beyle F. E., Bold B. M., Watanabe H. C., Koslowski A., Thiel W., Hegemann P., Marazzi M., Elstner M. (2016). Active Site
Structure and Absorption Spectrum of Channelrhodopsin-2 Wild-Type
and C128T Mutant. Chem. Sci..

[124] Woods G. A., Rommelfanger N. J., Hong G. (2020). Bioinspired Materials
for In Vivo Bioelectronic Neural Interfaces. Matter.

[125] Luan L., Robinson J. T., Aazhang B., Chi T., Yang K., Li X., Rathore H., Singer A., Yellapantula S., Fan Y., Yu Z., Xie C. (2020). Recent Advances
in Electrical Neural Interface Engineering: Minimal Invasiveness,
Longevity and Scalability. Neuron.

[126] Passaro A. P., Stice S. L. (2021). Electrophysiological Analysis of
Brain Organoids: Current Approaches and Advancements. Front. Neurosci..

[127] Xiang Z., Yen S., Sheshadri S., Wang J., Lee S., Liu Y., Liao L., Thakor N. V., Lee C. (2016). Progress of Flexible
Electronics
in Neural Interfacing - A Self-Adaptive Non-Invasive Neural Ribbon
Electrode for Small Nerves Recording. Adv. Mater..

[128] Zhang M., Tang Z., Liu X., Van Der
Spiegel J. (2020). Electronic Neural Interfaces. Nat. Electron..

[129] Bettinger C. J., Ecker M., Yoshida Kozai T. D., Malliaras G. G., Meng E., Voit W. (2020). Recent Advances in
Neural Interfaces—Materials Chemistry to Clinical Translation. MRS Bull..

[130] Obidin N., Tasnim F., Dagdeviren C. (2020). The Future
of Neuroimplantable Devices: A Materials Science and Regulatory Perspective. Adv. Mater..

[131] Wu N., Wan S., Su S., Huang H., Dou G., Sun L. (2021). Electrode Materials for Brain-Machine Interface: A Review. InfoMat.

[132] Minev I. R., Musienko P., Hirsch A., Barraud Q., Wenger N., Moraud E. M., Gandar J., Capogrosso M., Milekovic T., Asboth L., Torres R. F., Vachicouras N., Liu Q., Pavlova N., Duis S., Larmagnac A., Vörös J., Micera S., Suo Z., Courtine G., Lacour S. P. (2015). Electronic Dura Mater for Long-Term
Multimodal Neural
Interfaces. Science.

[133] Barrese J. C., Rao N., Paroo K., Triebwasser C., Vargas-Irwin C., Franquemont L., Donoghue J. P. (2013). Failure Mode Analysis
of Silicon-Based Intracortical Microelectrode Arrays in Non-Human
Primates. J. Neural Eng..

[134] Szarowski D. H., Andersen M. D., Retterer S., Spence A. J., Isaacson M., Craighead H. G., Turner J. N., Shain W. (2003). Brain Responses
to Micro-Machined Silicon Devices. Brain Res..

[135] Otte E., Vlachos A., Asplund M. (2022). Engineering Strategies
towards Overcoming Bleeding and Glial Scar Formation around Neural
Probes. Cell Tissue Res..

[136] Fu T.-M., Hong G., Zhou T., Schuhmann T. G., Viveros R. D., Lieber C. M. (2016). Stable Long-Term
Chronic Brain Mapping
at the Single-Neuron Level. Nat. Methods.

[137] Yang X., Forró C., Li T. L., Miura Y., Zaluska T. J., Tsai C.-T., Kanton S., McQueen J. P., Chen X., Mollo V., Santoro F., Paşca S. P., Cui B. (2024). Kirigami Electronics for Long-Term Electrophysiological Recording
of Human Neural Organoids and Assembloids. Nat.
Biotechnol..

[138] Yang X., Zhou T., Zwang T. J., Hong G., Zhao Y., Viveros R. D., Fu T.-M., Gao T., Lieber C. M. (2019). Bioinspired Neuron-like Electronics. Nat. Mater..

[139] Ferlauto L., Vagni P., Fanelli A., Zollinger E. G., Monsorno K., Paolicelli R. C., Ghezzi D. (2021). All-Polymeric Transient
Neural Probe for Prolonged in-Vivo Electrophysiological Recordings. Biomaterials.

[140] Bae J.-Y., Hwang G.-S., Kim Y.-S., Jeon J., Chae M., Kim J.-W., Lee S., Kim S., Lee S.-H., Choi S.-G., Lee J.-Y., Lee J.-H., Kim K.-S., Park J.-H., Lee W.-J., Kim Y.-C., Lee K.-S., Kim J., Lee H., Hyun J. K., Kim J.-Y., Kang S.-K. (2024). A Biodegradable
and Self-Deployable
Electronic Tent Electrode for Brain Cortex Interfacing. Nat. Electron..

[141] Tringides C. M., Vachicouras N., De Lázaro I., Wang H., Trouillet A., Seo B. R., Elosegui-Artola A., Fallegger F., Shin Y., Casiraghi C., Kostarelos K., Lacour S. P., Mooney D. J. (2021). Viscoelastic Surface
Electrode Arrays to Interface with Viscoelastic Tissues. Nat. Nanotechnol..

[142] Yang H., Zhu Z., Ni S., Wang X., Nie Y., Tao C., Zou D., Jiang W., Zhao Y., Zhou Z., Sun L., Li M., Tao T. H., Liu K., Wei X. (2025). Silk Fibroin-Based
Bioelectronic Devices for High-Sensitivity,
Stable, and Prolonged in Vivo Recording. Biosens.
Bioelectron..

[143] Kang S.-K., Murphy R. K. J., Hwang S.-W., Lee S. M., Harburg D. V., Krueger N. A., Shin J., Gamble P., Cheng H., Yu S., Liu Z., McCall J. G., Stephen M., Ying H., Kim J., Park G., Webb R. C., Lee C. H., Chung S., Wie D. S., Gujar A. D., Vemulapalli B., Kim A. H., Lee K.-M., Cheng J., Huang Y., Lee S. H., Braun P. V., Ray W. Z., Rogers J. A. (2016). Bioresorbable
Silicon Electronic
Sensors for the Brain. Nature.

[144] Cogan S. F. (2008). Neural
Stimulation and Recording Electrodes. Annu.
Rev. Biomed. Eng..

[145] Chen N., Tian L., Patil A. C., Peng S., Yang I. H., Thakor N. V., Ramakrishna S. (2017). Neural Interfaces
Engineered via Micro- and Nanostructured Coatings. Nano Today.

[146] Park Y., Lee G., Jang J., Yun S. M., Kim E., Park J. (2021). Liquid Metal-Based Soft Electronics for Wearable Healthcare. Adv. Healthc. Mater..

[147] Driscoll N., Richardson A. G., Maleski K., Anasori B., Adewole O., Lelyukh P., Escobedo L., Cullen D. K., Lucas T. H., Gogotsi Y., Vitale F. (2018). Two-Dimensional Ti_3_ C_2_ MXene for High-Resolution Neural Interfaces. ACS Nano.

[148] Devi M., Vomero M., Fuhrer E., Castagnola E., Gueli C., Nimbalkar S., Hirabayashi M., Kassegne S., Stieglitz T., Sharma S. (2021). Carbon-Based Neural
Electrodes: Promises and Challenges. J. Neural
Eng..

[149] Xuan H., Wu S., Jin Y., Wei S., Xiong F., Xue Y., Li B., Yang Y., Yuan H. (2023). A Bioinspired Self-Healing Conductive
Hydrogel Promoting Peripheral
Nerve Regeneration. Adv. Sci..

[150] Rashid R. B., Ji X., Rivnay J. (2021). Organic Electrochemical
Transistors in Bioelectronic Circuits. Biosens.
Bioelectron..

[151] Rivnay J., Inal S., Salleo A., Owens R. M., Berggren M., Malliaras G. G. (2018). Organic
Electrochemical Transistors. Nat. Rev. Mater..

[152] Srivastava S. B., Melikov R., Yildiz E., Dikbas U. M., Sadeghi S., Kavakli I. H., Sahin A., Nizamoglu S. (2021). Bulk-Heterojunction
Photocapacitors with High Open-Circuit Voltage for Low Light Intensity
Photostimulation of Neurons. J. Mater. Chem.
C.

[153] Savva A., Hama A., Herrera-López G., Schmidt T., Migliaccio L., Steiner N., Kawan M., Fiumelli H., Magistretti P. J., McCulloch I., Baran D., Gasparini N., Schindl R., Głowacki E. D., Inal S. (2023). Photo-Chemical Stimulation
of Neurons with Organic Semiconductors. Adv.
Sci..

[154] Rand D., Jakešová M., Lubin G., Vėbraitė I., David-Pur M., Đerek V., Cramer T., Sariciftci N. S., Hanein Y., Głowacki E. D. (2018). Direct Electrical Neurostimulation
with Organic Pigment Photocapacitors. Adv. Mater..

[155] Liu S., Wang Y., Zhao Y., Liu L., Sun S., Zhang S., Liu H., Liu S., Li Y., Yang F., Jiao M., Sun X., Zhang Y., Liu R., Mu X., Wang H., Zhang S., Yang J., Xie X., Duan X., Zhang J., Hong G., Zhang X., Ming D. (2024). A Nanozyme-Based Electrode for High-Performance Neural Recording. Adv. Mater..

[156] Duan W., Robles U. A., Poole-Warren L., Esrafilzadeh D. (2024). Bioelectronic Neural Interfaces: Improving Neuromodulation
Through Organic Conductive Coatings. Adv. Sci..

[157] Carnicer-Lombarte, A. ; Chen, S.-T. ; Malliaras, G. G. ; Barone, D. G. Foreign Body Reaction to Implanted Biomaterials and Its Impact in Nerve Neuroprosthetics. Front. Bioeng. Biotechnol. 2021, 9, 10.3389/fbioe.2021.622524. PMC8081831 33937212

[158] Mariano A., Lubrano C., Bruno U., Ausilio C., Dinger N. B., Santoro F. (2022). Advances in Cell-Conductive
Polymer
Biointerfaces and Role of the Plasma Membrane. Chem. Rev..

[159] Goding J. A., Gilmour A. D., Aregueta-Robles U. A., Hasan E. A., Green R. A. (2018). Living Bioelectronics: Strategies
for Developing an Effective Long-Term Implant with Functional Neural
Connections. Adv. Funct. Mater..

[160] Pérez-Prieto N., Delgado-Restituto M. (2021). Recording
Strategies for High Channel
Count, Densely Spaced Microelectrode Arrays. Front. Neurosci..

[161] Obien, M. E. J. ; Deligkaris, K. ; Bullmann, T. ; Bakkum, D. J. ; Frey, U. Revealing Neuronal Function through Microelectrode Array Recordings. Front. Neurosci. 2015, 8, 10.3389/fnins.2014.00423. PMC4285113 25610364

[162] Liu C., Chen H., Wang S., Liu Q., Jiang Y.-G., Zhang D. W., Liu M., Zhou P. (2020). Two-Dimensional Materials
for next-Generation Computing Technologies. Nat. Nanotechnol..

[163] Zhou A., Santacruz S. R., Johnson B. C., Alexandrov G., Moin A., Burghardt F. L., Rabaey J. M., Carmena J. M., Muller R. (2019). A Wireless and Artefact-Free 128-Channel Neuromodulation
Device for Closed-Loop Stimulation and Recording in Non-Human Primates. Nat. Biomed. Eng..

[164] Kim C. Y., Ku M. J., Qazi R., Nam H. J., Park J. W., Nam K. S., Oh S., Kang I., Jang J.-H., Kim W. Y., Kim J.-H., Jeong J.-W. (2021). Soft Subdermal
Implant Capable of Wireless Battery Charging and Programmable Controls
for Applications in Optogenetics. Nat. Commun..

[165] Szuts T. A., Fadeyev V., Kachiguine S., Sher A., Grivich M. V., Agrochão M., Hottowy P., Dabrowski W., Lubenov E. V., Siapas A. G., Uchida N., Litke A. M., Meister M. (2011). A Wireless Multi-Channel
Neural Amplifier for Freely Moving Animals. Nat. Neurosci..

[166] Zhao Z., Spyropoulos G. D., Cea C., Gelinas J. N., Khodagholy D. (2022). Ionic Communication
for Implantable Bioelectronics. Sci. Adv..

[167] Cea C., Zhao Z., Wisniewski D. J., Spyropoulos G. D., Polyravas A., Gelinas J. N., Khodagholy D. (2023). Integrated
Internal Ion-Gated Organic Electrochemical Transistors for Stand-Alone
Conformable Bioelectronics. Nat. Mater..

[168] Seo D., Neely R. M., Shen K., Singhal U., Alon E., Rabaey J. M., Carmena J. M., Maharbiz M. M. (2016). Wireless Recording
in the Peripheral Nervous System with Ultrasonic Neural Dust. Neuron.

[169] Piech D. K., Johnson B. C., Shen K., Ghanbari M. M., Li K. Y., Neely R. M., Kay J. E., Carmena J. M., Maharbiz M. M., Muller R. (2020). A Wireless Millimetre-Scale Implantable
Neural Stimulator with Ultrasonically Powered Bidirectional Communication. Nat. Biomed. Eng..

[170] Won S. M., Cai L., Gutruf P., Rogers J. A. (2023). Wireless
and Battery-Free Technologies for Neuroengineering. Nat. Biomed. Eng..

[171] Abu Shihada J., Jung M., Decke S., Koschinski L., Musall S., Rincón Montes V., Offenhäusser A. (2024). Highly Customizable
3D Microelectrode Arrays for In Vitro and In Vivo Neuronal Tissue
Recordings. Adv. Sci..

[172] Shokoohimehr P., Cepkenovic B., Milos F., Bednár J., Hassani H., Maybeck V., Offenhäusser A. (2022). High-Aspect-Ratio
Nanoelectrodes Enable Long-Term Recordings of Neuronal Signals with
Subthreshold Resolution. Small.

[173] Yuk H., Lu B., Zhao X. (2019). Hydrogel Bioelectronics. Chem. Soc. Rev..

[174] Fromherz P., Offenhäusser A., Vetter T., Weis J. (1991). A Neuron-Silicon
Junction: A Retzius Cell of the Leech on an Insulated-Gate Field-Effect
Transistor. Science.

[175] Weis R., Fromherz P. (1997). Frequency Dependent
Signal Transfer
in Neuron Transistors. Phys. Rev. E.

[176] Spira M. E., Hai A. (2013). Multi-Electrode Array
Technologies
for Neuroscience and Cardiology. Nat. Nanotechnol..

[177] Habibey R., Rojo Arias J. E., Striebel J., Busskamp V. (2022). Microfluidics
for Neuronal Cell and Circuit Engineering. Chem.
Rev..

[178] Lewandowska M. K., Bakkum D. J., Rompani S. B., Hierlemann A. (2015). Recording
Large Extracellular Spikes in Microchannels along Many Axonal Sites
from Individual Neurons. PLoS One.

[179] Forró C., Thompson-Steckel G., Weaver S., Weydert S., Ihle S., Dermutz H., Aebersold M. J., Pilz R., Demkó L., Vörös J. (2018). Modular Microstructure
Design to Build Neuronal Networks of Defined Functional Connectivity. Biosens. Bioelectron..

[180] Buentello D. C., García-Corral M., Trujillo-de
Santiago G., Alvarez M. M. (2024). Neuron(s)-on-a-Chip: A Review of
the Design and Use of Microfluidic Systems for Neural Tissue Culture. IEEE Rev. Biomed. Eng..

[181] Bruno U., Mariano A., Santoro F. (2021). A Systems Theory Approach
to Describe Dynamic Coupling at the Cell-Electrode Interface. APL Mater..

[182] Dipalo M., Amin H., Lovato L., Moia F., Caprettini V., Messina G. C., Tantussi F., Berdondini L., De Angelis F. (2017). Intracellular and Extracellular Recording of Spontaneous
Action Potentials in Mammalian Neurons and Cardiac Cells with 3D Plasmonic
Nanoelectrodes. Nano Lett..

[183] Santoro F., Schnitker J., Panaitov G., Offenhäusser A. (2013). On Chip Guidance
and Recording of Cardiomyocytes with 3D Mushroom-Shaped Electrodes. Nano Lett..

[184] Lunghi A., Mariano A., Bianchi M., Dinger N. B., Murgia M., Rondanina E., Toma A., Greco P., Di Lauro M., Santoro F., Fadiga L., Biscarini F. (2022). Flexible Neural
Interfaces Based on 3D PEDOT:PSS Micropillar Arrays. Adv. Mater. Interfaces.

[185] Ruggiero A., Criscuolo V., Grasselli S., Bruno U., Ausilio C., Bovio C. L., Bettucci O., Santoro F. (2022). Two-Photon Polymerization Lithography Enabling the
Fabrication of PEDOT:PSS 3D Structures for Bioelectronic Applications. Chem. Commun..

[186] Santoro F., Zhao W., Joubert L.-M., Duan L., Schnitker J., van de Burgt Y., Lou H.-Y., Liu B., Salleo A., Cui L., Cui Y., Cui B. (2017). Revealing
the Cell-Material Interface with Nanometer Resolution by Focused Ion
Beam/Scanning Electron Microscopy. ACS Nano.

[187] Shokoohimehr P., Cepkenovic B., Milos F., Bednár J., Hassani H., Maybeck V., Offenhäusser A. (2022). High-Aspect-Ratio
Nanoelectrodes Enable Long-Term Recordings of Neuronal Signals with
Subthreshold Resolution. Small.

[188] Buccino A. P., Damart T., Bartram J., Mandge D., Xue X., Zbili M., Gänswein T., Jaquier A., Emmenegger V., Markram H., Hierlemann A., Van Geit W. (2024). A Multimodal Fitting
Approach to Construct Single-Neuron Models With Patch Clamp and High-Density
Microelectrode Arrays. Neural Comput..

[189] Belu A., Schnitker J., Bertazzo S., Neumann E., Mayer D., Offenhäusser A., Santoro F. (2016). Ultra-thin Resin Embedding
Method for Scanning Electron Microscopy of Individual Cells on High
and Low Aspect Ratio 3D Nanostructures. J. Microsc..

[190] Sharon A., Shmoel N., Erez H., Jankowski M. M., Friedmann Y., Spira M. E. (2021). Ultrastructural
Analysis of Neuroimplant-Parenchyma
Interfaces Uncover Remarkable Neuroregeneration Along-With Barriers
That Limit the Implant Electrophysiological Functions. Front. Neurosci..

[191] Robinson D. A. (1968). The Electrical
Properties of Metal Microelectrodes. Proc. IEEE.

[192] Cerina M., Piastra M. C., Frega M. (2023). The Potential of in
Vitro Neuronal Networks Cultured on Micro Electrode Arrays for Biomedical
Research. Prog. Biomed. Eng..

[193] Jung M., Abu Shihada J., Decke S., Koschinski L., Graff P. S., Maruri
Pazmino S., Höllig A., Koch H., Musall S., Offenhäusser A., Rincón Montes V. (2025). Flexible 3D *Kirigami* Probes for In
Vitro and In Vivo Neural Applications. Adv.
Mater..

[194] Choi J. S., Lee H. J., Rajaraman S., Kim D.-H. (2021). Recent Advances in Three-Dimensional Microelectrode
Array Technologies for In Vitro and In Vivo Cardiac and Neuronal Interfaces. Biosens. Bioelectron..

[195] Santoro F., Panaitov G., Offenhäusser A. (2014). Defined Patterns
of Neuronal Networks on 3D Thiol-Functionalized Microstructures. Nano Lett..

[196] Milos F., Belu A., Mayer D., Maybeck V., Offenhäusser A. (2021). Polymer Nanopillars Induce Increased
Paxillin Adhesion
Assembly and Promote Axon Growth in Primary Cortical Neurons. Adv. Biol..

[197] Teixeira H., Dias C., Aguiar P., Ventura J. (2021). Gold-Mushroom
Microelectrode Arrays and the Quest for Intracellular-Like Recordings:
Perspectives and Outlooks. Adv. Mater. Technol..

[198] Hai A., Shappir J., Spira M. E. (2010). In-Cell
Recordings by Extracellular
Microelectrodes. Nat. Methods.

[199] Xie C., Lin Z., Hanson L., Cui Y., Cui B. (2012). Intracellular
Recording of Action Potentials by Nanopillar Electroporation. Nat. Nanotechnol..

[200] Rincón Montes V., Gehlen J., Ingebrandt S., Mokwa W., Walter P., Müller F., Offenhäusser A. (2020). Development and in Vitro Validation of Flexible Intraretinal
Probes. Sci. Rep..

[201] Soscia D. A., Lam D., Tooker A. C., Enright H. A., Triplett M., Karande P., Peters S. K. G., Sales A. P., Wheeler E. K., Fischer N. O. (2020). A Flexible 3-Dimensional
Microelectrode
Array for *in Vitro* Brain Models. Lab. Chip.

[202] Kireev D., Rincón Montes V., Stevanovic J., Srikantharajah K., Offenhäusser A. (2019). N3-MEA Probes: Scooping Neuronal
Networks. Front. Neurosci..

[203] Le Floch P., Li Q., Lin Z., Zhao S., Liu R., Tasnim K., Jiang H., Liu J. (2022). Stretchable Mesh Nanoelectronics
for 3D Single-Cell Chronic Electrophysiology from Developing Brain
Organoids. Adv. Mater..

[204] Hubel D. H. (1957). Tungsten Microelectrode for Recording
from Single Units. Science.

[205] Wise K. D., Angell J. B., Starr A. (1970). An Integrated-Circuit
Approach to Extracellular Microelectrodes. IEEE
Trans. Biomed. Eng..

[206] Nicolelis M. A. L., Dimitrov D., Carmena J. M., Crist R., Lehew G., Kralik J. D., Wise S. P. (2003). Chronic,
Multisite,
Multielectrode Recordings in Macaque Monkeys. Proc. Natl. Acad. Sci. U. S. A..

[207] Castagnola, E. ; Zheng, X. S. ; Cui, X. T. Flexible and Soft Materials and Devices for Neural Interface. In Handbook of Neuroengineering; Thakor, N. V. , Ed.; Springer Nature: Singapore, 2023; pp 79–139. 10.1007/978-981-16-5540-1_5.

[208] Wang Y., Yang X., Zhang X., Wang Y., Pei W. (2023). Implantable
Intracortical Microelectrodes: Reviewing the Present
with a Focus on the Future. Microsyst. Nanoeng..

[209] Zhou K., Jia Z., Zhou Y., Ding G., Ma X.-Q., Niu W., Han S.-T., Zhao J., Zhou Y. (2023). Covalent Organic Frameworks
for Neuromorphic Devices. J. Phys. Chem. Lett..

[210] KılınçBülbül D., Walston S. T., Duvan F. T., Garrido J. A., Güçlü B. (2024). Decoding Sensorimotor
Information from Somatosensory Cortex by Flexible Epicortical μECoG
Arrays in Unrestrained Behaving Rats. J. Neural
Eng..

[211] Ria N., Eladly A., Masvidal-Codina E., Illa X., Guimerà A., Hills K., Garcia-Cortadella R., Duvan F. T., Flaherty S. M., Prokop M., Wykes Rob. C., Kostarelos K., Garrido J. A. (2025). Flexible Graphene-Based Neurotechnology for High-Precision
Deep Brain Mapping and Neuromodulation in Parkinsonian Rats. Nat. Commun..

[212] Koschinski L., Lenyk B., Jung M., Lenzi I., Kampa B., Mayer D., Offenhäusser A., Musall S., Rincón Montes V. (2023). Validation of Transparent
and Flexible Neural Implants for Simultaneous Electrophysiology, Functional
Imaging, and Optogenetics. J. Mater. Chem. B.

[213] Ramezani M., Kim J.-H., Liu X., Ren C., Alothman A., De-Eknamkul C., Wilson M. N., Cubukcu E., Gilja V., Komiyama T., Kuzum D. (2024). High-Density Transparent
Graphene Arrays for Predicting Cellular Calcium Activity at Depth
from Surface Potential Recordings. Nat. Nanotechnol..

[214] Yasar T. B., Gombkoto P., Vyssotski A. L., Vavladeli A. D., Lewis C. M., Wu B., Meienberg L., Lundegardh V., Helmchen F., Von Der Behrens W., Yanik M. F. (2024). Months-Long Tracking of Neuronal Ensembles Spanning
Multiple Brain Areas with Ultra-Flexible Tentacle Electrodes. Nat. Commun..

[215] Wang P., Wu E. G., Uluşan H., Phillips A. J., Hays M. R., Kling A., Zhao E. T., Madugula S., Vilkhu R. S., Vasireddy P. K., Hierlemann A., Hong G., Chichilnisky E. J., Melosh N. A. (2025). Direct-Print Three-Dimensional Electrodes for Large-Scale,
High-Density, and Customizable Neural Interfaces. Adv. Sci..

[216] Brown M. A., Zappitelli K. M., Singh L., Yuan R. C., Bemrose M., Brogden V., Miller D. J., Smear M. C., Cogan S. F., Gardner T. J. (2023). Direct
Laser Writing of 3D Electrodes
on Flexible Substrates. Nat. Commun..

[217] Zardini, A. S. ; Rostami, B. ; Najafi, K. ; Hetrick, V. L. ; Ahmed, O. J. Sea of Electrodes Array (SEA): Extremely Dense and High-Count Silicon-Based Electrode Array Technology for High-Resolution High-Bandwidth Interfacing with 3D Neural Structures. January 26, 2021, 10.1101/2021.01.24.427975.

[218] Jun J. J., Steinmetz N. A., Siegle J. H., Denman D. J., Bauza M., Barbarits B., Lee A. K., Anastassiou C. A., Andrei A., Aydın Ç., Barbic M., Blanche T. J., Bonin V., Couto J., Dutta B., Gratiy S. L., Gutnisky D. A., Häusser M., Karsh B., Ledochowitsch P., Lopez C. M., Mitelut C., Musa S., Okun M., Pachitariu M., Putzeys J., Rich P. D., Rossant C., Sun W., Svoboda K., Carandini M., Harris K. D., Koch C., O’Keefe J., Harris T. D. (2017). Fully Integrated Silicon Probes for
High-Density Recording of Neural Activity. Nature.

[219] Steinmetz N. A., Aydin C., Lebedeva A., Okun M., Pachitariu M., Bauza M., Beau M., Bhagat J., Böhm C., Broux M., Chen S., Colonell J., Gardner R. J., Karsh B., Kloosterman F., Kostadinov D., Mora-Lopez C., O’Callaghan J., Park J., Putzeys J., Sauerbrei B., 1 R. J. J., Vollan A. Z., Wang S., Welkenhuysen M., Ye Z., Dudman J. T., Dutta B., Hantman A. W., Harris K. D., Lee A. K., Moser E. I., O’Keefe J., Renart A., Svoboda K., Häusser M., Haesler S., Carandini M., Harris T. D. (2021). Neuropixels 2.0:
A Miniaturized High-Density Probe for Stable, Long-Term Brain Recordings. Science.

[220] Musk, E. ; Neuralink . An Integrated Brain-Machine Interface Platform with Thousands of Channels. July 17, 2019, 21, e16194 10.2196/16194. PMC6914248 31642810

[221] Ramezani M., Ren Y., Cubukcu E., Kuzum D. (2025). Innovating
beyond Electrophysiology through Multimodal Neural Interfaces. Nat. Rev. Electr. Eng..

[222] Gu Y., Wang C., Kim N., Zhang J., Wang T. M., Stowe J., Nasiri R., Li J., Zhang D., Yang A., Hsu L. H.-H., Dai X., Mu J., Liu Z., Lin M., Li W., Wang C., Gong H., Chen Y., Lei Y., Hu H., Li Y., Zhang L., Huang Z., Zhang X., Ahadian S., Banik P., Zhang L., Jiang X., Burke P. J., Khademhosseini A., McCulloch A. D., Xu S. (2022). Three-Dimensional Transistor
Arrays for Intra- and Inter-Cellular Recording. Nat. Nanotechnol..

[223] Delacour C., Veliev F., Crozes T., Bres G., Minet J., Ionica I., Ernst T., Briançon-Marjollet A., Albrieux M., Villard C. (2021). Neuron-Gated Silicon Nanowire Field
Effect Transistors to Follow Single Spike Propagation within Neuronal
Network. Adv. Eng. Mater..

[224] Zhang A., Lee J.-H., Lieber C. M. (2021). Nanowire-Enabled
Bioelectronics. Nano Today.

[225] Kireev D., Offenhäusser A. (2018). Graphene & Two-Dimensional Devices
for Bioelectronics and Neuroprosthetics. 2D
Mater..

[226] Bonaccini Calia A., Masvidal-Codina E., Smith T. M., Schäfer N., Rathore D., Rodríguez-Lucas E., Illa X., De La Cruz J. M., Del Corro E., Prats-Alfonso E., Viana D., Bousquet J., Hébert C., Martínez-Aguilar J., Sperling J. R., Drummond M., Halder A., Dodd A., Barr K., Savage S., Fornell J., Sort J., Guger C., Villa R., Kostarelos K., Wykes R. C., Guimerà-Brunet A., Garrido J. A. (2022). Full-Bandwidth Electrophysiology of Seizures and Epileptiform
Activity Enabled by Flexible Graphene Microtransistor Depth Neural
Probes. Nat. Nanotechnol..

[227] Kireev D., Brambach M., Seyock S., Maybeck V., Fu W., Wolfrum B., Offenhäusser A. (2017). Graphene Transistors
for Interfacing
with Cells: Towards a Deeper Understanding of Liquid Gating and Sensitivity. Sci. Rep..

[228] Chiang C.-H., Won S. M., Orsborn A. L., Yu K. J., Trumpis M., Bent B., Wang C., Xue Y., Min S., Woods V., Yu C., Kim B. H., Kim S. B., Huq R., Li J., Seo K. J., Vitale F., Richardson A., Fang H., Huang Y., Shepard K., Pesaran B., Rogers J. A., Viventi J. (2020). Development of a Neural Interface
for High-Definition, Long-Term Recording in Rodents and Nonhuman Primates. Sci. Transl. Med..

[229] Garcia-Cortadella R., Schwesig G., Jeschke C., Illa X., Gray A. L., Savage S., Stamatidou E., Schiessl I., Masvidal-Codina E., Kostarelos K., Guimerà-Brunet A., Sirota A., Garrido J. A. (2021). Graphene
Active Sensor Arrays for Long-Term and Wireless Mapping of Wide Frequency
Band Epicortical Brain Activity. Nat. Commun..

[230] Penttonen M., Nurminen N., Miettinen R., Sirviö J., Henze D. A., Csicsvári J., Buzsáki G. (1999). Ultra-Slow Oscillation (0.025 Hz) Triggers Hippocampal
Afterdischarges in Wistar Rats. Neuroscience.

[231] Kang H., Kim J.-Y., Choi Y.-K., Nam Y. (2017). Feasibility
Study of Extended-Gate-Type Silicon Nanowire Field-Effect Transistors
for Neural Recording. Sensors.

[232] Shi W., Guo Y., Liu Y. (2020). When Flexible
Organic Field-Effect
Transistors Meet Biomimetics: A Prospective View of the Internet of
Things. Adv. Mater..

[233] Tao J., Sun W., Lu L. (2022). Organic Small
Molecule Semiconductor
Materials for OFET-Based Biosensors. Biosens.
Bioelectron..

[234] Song J., Liu H., Zhao Z., Lin P., Yan F. (2024). Flexible Organic Transistors for Biosensing: Devices
and Applications. Adv. Mater..

[235] Nawaz A., Liu Q., Leong W. L., Fairfull-Smith K. E., Sonar P. (2021). Organic Electrochemical Transistors
for In Vivo Bioelectronics. Adv. Mater..

[236] Spyropoulos G. D., Gelinas J. N., Khodagholy D. (2019). Internal Ion-Gated
Organic Electrochemical Transistor: A Building Block for Integrated
Bioelectronics. Sci. Adv..

[237] Cea C., Spyropoulos G. D., Jastrzebska-Perfect P., Ferrero J. J., Gelinas J. N., Khodagholy D. (2020). Enhancement-Mode
Ion-Based Transistor as a Comprehensive Interface and Real-Time Processing
Unit for in Vivo Electrophysiology. Nat. Mater..

[238] Berggren M., Głowacki E. D., Simon D. T., Stavrinidou E., Tybrandt K. (2022). *In Vivo* Organic Bioelectronics for
Neuromodulation. Chem. Rev..

[239] Tyrrell J. E., Boutelle M. G., Campbell A. J. (2021). Measurement
of Electrophysiological
Signals In Vitro Using High-Performance Organic Electrochemical Transistors. Adv. Funct. Mater..

[240] Spanu A., Martines L., Bonfiglio A. (2021). Interfacing
Cells with Organic Transistors: A Review of in Vitro and in Vivo Applications. Lab. Chip.

[241] Lu Y., Jia Y., Yu C. (2021). Recent Advances in Power Supply Strategies
for Untethered Neural Implants. J. Micromechanics
Microengineering.

[242] Burton A., Obaid S. N., Vázquez-Guardado A., Schmit M. B., Stuart T., Cai L., Chen Z., Kandela I., Haney C. R., Waters E. A., Cai H., Rogers J. A., Lu L., Gutruf P. (2020). Wireless, Battery-Free
Subdermally Implantable Photometry Systems for Chronic Recording of
Neural Dynamics. Proc. Natl. Acad. Sci. U. S.
A..

[243] Photodetectors and Solar Cells. In Physics of Semiconductor Devices; John Wiley & Sons, Ltd, 2006; pp 663–742. 10.1002/9780470068328.ch13.

[244] Goetz G. A., Palanker D. V. (2016). Electronic Approaches
to Restoration
of Sight. Rep. Prog. Phys..

[245] Aria M. M., Srivastava S. B., Sekerdag E., Dikbas U. M., Sadeghi S., Pering S. R., Cameron P. J., Gursoy-Ozdemir Y., Kavakli I. H., Nizamoglu S. (2019). Perovskite-Based
Optoelectronic Biointerfaces
for Non-Bias-Assisted Photostimulation of Cells. Adv. Mater. Interfaces.

[246] Karatum O., Kaleli H. N., Eren G. O., Sahin A., Nizamoglu S. (2022). Electrical Stimulation of Neurons with Quantum Dots
via Near-Infrared Light. ACS Nano.

[247] Vagni P., Airaghi Leccardi M. J.
I., Vila C.-H., Zollinger E. G., Sherafatipour G., Wolfensberger T. J., Ghezzi D. (2022). POLYRETINA Restores Light Responses in Vivo in Blind
Göttingen Minipigs. Nat. Commun..

[248] SilveråEjneby M., Jakešová M., Ferrero J. J., Migliaccio L., Sahalianov I., Zhao Z., Berggren M., Khodagholy D., Đerek V., Gelinas J. N., Głowacki E. D. (2022). Chronic
Electrical Stimulation of Peripheral Nerves via Deep-Red Light Transduced
by an Implanted Organic Photocapacitor. Nat.
Biomed. Eng..

[249] Clark A. M., Ingold A., Reiche C. F., Cundy D., Balsor J. L., Federer F., McAlinden N., Cheng Y., Rolston J. D., Rieth L., Dawson M. D., Mathieson K., Blair S., Angelucci A. (2024). An Optrode
Array for Spatiotemporally-Precise Large-Scale Optogenetic Stimulation
of Deep Cortical Layers in Non-Human Primates. Commun. Biol..

[250] Fallon, J. B. ; Carter, P. M. Principles of Recording from and Electrical Stimulation of Neural Tissue. In Neurobionics: The Biomedical Engineering of Neural Prostheses; Shepherd, R. K. , Ed.; Wiley, 2016; pp 89–120. 10.1002/9781118816028.ch4.

[251] Schiavone G., Kang X., Fallegger F., Gandar J., Courtine G., Lacour S. P. (2020). Guidelines to Study
and Develop Soft Electrode Systems for Neural Stimulation. Neuron.

[252] Neher, E. ; Sakmann, B. The Patch Clamp Technique. Sci. Am. 1992, 266, 44. 10.1038/scientificamerican0392-44 1374932

[253] Abbott J., Ye T., Krenek K., Gertner R. S., Ban S., Kim Y., Qin L., Wu W., Park H., Ham D. (2020). A Nanoelectrode Array for Obtaining Intracellular Recordings from
Thousands of Connected Neurons. Nat. Biomed.
Eng..

[254] Suzuki I., Matsuda N., Han X., Noji S., Shibata M., Nagafuku N., Ishibashi Y. (2023). Large-Area
Field Potential Imaging Having Single Neuron Resolution Using 236
880 Electrodes CMOS-MEA Technology. Adv. Sci..

[255] Yuan X., Hierlemann A., Frey U. (2021). Extracellular Recording
of Entire Neural Networks Using a Dual-Mode Microelectrode Array With
19 584 Electrodes and High SNR. IEEE J. Solid-State
Circuits.

[256] Liu R., Chen R., Elthakeb A. T., Lee S. H., Hinckley S., Khraiche M. L., Scott J., Pre D., Hwang Y., Tanaka A., Ro Y. G., Matsushita A. K., Dai X., Soci C., Biesmans S., James A., Nogan J., Jungjohann K. L., Pete D. V., Webb D. B., Zou Y., Bang A. G., Dayeh S. A. (2017). High Density Individually Addressable
Nanowire Arrays Record Intracellular Activity from Primary Rodent
and Human Stem Cell Derived Neurons. Nano Lett..

[257] Liu R., Lee J., Tchoe Y., Pre D., Bourhis A. M., D’Antonio-Chronowska A., Robin G., Lee S. H., Ro Y. G., Vatsyayan R., Tonsfeldt K. J., Hossain L. A., Phipps M. L., Yoo J., Nogan J., Martinez J. S., Frazer K. A., Bang A. G., Dayeh S. A. (2022). Ultra-Sharp
Nanowire Arrays Natively Permeate, Record, and Stimulate Intracellular
Activity in Neuronal and Cardiac Networks. Adv.
Funct. Mater..

[258] Dipalo M., Caprettini V., Bruno G., Caliendo F., Garma L. D., Melle G., Dukhinova M., Siciliano V., Santoro F., De Angelis F. (2019). Membrane Poration
Mechanisms at the Cell-Nanostructure Interface. Adv. Biosyst..

[259] Dipalo M., McGuire A. F., Lou H.-Y., Caprettini V., Melle G., Bruno G., Lubrano C., Matino L., Li X., De Angelis F., Cui B., Santoro F. (2018). Cells Adhering to 3D
Vertical Nanostructures: Cell Membrane Reshaping without Stable Internalization. Nano Lett..

[260] Chen X., Wang F., Kooijmans R., Klink P. C., Boehler C., Asplund M., Roelfsema P. R. (2023). Chronic
Stability of a Neuroprosthesis Comprising Multiple Adjacent Utah Arrays
in Monkeys. J. Neural Eng..

[261] Lewis C. M., Boehler C., Liljemalm R., Fries P., Stieglitz T., Asplund M. (2024). Recording Quality Is
Systematically Related to Electrode Impedance. Adv. Healthc. Mater..

[262] Fan B., Wolfrum B., Robinson J. T. (2021). Impedance
Scaling for Gold and Platinum
Microelectrodes. J. Neural Eng..

[263] Bhadra, N. Physiological Principles of Electrical Stimulation. In Implantable Neuroprostheses for Restoring Function; Elsevier, 2015; pp 13–43. 10.1016/B978-1-78242-101-6.00002-1.

[264] Merrill D. R., Bikson M., Jefferys J. G. R. (2005). Electrical Stimulation
of Excitable Tissue: Design of Efficacious and Safe Protocols. J. Neurosci. Methods.

[265] Boehler C., Carli S., Fadiga L., Stieglitz T., Asplund M. (2020). Tutorial: Guidelines for Standardized
Performance Tests
for Electrodes Intended for Neural Interfaces and Bioelectronics. Nat. Protoc..

[266] Duru J., Maurer B., Giles Doran C., Jelitto R., Küchler J., Ihle S. J., Ruff T., John R., Genocchi B., Vörös J. (2023). Investigation
of the Input-Output Relationship of Engineered Neural Networks Using
High-Density Microelectrode Arrays. Biosens.
Bioelectron..

[267] Shin H., Jeong S., Lee J.-H., Sun W., Choi N., Cho I.-J. (2021). 3D High-Density Microelectrode Array
with Optical Stimulation and Drug Delivery for Investigating Neural
Circuit Dynamics. Nat. Commun..

[268] Ronchi S., Buccino A. P., Prack G., Kumar S. S., Schröter M., Fiscella M., Hierlemann A. (2021). Electrophysiological
Phenotype Characterization of Human iPSC-Derived Neuronal Cell Lines
by Means of High-Density Microelectrode Arrays. Adv. Biol..

[269] Mossink B., Verboven A. H. A., Van Hugte E. J. H., Klein Gunnewiek T. M., Parodi G., Linda K., Schoenmaker C., Kleefstra T., Kozicz T., Van Bokhoven H., Schubert D., Nadif Kasri N., Frega M. (2021). Human Neuronal Networks
on Micro-Electrode Arrays Are a Highly Robust Tool to Study Disease-Specific
Genotype-Phenotype Correlations in Vitro. Stem
Cell Rep..

[270] Shin H., Jeong S., Lee J.-H., Sun W., Choi N., Cho I.-J. (2021). 3D High-Density Microelectrode Array
with Optical Stimulation and Drug Delivery for Investigating Neural
Circuit Dynamics. Nat. Commun..

[271] Bak A., Schmied K., Jakob M. L., Bedogni F., Squire O. A., Gittel B., Jesinghausen M., Schünemann K. D., Weber Y., Kampa B., Van Loo K. M. J., Koch H. (2024). Temporal Dynamics
of Neocortical Development in Organotypic Mouse Brain Cultures: A
Comprehensive Analysis. J. Neurophysiol..

[272] Halfmann C., Rüland T., Müller F., Jehasse K., Kampa B. M. (2023). Electrophysiological
Properties of
Layer 2/3 Pyramidal Neurons in the Primary Visual Cortex of a Retinitis
Pigmentosa Mouse Model (Rd10). Front. Cell.
Neurosci..

[273] Reinhard K., Münch T. A. (2021). Visual
Properties of Human Retinal
Ganglion Cells. PLoS One.

[274] Zhao Z., Zhu H., Li X., Sun L., He F., Chung J. E., Liu D. F., Frank L., Luan L., Xie C. (2023). Ultraflexible Electrode Arrays for
Months-Long High-Density Electrophysiological
Mapping of Thousands of Neurons in Rodents. Nat. Biomed. Eng..

[275] Ghosh, K. Neuralink’s First Human Patient Able to Control Mouse through Thinking, Musk Says. Reuters, February 20, 2024. https://www.reuters.com/business/healthcare-pharmaceuticals/neuralinks-first-human-patient-able-control-mouse-through-thinking-musk-says-2024-02-20/ (accessed 2025–07–31).

[276] Callegari F., Brofiga M., Poggio F., Massobrio P. (2022). Stimulus-Evoked
Activity Modulation of In Vitro Engineered Cortical and Hippocampal
Networks. Micromachines.

[277] Ahtiainen A., Leydolph L., Tanskanen J. M. A., Hunold A., Haueisen J., Hyttinen J. A. K. (2024). Electric Field
Temporal Interference Stimulation of Neurons *in Vitro*. Lab. Chip.

[278] Meng X., Yu X., Lu Y., Pei Z., Wang G., Qi M., Liu R., Zhou J., Guo X., Zhou Z., Wang F. (2023). Electrical Stimulation Induced Structural
3D Human Engineered Neural Tissue with Well-Developed Neuronal Network
and Functional Connectivity. J. Neural Eng..

[279] Gogliettino A. R., Madugula S. S., Grosberg L. E., Vilkhu R. S., Brown J., Nguyen H., Kling A., Hottowy P., Da̧browski W., Sher A., Litke A. M., Chichilnisky E. J. (2023). High-Fidelity
Reproduction of Visual Signals by Electrical Stimulation in the Central
Primate Retina. J. Neurosci..

[280] Rincón Montes V., Gehlen J., Lück S., Mokwa W., Müller F., Walter P., Offenhäusser A. (2019). Toward a Bidirectional
Communication Between Retinal Cells and a Prosthetic Device - A Proof
of Concept. Front. Neurosci..

[281] Lorach H., Galvez A., Spagnolo V., Martel F., Karakas S., Intering N., Vat M., Faivre O., Harte C., Komi S., Ravier J., Collin T., Coquoz L., Sakr I., Baaklini E., Hernandez-Charpak S. D., Dumont G., Buschman R., Buse N., Denison T., van Nes I., Asboth L., Watrin A., Struber L., Sauter-Starace F., Langar L., Auboiroux V., Carda S., Chabardes S., Aksenova T., Demesmaeker R., Charvet G., Bloch J., Courtine G. (2023). Walking Naturally after
Spinal Cord Injury Using a Brain-Spine Interface. Nature.

[282] Neumann W.-J., Steiner L. A., Milosevic L. (2023). Neurophysiological
Mechanisms of Deep Brain Stimulation across Spatiotemporal Resolutions. Brain.

[283] Oehrn C. R., Cernera S., Hammer L. H., Shcherbakova M., Yao J., Hahn A., Wang S., Ostrem J. L., Little S., Starr P. A. (2024). Chronic Adaptive
Deep Brain Stimulation versus Conventional
Stimulation in Parkinson’s Disease: A Blinded Randomized Feasibility
Trial. Nat. Med..

[284] Ertas Y. N., Ozpolat D., Karasu S. N., Ashammakhi N. (2022). Recent Advances
in Cochlear Implant Electrode Array Design Parameters. Micromachines.

[285] Ayton L. N., Barnes N., Dagnelie G., Fujikado T., Goetz G., Hornig R., Jones B. W., Muqit M. M. K., Rathbun D. L., Stingl K., Weiland J. D., Petoe M. A. (2020). An Update
on Retinal Prostheses. Clin. Neurophysiol..

[286] Ganesana M., Lee S. T., Wang Y., Venton B. J. (2017). Analytical
Techniques in Neuroscience: Recent Advances in Imaging, Separation,
and Electrochemical Methods. Anal. Chem..

[287] Xu X., Zuo Y., Chen S., Hatami A., Gu H. (2024). Advancements
in Brain Research: The In Vivo/In Vitro Electrochemical Detection
of Neurochemicals. Biosensors.

[288] Castagnola E., Robbins E. M., Krahe D. D., Wu B., Pwint M. Y., Cao Q., Cui X. T. (2023). Stable In-Vivo Electrochemical
Sensing of Tonic Serotonin Levels Using PEDOT/CNT-Coated Glassy Carbon
Flexible Microelectrode Arrays. Biosens. Bioelectron..

[289] Liu R., Feng Z.-Y., Li D., Jin B., Lan Y., Meng L.-Y. (2022). Recent Trends in Carbon-Based Microelectrodes
as Electrochemical
Sensors for Neurotransmitter Detection: A Review. TrAC Trends Anal. Chem..

[290] White K. A., Kim B. N. (2021). Quantifying Neurotransmitter Secretion
at Single-Vesicle Resolution Using High-Density Complementary Metal-Oxide-Semiconductor
Electrode Array. Nat. Commun..

[291] Liu X., Tong Y., Fang P.-P. (2019). Recent
Development in Amperometric
Measurements of Vesicular Exocytosis. TrAC Trends
Anal. Chem..

[292] Wightman R. M., Jankowski J. A., Kennedy R. T., Kawagoe K. T., Schroeder T. J., Leszczyszyn D. J., Near J. A., Diliberto E. J., Viveros O. H. (1991). Temporally Resolved Catecholamine Spikes Correspond
to Single Vesicle Release from Individual Chromaffin Cells. Proc. Natl. Acad. Sci. U. S. A..

[293] Feng T., Ji W., Tang Q., Wei H., Zhang S., Mao J., Zhang Y., Mao L., Zhang M. (2019). Low-Fouling Nanoporous Conductive Polymer-Coated Microelectrode for
In Vivo Monitoring of Dopamine in the Rat Brain. Anal. Chem..

[294] Rodeberg N. T., Sandberg S. G., Johnson J. A., Phillips P. E. M., Wightman R. M. (2017). Hitchhiker’s Guide to Voltammetry: Acute and
Chronic Electrodes for in Vivo Fast-Scan Cyclic Voltammetry. ACS Chem. Neurosci..

[295] Baur J. E., Kristensen E. W., May L. J., Wiedemann D. J., Wightman R. (1988). Mark. Fast-Scan Voltammetry
of Biogenic Amines. Anal. Chem..

[296] Ammam M., Fransaer J. (2010). Highly Sensitive and Selective Glutamate
Microbiosensor Based on Cast Polyurethane/AC-Electrophoresis Deposited
Multiwalled Carbon Nanotubes and Then Glutamate Oxidase/Electrosynthesized
Polypyrrole/Pt Electrode. Biosens. Bioelectron..

[297] Meng L., Wu P., Chen G., Cai C., Sun Y., Yuan Z. (2009). Low Potential
Detection of Glutamate Based on the Electrocatalytic
Oxidation of NADH at Thionine/Single-Walled Carbon Nanotubes Composite
Modified Electrode. Biosens. Bioelectron..

[298] Akhtar M. H., Hussain K. K., Gurudatt N. G., Shim Y.-B. (2017). Detection
of Ca2+-Induced Acetylcholine Released from Leukemic T-Cells Using
an Amperometric Microfluidic Sensor. Biosens.
Bioelectron..

[299] Kueng A., Kranz C., Mizaikoff B. (2005). Imaging of
ATP Membrane Transport with Dual Micro-Disk Electrodes and Scanning
Electrochemical Microscopy. Biosens. Bioelectron..

[300] Roychoudhury A., Basu S., Jha S. K. (2016). Dopamine Biosensor
Based on Surface Functionalized Nanostructured Nickel Oxide Platform. Biosens. Bioelectron..

[301] Andreescu S., Marty J.-L. (2006). Twenty Years Research in Cholinesterase
Biosensors: From Basic Research to Practical Applications. Biomol. Eng..

[302] Zare I., Choi D., Zhang J., Yaraki M. T., Ghaee A., Nasab S. Z., Taheri-Ledari R., Maleki A., Rahi A., Fan K., Lee J. (2024). Modulating
the Catalytic Activities of Nanozymes for Molecular Sensing. Nano Today.

[303] Jamal M., Hasan M., Mathewson A., Razeeb K. M. (2013). Disposable Sensor
Based on Enzyme-Free Ni Nanowire
Array Electrode to Detect Glutamate. Biosens.
Bioelectron..

[304] Asai K., Ivandini T. A., Einaga Y. (2016). Continuous and Selective
Measurement of Oxytocin and Vasopressin Using Boron-Doped Diamond
Electrodes. Sci. Rep..

[305] Gao X., Wei H., Ma W., Wu W., Ji W., Mao J., Yu P., Mao L. (2024). Inflammation-Free
Electrochemical
in Vivo Sensing of Dopamine with Atomic-Level Engineered Antioxidative
Single-Atom Catalyst. Nat. Commun..

[306] DeRosa M. C., Lin A., Mallikaratchy P., McConnell E. M., McKeague M., Patel R., Shigdar S. (2023). In Vitro Selection
of Aptamers and Their Applications. Nat. Rev.
Methods Primer.

[307] Hu Z., Li Y., Figueroa-Miranda G., Musall S., Li H., Martínez-Roque M. A., Hu Q., Feng L., Mayer D., Offenhäusser A. (2023). Aptamer Based
Biosensor Platforms
for Neurotransmitters Analysis. TrAC Trends
Anal. Chem..

[308] Liu Y., Yu H., Alkhamis O., Moliver J., Xiao Y. (2020). Tuning Biosensor
Cross-Reactivity Using Aptamer Mixtures. Anal.
Chem..

[309] Alkhamis O., Canoura J., Yu H., Liu Y., Xiao Y. (2019). Innovative Engineering and Sensing Strategies for Aptamer-Based Small-Molecule
Detection. TrAC Trends Anal. Chem..

[310] Yu H., Alkhamis O., Canoura J., Liu Y., Xiao Y. (2021). Advances and
Challenges in Small-Molecule DNA Aptamer Isolation, Characterization,
and Sensor Development. Angew. Chem., Int. Ed..

[311] Zhang K., He X., Liu Y., Yu P., Fei J., Mao L. (2017). Highly Selective
Cerebral ATP Assay Based on Micrometer
Scale Ion Current Rectification at Polyimidazolium-Modified Micropipettes. Anal. Chem..

[312] Li X., Jin Y., Zhu F., Liu R., Jiang Y., Jiang Y., Mao L. (2022). Electrochemical Conjugation
of Aptamers
on a Carbon Fiber Microelectrode Enables Highly Stable and Selective
In Vivo Neurosensing. Angew. Chem., Int. Ed..

[313] Seo J.-W., Fu K., Correa S., Eisenstein M., Appel E. A., Soh H. T. (2022). Real-Time Monitoring of Drug Pharmacokinetics
within Tumor Tissue in Live Animals. Sci. Adv..

[314] Álvarez-Martos I., Ferapontova E. E. (2016). Electrochemical
Label-Free Aptasensor for Specific Analysis of Dopamine in Serum in
the Presence of Structurally Related Neurotransmitters. Anal. Chem..

[315] Wu C., Barkova D., Komarova N., Offenhäusser A., Andrianova M., Hu Z., Kuznetsov A., Mayer D. (2022). Highly Selective and Sensitive Detection of Glutamate by an Electrochemical
Aptasensor. Anal. Bioanal. Chem..

[316] Wadhera T., Kakkar D., Wadhwa G., Raj B. (2019). Recent Advances
and Progress in Development of the Field Effect Transistor Biosensor:
A Review. J. Electron. Mater..

[317] Zhao C., Cheung K. M., Huang I.-W., Yang H., Nakatsuka N., Liu W., Cao Y., Man T., Weiss P. S., Monbouquette H. G., Andrews A. M. (2021). Implantable Aptamer-Field-Effect
Transistor Neuroprobes for in Vivo Neurotransmitter Monitoring. Sci. Adv..

[318] Gao Z., Wu G., Song Y., Li H., Zhang Y., Schneider M. J., Qiang Y., Kaszas J., Weng Z., Sun H., Huey B. D., Lai R. Y., Zhang Y. (2022). Multiplexed Monitoring
of Neurochemicals via Electrografting-Enabled Site-Selective Functionalization
of Aptamers on Field-Effect Transistors. Anal.
Chem..

[319] Minamiki T., Sasaki Y., Su S., Minami T. (2019). Development
of Polymer Field-Effect Transistor-Based Immunoassays. Polym. J..

[320] Liang Y., Guo T., Zhou L., Offenhäusser A., Mayer D. (2020). Label-Free Split Aptamer Sensor for Femtomolar Detection of Dopamine
by Means of Flexible Organic Electrochemical Transistors. Materials.

[321] Wang S., Wu M., Liu W., Liu J., Tian Y., Xiao K. (2024). Dopamine Detection
and Integration
in Neuromorphic Devices for Applications in Artificial Intelligence. Device.

[322] Liu C., Zhao Y., Cai X., Xie Y., Wang T., Cheng D., Li L., Li R., Deng Y., Ding H., Lv G., Zhao G., Liu L., Zou G., Feng M., Sun Q., Yin L., Sheng X. (2020). A Wireless,
Implantable Optoelectrochemical Probe for Optogenetic Stimulation
and Dopamine Detection. Microsyst. Nanoeng..

[323] Simon D. T., Kurup S., Larsson K. C., Hori R., Tybrandt K., Goiny M., Jager E. W. H., Berggren M., Canlon B., Richter-Dahlfors A. (2009). Organic Electronics
for Precise Delivery
of Neurotransmitters to Modulate Mammalian Sensory Function. Nat. Mater..

[324] Jonsson A., Song Z., Nilsson D., Meyerson B. A., Simon D. T., Linderoth B., Berggren M. (2015). Therapy Using Implanted
Organic Bioelectronics. Sci. Adv..

[325] Uguz I., Proctor C. M., Curto V. F., Pappa A., Donahue M. J., Ferro M., Owens R. M., Khodagholy D., Inal S., Malliaras G. G. (2017). A Microfluidic
Ion Pump for In Vivo
Drug Delivery. Adv. Mater..

[326] World Health Organization. Epilepsy. World Health Organization (Fact Sheet), February 7, 2024. https://www.who.int/news-room/fact-sheets/detail/epilepsy (accessed 2025–07–31).

[327] Proctor C. M., Slézia A., Kaszas A., Ghestem A., Del Agua I., Pappa A.-M., Bernard C., Williamson A., Malliaras G. G. (2018). Electrophoretic Drug Delivery for Seizure Control. Sci. Adv..

[328] Ash C., Dubec M., Donne K., Bashford T. (2017). Effect of Wavelength
and Beam Width on Penetration in Light-Tissue Interaction Using Computational
Methods. Lasers Med. Sci..

[329] Hart W. L., Kameneva T., Wise A. K., Stoddart P. R. (2019). Biological
Considerations of Optical Interfaces for Neuromodulation. Adv. Opt. Mater..

[330] Xu S., Momin M., Ahmed S., Hossain A., Veeramuthu L., Pandiyan A., Kuo C.-C., Zhou T. (2023). Illuminating the Brain:
Advances and Perspectives in Optoelectronics for Neural Activity Monitoring
and Modulation. Adv. Mater..

[331] Rianna C., Calabuig A., Ventre M., Cavalli S., Pagliarulo V., Grilli S., Ferraro P., Netti P. A. (2015). Reversible
Holographic Patterns on Azopolymers for Guiding Cell Adhesion and
Orientation. ACS Appl. Mater. Interfaces.

[332] De Martino S., Zhang W., Klausen L., Lou H.-Y., Li X., Alfonso F. S., Cavalli S., Netti P. A., Santoro F., Cui B. (2020). Dynamic Manipulation of Cell Membrane Curvature by Light-Driven Reshaping
of Azopolymer. Nano Lett..

[333] Bellani S., Ghadirzadeh A., Meda L., Savoini A., Tacca A., Marra G., Meira R., Morgado J., Di Fonzo F., Antognazza M. R. (2015). Hybrid
Organic/Inorganic Nanostructures
for Highly Sensitive Photoelectrochemical Detection of Dissolved Oxygen
in Aqueous Media. Adv. Funct. Mater..

[334] Gamper N., Zaika O., Li Y., Martin P., Hernandez C. C, Perez M. R, Wang A. Y C, Jaffe D. B, Shapiro M. S (2006). Oxidative
Modification of M-Type K^+^ Channels
as a Mechanism of Cytoprotective Neuronal Silencing. EMBO J..

[335] Bossio, C. ; Abdel Aziz, I. ; Tullii, G. ; Zucchetti, E. ; Debellis, D. ; Zangoli, M. ; Di Maria, F. ; Lanzani, G. ; Antognazza, M. R. Photocatalytic Activity of Polymer Nanoparticles Modulates Intracellular Calcium Dynamics and Reactive Oxygen Species in HEK-293 Cells. Front. Bioeng. Biotechnol. 2018, 6, 10.3389/fbioe.2018.00114. PMC6119808 30211158

[336] Huang Y.-Y., Nagata K., Tedford C. E., McCarthy T., Hamblin M. R. (2013). Low-Level Laser Therapy (LLLT) Reduces
Oxidative Stress
in Primary Cortical Neurons in Vitro. J. Biophotonics.

[337] Martino N., Feyen P., Porro M., Bossio C., Zucchetti E., Ghezzi D., Benfenati F., Lanzani G., Antognazza M. R. (2015). Photothermal
Cellular Stimulation
in Functional Bio-Polymer Interfaces. Sci. Rep..

[338] Lodola F., Rosti V., Tullii G., Desii A., Tapella L., Catarsi P., Lim D., Moccia F., Antognazza M. R. (2019). Conjugated Polymers Optically Regulate
the Fate of
Endothelial Colony-Forming Cells. Sci. Adv..

[339] Đerek, V. ; Rand, D. ; Migliaccio, L. ; Hanein, Y. ; Głowacki, E. D. Untangling Photofaradaic and Photocapacitive Effects in Organic Optoelectronic Stimulation Devices. Front. Bioeng. Biotechnol. 2020, 8, 10.3389/fbioe.2020.00284. PMC7180391 32363183

[340] Xiao G., Song Y., Zhang Y., Xing Y., Zhao H., Xie J., Xu S., Gao F., Wang M., Xing G., Cai X. (2019). Microelectrode Arrays
Modified with Nanocomposites for Monitoring Dopamine and Spike Firings
under Deep Brain Stimulation in Rat Models of Parkinson’s Disease. ACS Sens..

[341] Yang S. M., Shim J. H., Cho H., Jang T., Ko G., Shim J., Kim T. H., Zhu J., Park S., Kim Y. S., Joung S., Choe J. C., Shin J., Lee J. H., Kang Y. M., Cheng H., Jung Y., Lee C., Jang D. P., Hwang S. (2022). Hetero-Integration
of Silicon Nanomembranes
with 2D Materials for Bioresorbable, Wireless Neurochemical System. Adv. Mater..

[342] Medagoda D. I., Ghezzi D. (2021). Organic Semiconductors for Light-Mediated
Neuromodulation. Commun. Mater..

[343] Van Doremaele E. R. W., Ji X., Rivnay J., Van De Burgt Y. (2023). A Retrainable
Neuromorphic Biosensor for On-Chip Learning and Classification. Nat. Electron..

[344] Harikesh P. C., Yang C.-Y., Tu D., Gerasimov J. Y., Dar A. M., Armada-Moreira A., Massetti M., Kroon R., Bliman D., Olsson R., Stavrinidou E., Berggren M., Fabiano S. (2022). Organic Electrochemical
Neurons and
Synapses with Ion Mediated Spiking. Nat. Commun..

[345] Kuzum D., Jeyasingh R. G. D., Lee B., Wong H.-S. P. (2012). Nanoelectronic
Programmable Synapses Based on Phase Change Materials for Brain-Inspired
Computing. Nano Lett..

[346] Yang Y., Gao P., Gaba S., Chang T., Pan X., Lu W. (2012). Observation of Conducting
Filament Growth in Nanoscale
Resistive Memories. Nat. Commun..

[347] Berdan R., Vasilaki E., Khiat A., Indiveri G., Serb A., Prodromakis T. (2016). Emulating
Short-Term Synaptic Dynamics
with Memristive Devices. Sci. Rep..

[348] Pedretti G., Milo V., Ambrogio S., Carboni R., Bianchi S., Calderoni A., Ramaswamy N., Spinelli A. S., Ielmini D. (2017). Memristive Neural Network
for On-Line
Learning and Tracking with Brain-Inspired Spike Timing Dependent Plasticity. Sci. Rep..

[349] Szot K., Speier W., Bihlmayer G., Waser R. (2006). Switching the Electrical Resistance of Individual Dislocations in
Single-Crystalline SrTiO3. Nat. Mater..

[350] Cao G., Meng P., Chen J., Liu H., Bian R., Zhu C., Liu F., Liu Z. (2021). 2D Material
Based Synaptic Devices
for Neuromorphic Computing. Adv. Funct. Mater..

[351] Dong Z., Hua Q., Xi J., Shi Y., Huang T., Dai X., Niu J., Wang B., Wang Z. L., Hu W. (2023). Ultrafast and Low-Power 2D Bi_2_ O_2_ Se Memristors for Neuromorphic Computing Applications. Nano Lett..

[352] Xie J., Afshari S., Sanchez
Esqueda I. (2022). Hexagonal Boron Nitride (h-BN) Memristor
Arrays for Analog-Based Machine Learning Hardware. Npj 2D Mater. Appl..

[353] Ren Y., De-Eknamkul C., Sun F., Ramezani M., Gonzalez G., Huang W., Schwab J. H., Wilson M. N., Engler A. J., Kuzum D., Cubukcu E. (2025). Trionic All-Optical
Biological Voltage
Sensing via Quantum Statistics. Nat. Photonics.

[354] Li Y., Qian Q., Zhu X., Li Y., Zhang M., Li J., Ma C., Li H., Lu J., Zhang Q. (2020). Recent Advances
in Organic-Based Materials for Resistive Memory Applications. InfoMat.

[355] Luo X., Ming J., Gao J., Zhuang J., Fu J., Ren Z., Ling H., Xie L. (2022). Low-Power Flexible Organic Memristor
Based on PEDOT:PSS/Pentacene Heterojunction for Artificial Synapse. Front. Neurosci..

[356] van de Burgt Y., Lubberman E., Fuller E. J., Keene S. T., Faria G. C., Agarwal S., Marinella M. J., Alec Talin A., Salleo A. (2017). A Non-Volatile Organic Electrochemical
Device as a Low-Voltage Artificial Synapse for Neuromorphic Computing. Nat. Mater..

[357] Lobosco A., Lubrano C., Rana D., Montes V. R., Musall S., Offenhäusser A., Santoro F. (2024). Enzyme-Mediated Organic
Neurohybrid Synapses. Adv. Mater..

[358] Gkoupidenis P., Schaefer N., Garlan B., Malliaras G. G. (2015). Neuromorphic
Functions in PEDOT:PSS Organic Electrochemical Transistors. Adv. Mater..

[359] Ye C., Peng Q., Li M., Luo J., Tang Z., Pei J., Chen J., Shuai Z., Jiang L., Song Y. (2012). Multilevel
Conductance Switching of Memory Device through Photoelectric Effect. J. Am. Chem. Soc..

[360] Wan Q., Sharbati M. T., Erickson J. R., Du Y., Xiong F. (2019). Emerging Artificial
Synaptic Devices for Neuromorphic Computing. Adv. Mater. Technol..

[361] Zhang F., Zhang H., Krylyuk S., Milligan C. A., Zhu Y., Zemlyanov D. Y., Bendersky L. A., Burton B. P., Davydov A. V., Appenzeller J. (2019). Electric-Field Induced Structural Transition in Vertical
MoTe2- and Mo1-xWxTe2-Based Resistive Memories. Nat. Mater..

[362] Yan X., Qian J. H., Sangwan V. K., Hersam M. C. (2022). Progress
and Challenges
for Memtransistors in Neuromorphic Circuits and Systems. Adv. Mater..

[363] Sun K., Chen J., Yan X. (2021). The Future of Memristors: Materials
Engineering and Neural Networks. Adv. Funct.
Mater..

[364] Yan X., Zhang L., Yang Y., Zhou Z., Zhao J., Zhang Y., Liu Q., Chen J. (2017). Highly Improved Performance
in Zr0.5Hf0.5O2 Films Inserted with Graphene Oxide Quantum Dots Layer
for Resistive Switching Non-Volatile Memory. J. Mater. Chem. C.

[365] Wang T., Meng J., Zhou X., Liu Y., He Z., Han Q., Li Q., Yu J., Li Z., Liu Y., Zhu H., Sun Q., Zhang D. W., Chen P., Peng H., Chen L. (2022). Reconfigurable Neuromorphic
Memristor
Network for Ultralow-Power Smart Textile Electronics. Nat. Commun..

[366] Organic core-sheath nanowire artificial synapses with femtojoule energy consumption. 10.1126/sciadv.1501326. PMC4928881 27386556

[367] Tzouvadaki I., Gkoupidenis P., Vassanelli S., Wang S., Prodromakis T. (2023). Interfacing Biology and Electronics
with Memristive Materials. Adv. Mater..

[368] Tuchman Y., Mangoma T. N., Gkoupidenis P., Van De Burgt Y., John R. A., Mathews N., Shaheen S. E., Daly R., Malliaras G. G., Salleo A. (2020). Organic Neuromorphic
Devices: Past, Present, and Future Challenges. MRS Bull..

[369] Mariano A., Bovio C. L., Criscuolo V., Santoro F. (2022). Bioinspired Micro- and Nano-Structured Neural Interfaces. Nanotechnology.

[370] Cucchi M., Kleemann H., Tseng H., Ciccone G., Lee A., Pohl D., Leo K. (2021). Directed Growth of Dendritic Polymer
Networks for Organic Electrochemical Transistors and Artificial Synapses. Adv. Electron. Mater..

[371] Janzakova K., Ghazal M., Kumar A., Coffinier Y., Pecqueur S., Alibart F. (2021). Dendritic Organic Electrochemical
Transistors Grown by Electropolymerization for 3D Neuromorphic Engineering. Adv. Sci..

[372] Petrauskas L., Cucchi M., Grüner C., Ellinger F., Leo K., Matthus C., Kleemann H. (2022). Nonlinear
Behavior of Dendritic Polymer Networks for Reservoir Computing. Adv. Electron. Mater..

[373] Terenzi, L. ; Gao, Z. ; Ravandeh, M. ; Fedele, C. ; Klausen, L. H. ; Bovio, C. L. ; Priimagi, A. ; Santoro, F. Engineering Lipid-Based Pop-up Conductive Interfaces with PEDOT:PSS and Light-Responsive Azopolymer Films. Adv. Healthc. Mater. 2024, 2303812. 10.1002/adhm.202303812 39126173

[374] Seok H., Lee D., Son S., Choi H., Kim G., Kim T. (2024). Beyond von Neumann Architecture: Brain-Inspired Artificial
Neuromorphic Devices and Integrated Computing. Adv. Electron. Mater..

[375] Sun, H. ; Tian, H. ; Hu, Y. ; Cui, Y. ; Chen, X. ; Xu, M. ; Wang, X. ; Zhou, T. Bio-Plausible Multimodal Learning with Emerging Neuromorphic Devices. Adv. Sci. 2024, 11, 2406242. 10.1002/advs.202406242. PMC11615814 39258724

[376] Zhang F., Li C., Li Z., Dong L., Zhao J. (2023). Recent Progress in Three-Terminal Artificial Synapses Based on 2D
Materials: From Mechanisms to Applications. Microsyst. Nanoeng..

[377] Han H., Yu H., Wei H., Gong J., Xu W. (2019). Recent Progress
in Three-Terminal Artificial Synapses: From Device to System. Small.

[378] Seo, J. ; Brezzo, B. ; Liu, Y. ; Parker, B. D. ; Esser, S. K. ; Montoye, R. K. ; Rajendran, B. ; Tierno, J. A. ; Chang, L. ; Modha, D. S. ; Friedman, D. J. A 45nm CMOS Neuromorphic Chip with a Scalable Architecture for Learning in Networks of Spiking Neurons. In 2011 IEEE Custom Integrated Circuits Conference (CICC); 2011; pp 1–4. 10.1109/CICC.2011.6055293.

[379] Ribar L., Sepulchre R. (2019). Neuromodulation of Neuromorphic Circuits. IEEE Trans. Circuits Syst. Regul. Pap..

[380] Sheng J., Park E. J., Shong B., Park J.-S. (2017). Atomic
Layer Deposition of an Indium Gallium Oxide Thin Film for Thin-Film
Transistor Applications. ACS Appl. Mater. Interfaces.

[381] Chai X., Jiang J., Zhang Q., Hou X., Meng F., Wang J., Gu L., Zhang D. W., Jiang A. Q. (2020). Nonvolatile
Ferroelectric Field-Effect Transistors. Nat.
Commun..

[382] Kirubasankar B., Won Y. S., Adofo L. A., Choi S. H., Kim S. M., Kim K. K. (2022). Atomic and Structural Modifications
of Two-Dimensional Transition Metal Dichalcogenides for Various Advanced
Applications. Chem. Sci..

[383] Yu Y., Ma Q., Ling H., Li W., Ju R., Bian L., Shi N., Qian Y., Yi M., Xie L., Huang W. (2019). Small-Molecule-Based Organic Field-Effect
Transistor
for Nonvolatile Memory and Artificial Synapse. Adv. Funct. Mater..

[384] Shao L., Zhao Y., Liu Y. (2021). Organic Synaptic Transistors:
The Evolutionary Path from Memory Cells to the Application of Artificial
Neural Networks. Adv. Funct. Mater..

[385] Paulsen B. D., Tybrandt K., Stavrinidou E., Rivnay J. (2020). Organic Mixed Ionic-Electronic Conductors. Nat. Mater..

[386] Nezakati T., Seifalian A., Tan A., Seifalian A. M. (2018). Conductive
Polymers: Opportunities and Challenges in Biomedical Applications. Chem. Rev..

[387] Friedlein J. T., McLeod R. R., Rivnay J. (2018). Device Physics
of Organic
Electrochemical Transistors. Org. Electron..

[388] Khodagholy D., Rivnay J., Sessolo M., Gurfinkel M., Leleux P., Jimison L. H., Stavrinidou E., Herve T., Sanaur S., Owens R. M., Malliaras G. G. (2013). High Transconductance
Organic Electrochemical Transistors. Nat. Commun..

[389] Belleri P., Pons i Tarrés J., McCulloch I., Blom P. W. M., Kovács-Vajna Z.
M., Gkoupidenis P., Torricelli F. (2024). Unravelling the Operation of Organic Artificial Neurons
for Neuromorphic Bioelectronics. Nat. Commun..

[390] Yang C., Tu D., Ruoko T., Gerasimov J. Y., Wu H., Harikesh P. C., Massetti M., Stoeckel M., Kroon R., Müller C., Berggren M., Fabiano S. (2022). Low-Power/High-Gain
Flexible Complementary Circuits Based on Printed Organic Electrochemical
Transistors. Adv. Electron. Mater..

[391] Huang W., Zhang H., Lin Z., Hang P., Li X. (2024). Transistor-Based Synaptic Devices
for Neuromorphic Computing. Crystals.

[392] Jang H., Biswas S., Lang P., Bae J.-H., Kim H. (2024). Organic Synaptic Transistors: Biocompatible
Neuromorphic Devices
for in-Vivo Applications. Org. Electron..

[393] Kim M.-K., Kim I.-J., Lee J.-S. (2021). Oxide Semiconductor-Based
Ferroelectric Thin-Film Transistors for Advanced Neuromorphic Computing. Appl. Phys. Lett..

[394] Jiang X., Shi C., Wang Z., Huang L., Chi L. (2024). Healthcare Monitoring
Sensors Based on Organic Transistors: Surface/Interface
Strategy and Performance. Adv. Mater..

[395] Strakosas, X. ; Bongo, M. ; Owens, R. M. The Organic Electrochemical Transistor for Biological Applications. J. Appl. Polym. Sci. 2015, 132 (15), 10.1002/app.41735.

[396] Burr G. W., Shelby R. M., Sebastian A., Kim S., Kim S., Sidler S., Virwani K., Ishii M., Narayanan P., Fumarola A., Sanches L. L., Boybat I., Le Gallo M., Moon K., Woo J., Hwang H., Leblebici Y. (2017). Neuromorphic Computing Using Non-Volatile Memory. Adv. Phys. X.

[397] Matrone G. M., Bruno U., Forró C., Lubrano C., Cinti S., Van De Burgt Y., Santoro F. (2023). Electrical and Optical Modulation of a PEDOT:PSS-Based
Electrochemical Transistor for Multiple Neurotransmitter-Mediated
Artificial Synapses. Adv. Mater. Technol..

[398] Keene S. T., Lubrano C., Kazemzadeh S., Melianas A., Tuchman Y., Polino G., Scognamiglio P., Cinà L., Salleo A., Van De Burgt Y., Santoro F. (2020). A Biohybrid Synapse with Neurotransmitter-Mediated
Plasticity. Nat. Mater..

[399] Harikesh P. C., Yang C.-Y., Tu D., Gerasimov J. Y., Dar A. M., Armada-Moreira A., Massetti M., Kroon R., Bliman D., Olsson R., Stavrinidou E., Berggren M., Fabiano S. (2022). Organic Electrochemical
Neurons and
Synapses with Ion Mediated Spiking. Nat. Commun..

[400] Bruno U., Rana D., Ausilio C., Mariano A., Bettucci O., Musall S., Lubrano C., Santoro F. (2024). An Organic
Brain-Inspired Platform with Neurotransmitter Closed-Loop Control,
Actuation and Reinforcement Learning. Mater.
Horiz..

[401] Ausilio C., Lubrano C., Rana D., Matrone G. M., Bruno U., Santoro F. (2024). Concealing Organic Neuromorphic Devices
with Neuronal-Inspired Supported Lipid Bilayers. Adv. Sci. Weinh. Baden-Wurtt. Ger..

[402] Maraj J. J., Schafer E. A., Mansour M. M., Hussein E. A., Berryman J., Klavon E., Rivnay J., Sarles S. A. (2025). Highly
Resistive Biomembranes Coupled to Organic Transistors Enable Ion-Channel
Mediated Neuromorphic Synapses. Adv. Electron.
Mater..

[403] Rana D., Kim C., Wang M., Cicoira F., Santoro F. (2024). Tissue-like Interfacing
of Planar Electrochemical Organic
Neuromorphic Devices. Neuromorphic Comput. Eng..

[404] Liu X., Zeng Z. (2022). Memristor Crossbar
Architectures for Implementing Deep
Neural Networks. Complex Intell. Syst..

[405] Li Y., Su K., Chen H., Zou X., Wang C., Man H., Liu K., Xi X., Li T. (2023). Research Progress of
Neural Synapses Based on Memristors. Electronics.

[406] Shi Y., Ananthakrishnan A., Oh S., Liu X., Hota G., Cauwenberghs G., Kuzum D. (2022). A Neuromorphic Brain
Interface Based on RRAM Crossbar Arrays for High Throughput Real-Time
Spike Sorting. IEEE Trans. Electron Devices.

[407] Zeng F., Li S., Yang J., Pan F., Guo D. (2014). Learning Processes
Modulated by the Interface Effects in a Ti/Conducting
Polymer/Ti Resistive Switching Cell. RSC Adv..

[408] van de Burgt Y., Melianas A., Keene S. T., Malliaras G., Salleo A. (2018). Organic Electronics for Neuromorphic
Computing. Nat. Electron..

[409] Li H., Wang S., Zhang X., Wang W., Yang R., Sun Z., Feng W., Lin P., Wang Z., Sun L., Yao Y. (2021). Memristive Crossbar
Arrays for Storage and Computing Applications. Adv. Intell. Syst..

[410] Leydecker T., Herder M., Pavlica E., Bratina G., Hecht S., Orgiu E., Samorì P. (2016). Flexible Non-Volatile
Optical Memory Thin-Film Transistor Device with over 256 Distinct
Levels Based on an Organic Bicomponent Blend. Nat. Nanotechnol..

[411] Erokhin V., Berzina T., Gorshkov K., Camorani P., Pucci A., Ricci L., Ruggeri G., Sigala R., Schüz A. (2012). Stochastic Hybrid 3D Matrix: Learning and Adaptation
of Electrical Properties. J. Mater. Chem..

[412] Abderrahmane, N. ; Miramond, B. Information Coding and Hardware Architecture of Spiking Neural Networks. In 2019 22nd Euromicro Conference on Digital System Design (DSD); IEEE: Kallithea, Greece, 2019; pp 291–298. 10.1109/DSD.2019.00050.

[413] Neftci E. O., Augustine C., Paul S., Detorakis G. (2017). Event-Driven
Random Back-Propagation: Enabling Neuromorphic Deep Learning Machines. Front. Neurosci..

[414] Cramer B., Billaudelle S., Kanya S., Leibfried A., Grübl A., Karasenko V., Pehle C., Schreiber K., Stradmann Y., Weis J., Schemmel J., Zenke F. (2022). Surrogate
Gradients for Analog Neuromorphic Computing. Proc. Natl. Acad. Sci. U. S. A..

[415] Indiveri, G. ; Linares-Barranco, B. ; Hamilton, T. J. ; Schaik, A. V. ; Etienne-Cummings, R. ; Delbruck, T. ; Liu, S.-C. ; Dudek, P. ; Häfliger, P. ; Renaud, S. ; Schemmel, J. ; Cauwenberghs, G. ; Arthur, J. ; Hynna, K. ; Folowosele, F. ; Saighi, S. ; Serrano-Gotarredona, T. ; Wijekoon, J. ; Wang, Y. ; Boahen, K. Neuromorphic Silicon Neuron Circuits. Front. Neurosci. 2011, 5, 10.3389/fnins.2011.00073. PMC3130465 21747754

[416] Van Schaik A. (2001). Building Blocks for Electronic Spiking
Neural Networks. Neural Netw..

[417] Donati E., Payvand M., Risi N., Krause R., Indiveri G. (2019). Discrimination
of EMG Signals Using a Neuromorphic
Implementation of a Spiking Neural Network. IEEE Trans. Biomed. Circuits Syst..

[418] Guo W., Fouda M. E., Eltawil A. M., Salama K. N. (2023). Efficient Training
of Spiking Neural Networks with Temporally-Truncated Local Backpropagation
through Time. Front. Neurosci..

[419] Neftci E. O., Mostafa H., Zenke F. (2019). Surrogate
Gradient
Learning in Spiking Neural Networks: Bringing the Power of Gradient-Based
Optimization to Spiking Neural Networks. IEEE
Signal Process. Mag..

[420] Gao H., He J., Wang H., Wang T., Zhong Z., Yu J., Wang Y., Tian M., Shi C. (2023). High-Accuracy Deep
ANN-to-SNN Conversion Using Quantization-Aware Training Framework
and Calcium-Gated Bipolar Leaky Integrate and Fire Neuron. Front. Neurosci..

[421] Saponati M., Vinck M. (2023). Sequence Anticipation and Spike-Timing-Dependent
Plasticity Emerge from a Predictive Learning Rule. Nat. Commun..

[422] Kugele A., Pfeil T., Pfeiffer M., Chicca E. (2020). Efficient
Processing of Spatio-Temporal Data Streams With Spiking Neural Networks. Front. Neurosci..

[423] Zhu S., Xie T., Lv Z., Leng Y.-B., Zhang Y.-Q., Xu R., Qin J., Zhou Y., Roy V. A. L., Han S.-T. (2024). Hierarchies
in Visual Pathway: Functions and Inspired Artificial Vision. Adv. Mater..

[424] Corrado F., Bruno U., Prato M., Carella A., Criscuolo V., Massaro A., Pavone M., Muñoz-García A. B., Forti S., Coletti C., Bettucci O., Santoro F. (2023). Azobenzene-Based
Optoelectronic Transistors for Neurohybrid Building Blocks. Nat. Commun..

[425] Matrone G. M., Van Doremaele E. R. W., Surendran A., Laswick Z., Griggs S., Ye G., McCulloch I., Santoro F., Rivnay J., Van De Burgt Y. (2024). A Modular
Organic Neuromorphic Spiking Circuit for Retina-Inspired Sensory Coding
and Neurotransmitter-Mediated Neural Pathways. Nat. Commun..

[426] Kwon S. M., Cho S. W., Kim M., Heo J. S., Kim Y.-H., Park S. K. (2019). Environment-Adaptable Artificial
Visual Perception Behaviors Using a Light-Adjustable Optoelectronic
Neuromorphic Device Array. Adv. Mater..

[427] Jo C., Kim J., Kwak J. Y., Kwon S. M., Park J. B., Kim J., Park G., Kim M., Kim Y., Park S. K. (2022). Retina-Inspired
Color-Cognitive Learning via Chromatically Controllable Mixed Quantum
Dot Synaptic Transistor Arrays. Adv. Mater..

[428] Yuan R., Tiw P. J., Cai L., Yang Z., Liu C., Zhang T., Ge C., Huang R., Yang Y. (2023). A Neuromorphic
Physiological Signal Processing System Based on VO2Memristor for Next-Generation
Human-Machine Interface. Nat. Commun..

[429] Broccard F. D., Joshi S., Wang J., Cauwenberghs G. (2017). Neuromorphic
Neural Interfaces: From Neurophysiological Inspiration to Biohybrid
Coupling with Nervous Systems. J. Neural Eng..

[430] Mavoori J., Jackson A., Diorio C., Fetz E. (2005). An Autonomous
Implantable Computer for Neural Recording and Stimulation in Unrestrained
Primates. J. Neurosci. Methods.

[431] Berger T. W., Song D., Chan R. H. M., Marmarelis V. Z., LaCoss J., Wills J., Hampson R. E., Deadwyler S. A., Granacki J. J. (2012). A Hippocampal Cognitive
Prosthesis: Multi-Input, Multi-Output
Nonlinear Modeling and VLSI Implementation. IEEE Trans. Neural Syst. Rehabil. Eng..

[432] Wilson M. N., Thunemann M., Liu X., Lu Y., Puppo F., Adams J. W., Kim J.-H., Ramezani M., Pizzo D. P., Djurovic S., Andreassen O. A., Mansour A. A., Gage F. H., Muotri A. R., Devor A., Kuzum D. (2022). Multimodal Monitoring of Human Cortical Organoids Implanted in Mice
Reveal Functional Connection with Visual Cortex. Nat. Commun..

[433] Park J. H., Tan J. S. Y., Wu H., Dong Y., Yoo J. (2020). 1225-Channel
Neuromorphic Retinal-Prosthesis SoC With Localized Temperature-Regulation. IEEE Trans. Biomed. Circuits Syst..

[434] Matsukatova A. N., Prudnikov N. V., Kulagin V. A., Battistoni S., Minnekhanov A. A., Trofimov A. D., Nesmelov A. A., Zavyalov S. A., Malakhova Y. N., Parmeggiani M., Ballesio A., Marasso S. L., Chvalun S. N., Demin V. A., Emelyanov A. V., Erokhin V. (2023). Combination of Organic-Based
Reservoir Computing and
Spiking Neuromorphic Systems for a Robust and Efficient Pattern Classification. Adv. Intell. Syst..

[435] Gupta I., Serb A., Khiat A., Zeitler R., Vassanelli S., Prodromakis T. (2016). Real-Time Encoding and Compression
of Neuronal Spikes by Metal-Oxide Memristors. Nat. Commun..

[436] Khoyratee, F. ; Nishikawa, S. M. ; Zhongyue, L. ; Kim, S. H. ; Saighi, S. ; Fujii, T. ; Ikeuchi, Y. ; Aihara, K. ; Levi, T. Biomimetic Spiking Neural Network (SNN) Systems for ‘In Vitro’ Cells Stimulation. In 2019 IEEE International Symposium on Circuits and Systems (ISCAS); IEEE: Sapporo, Japan, 2019; pp 1–5. 10.1109/ISCAS.2019.8702407.

[437] Juzekaeva E., Nasretdinov A., Battistoni S., Berzina T., Iannotta S., Khazipov R., Erokhin V., Mukhtarov M. (2019). Coupling Cortical
Neurons through Electronic Memristive
Synapse. Adv. Mater. Technol..

[438] Serb A., Corna A., George R., Khiat A., Rocchi F., Reato M., Maschietto M., Mayr C., Indiveri G., Vassanelli S., Prodromakis T. (2020). Memristive Synapses Connect Brain and Silicon Spiking
Neurons. Sci. Rep..

[439] Benfenati V., Toffanin S., Bonetti S., Turatti G., Pistone A., Chiappalone M., Sagnella A., Stefani A., Generali G., Ruani G., Saguatti D., Zamboni R., Muccini M. (2013). A Transparent Organic
Transistor Structure for Bidirectional
Stimulation and Recording of Primary Neurons. Nat. Mater..

[440] Luo S., Shao L., Ji D., Chen Y., Wang X., Wu Y., Kong D., Guo M., Wei D., Zhao Y., Liu Y., Wei D. (2023). Highly Bionic Neurotransmitter-Communicated Neurons
Following Integrate-and-Fire Dynamics. Nano
Lett..

[441] Balamur R., Eren G. O., Kaleli H. N., Karatum O., Kaya L., Hasanreisoglu M., Nizamoglu S. (2024). A Retina-Inspired
Optoelectronic Synapse Using Quantum Dots for Neuromorphic Photostimulation
of Neurons. Adv. Sci..

[442] Cai H., Ao Z., Tian C., Wu Z., Liu H., Tchieu J., Gu M., Mackie K., Guo F. (2023). Brain Organoid
Reservoir Computing for Artificial Intelligence. Nat. Electron..

[443] Van Den Biggelaar R.
H. G. A., Hoefnagel M. H. N., Vandebriel R. J., Sloots A., Hendriksen C. F. M., Van Eden W., Rutten V. P. M. G., Jansen C. A. (2021). Overcoming Scientific
Barriers in the Transition from *in Vivo* to Non-Animal
Batch Testing of Human and Veterinary Vaccines. Expert Rev. Vaccines.

[444] Vázquez-Guardado A., Yang Y., Bandodkar A. J., Rogers J. A. (2020). Recent Advances in Neurotechnologies
with Broad Potential
for Neuroscience Research. Nat. Neurosci..

[445] Wang W., Jiang Y., Zhong D., Zhang Z., Choudhury S., Lai J.-C., Gong H., Niu S., Yan X., Zheng Y., Shih C.-C., Ning R., Lin Q., Li D., Kim Y.-H., Kim J., Wang Y.-X., Zhao C., Xu C., Ji X., Nishio Y., Lyu H., Tok J. B.-H., Bao Z. (2023). Neuromorphic Sensorimotor Loop Embodied by Monolithically
Integrated, Low-Voltage, Soft e-Skin. Science.

[446] Donati E., Valle G. (2024). Neuromorphic Hardware for Somatosensory
Neuroprostheses. Nat. Commun..

[447] Raspopovic S., Valle G., Petrini F. M. (2021). Sensory
Feedback
for Limb Prostheses in Amputees. Nat. Mater..

[448] Buccelli S., Bornat Y., Colombi I., Ambroise M., Martines L., Pasquale V., Bisio M., Tessadori J., Nowak P., Grassia F., Averna A., Tedesco M., Bonifazi P., Difato F., Massobrio P., Levi T., Chiappalone M. (2019). A Neuromorphic Prosthesis to Restore
Communication in Neuronal Networks. iScience.

[449] Boi, F. ; Moraitis, T. ; De Feo, V. ; Diotalevi, F. ; Bartolozzi, C. ; Indiveri, G. ; Vato, A. A Bidirectional Brain-Machine Interface Featuring a Neuromorphic Hardware Decoder. Front. Neurosci. 2016, 10, 10.3389/fnins.2016.00563. PMC5145890 28018162

[450] Chiappalone M., Cota V. R., Carè M., Di Florio M., Beaubois R., Buccelli S., Barban F., Brofiga M., Averna A., Bonacini F., Guggenmos D. J., Bornat Y., Massobrio P., Bonifazi P., Levi T. (2022). Neuromorphic-Based
Neuroprostheses for Brain Rewiring: State-of-the-Art and Perspectives
in Neuroengineering. Brain Sci..

[451] Sun Y., Wang Y., Yuan Q. (2024). Artificial
Nociceptor Based on Temperature
Responsive of Synaptic Transistor for Electronic Skin. Appl. Mater. Today.

[452] Shim H., Sim K., Ershad F., Yang P., Thukral A., Rao Z., Kim H.-J., Liu Y., Wang X., Gu G., Gao L., Wang X., Chai Y., Yu C. (2019). Stretchable Elastic
Synaptic Transistors
for Neurologically Integrated Soft Engineering Systems. Sci. Adv..

[453] Gerasimova S. A., Lebedeva A. V., Fedulina A., Koryazhkina M., Belov A. I., Mishchenko M. A., Matveeva M., Guseinov D., Mikhaylov A. N., Kazantsev V. B., Pisarchik A. N. (2021). A Neurohybrid
Memristive System for Adaptive Stimulation of Hippocampus. Chaos Solitons Fractals.

[454] De Salvo A., Rondelli F., Di Lauro M., Tomassini A., Greco P., Stieglitz T., Fadiga L., Biscarini F. (2023). Organic Electronics
Circuitry for In Situ Real-Time Processing of Electrophysiological
Signals. Adv. Mater. Interfaces.

[455] Ienca M., Andorno R. (2017). Towards New Human Rights
in the Age
of Neuroscience and Neurotechnology. Life Sci.
Soc. Policy.

[456] Bublitz J. C., Merkel R. (2014). Crimes Against Minds: On Mental Manipulations,
Harms and a Human Right to Mental Self-Determination. Crim. Law Philos..

[457] Gilbert F., Ienca M., Cook M. (2023). How I Became
Myself
after Merging with a Computer: Does Human-Machine Symbiosis Raise
Human Rights Issues?. Brain Stimulat..

[458] Helbing D., Ienca M. (2024). Why Converging Technologies Need
Converging International Regulation. Ethics
Inf. Technol..

[459] Magee P., Ienca M., Farahany N. (2024). Beyond Neural Data:
Cognitive Biometrics and Mental Privacy. Neuron.

[460] Ienca M., Malgieri G. (2022). Mental Data Protection and the GDPR. J. Law Biosci..

[461] Hendriks S., Li X., Grady C., Kim S. Y. (2024). Public
Views on Whether the Use of Pharmaceutical Neuroenhancements Should
Be Allowed. Neurology.

[462] Bruns A., Winkler E. C. (2024). Dynamic Consent:
A Royal Road to
Research Consent?. J. Med. Ethics.

[463] Kaye J., Whitley E. A., Lund D., Morrison M., Teare H., Melham K. (2015). Dynamic Consent: A
Patient Interface
for Twenty-First Century Research Networks. Eur. J. Hum. Genet..

[464] Soekadar S., Chandler J., Ienca M., Bublitz C. (2021). On The Verge
of the Hybrid Mind. Morals Mach..

[465] Ienca M., Valle G., Raspopovic S. (2025). Clinical Trials
for Implantable Neural Prostheses: Understanding the Ethical and Technical
Requirements. Lancet Digit. Health.

[466] Sai S., Sharma S., Chamola V. (2024). Explainable
AI-Empowered Neuromorphic
Computing Framework for Consumer Healthcare. IEEE Trans. Consum. Electron..

[467] Eke D. O., Bernard A., Bjaalie J. G., Chavarriaga R., Hanakawa T., Hannan A. J., Hill S. L., Martone M. E., McMahon A., Ruebel O., Crook S., Thiels E., Pestilli F. (2022). International Data Governance for Neuroscience. Neuron.

[468] European Parliament . Directorate General for Parliamentary Research Services. The Protection of Mental Privacy in the Area of Neuroscience: Societal, Legal and Ethical Challenges.; Publications Office: LU, 2024.

[469] Bertoni, E. ; Ienca, M. The Privacy and Data Protection Implication of the Use of Neurotechnology and Neural Data from the Perspective of Convention 108+. Council of Europe, June 2024. https://www.coe.int/en/web/data-protection/-/the-privacy-and-data-protection-implication-of-the-use-of-neurotechnology-and-neural-data-from-the-perspective-of-convention-108 (accessed 2025–07–31).

[470] Ienca M., Jotterand F., Elger B. S. (2018). From Healthcare
to Warfare and Reverse: How Should We Regulate Dual-Use Neurotechnology?. Neuron.

[471] Ienca M., Vayena E. (2018). Dual Use in the 21st Century: Emerging
Risks and Global Governance. Swiss Med. Wkly..

